# Development of bifunctional fluorescent probes and their application to α-helix labelling†‡

**DOI:** 10.1039/d5ob00563a

**Published:** 2025-05-21

**Authors:** Laszlo Kondacs, David R. Trentham, Thomas Kampourakis, Alexander J. A. Cobb

**Affiliations:** a Department of Chemistry, King's College London 7 Trinity Street London SE1 1DB UK andre.cobb@kcl.ac.uk; b Randall Centre for Cell and Molecular Biophysics and British Heart Foundation Centre of Research Excellence UK; c College of Medicine, University of Kentucky 900 S. Limestone Street William R. Willard Medical Education Building MN 150 Lexington KY 40536-0298 USA thomas.kampourakis@uky.edu

## Abstract

The site selective modification of proteins and peptides is an important venture when it comes to the study of biological systems, such as in the determination of viable pharmacological targets and in the understanding of biomolecular mechanisms. In this paper we report on the development of novel bifunctional probes that allow for the unambiguous site-specific labelling of short peptides for spectroscopic measurements as demonstration of our future intentions to introduce these as functional labels for the study of protein dynamincs *in situ*. The symmetrical nature and bifunctional attachments of these probes to their targets significantly reduces their orientational disorder (*i.e.* ‘dye diffusion’), improving the accuracy and interpretation of established methods to study protein dynamics such as fluorescence polarization and Foerster Resonance Energy Transfer (FRET) measurements. In addition to solving a problem which has led to previous probes giving convoluted data owing to atropisomeric diastereoisomerism upon binding, we also introduce bio-orthogonal attachment groups that circumvent some of the drawbacks associated with the traditional labelling chemistries of thiol-reactive groups. These novel probes will be useful tools for future bulk and single-molecule spectroscopic experiments.

## Abstract 2

Novel bifunctional probes for α-helix labelling is described. These probes both solve an existing problem with current probes of this class, as well as introduce new orthogonality that will assist with the future study of dynamic proteins *in situ*.

## Graphical abstract

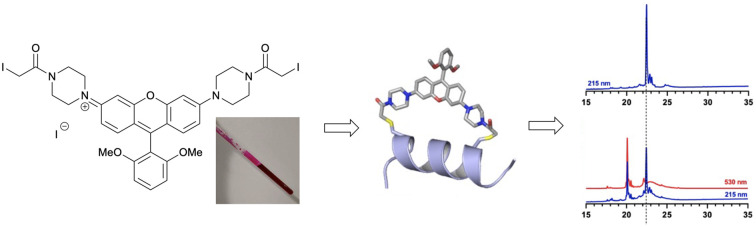

The site-directed labelling of proteins with dipolar fluorescent probes is a powerful technique to study their structural and conformational dynamics with high spatial (nm-scale) and temporal resolution (microsecond scale).^1–5^ However, traditional probe chemistry uses single attachment points using mainly thiol- or amino-reactive chemical groups which introduces a phenomenon known as “dye diffusion”, where uncertainty about the orientation of the excitation and emission dipoles of the fluorophore with respect to the three-dimensional structure of its target protein (Fig. 1) significantly limits the analysis and interpretation of spectroscopic experiments, such as Foerster-Resonance Energy Transfer (FRET), electron paramagnetic resonance spectroscopy (EPR) and fluorescence polarization (FP).^6^ The introduction of bifunctional probes with *two* attachment sites significantly reduces dye diffusion, making it easier to distinguish different structural and conformational states.^7–11^

**Fig. 1:**
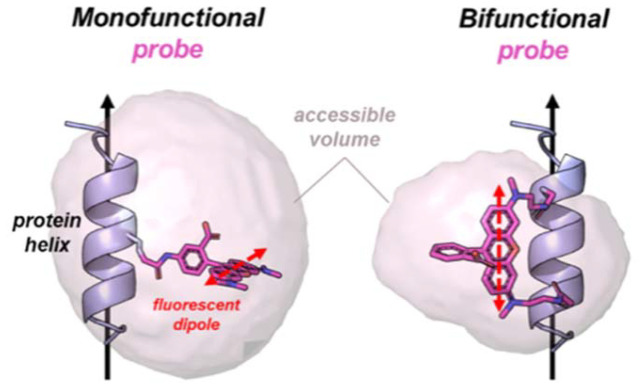
Bifunctional attachment reduces fluorescent probe diffusion. Models of a mono-functional (left) and bifunctional rhodamine probe (right) attached to a single protein helix. The calculated accessible space for each probe around its attachment point(s) is indicated by pink transparent surface.

However, both commercially available and custom-synthesized thiol-reactive bifunctional fluorescent probes for protein labelling – almost exclusively based on a rhodamine backbone – are problematic because they can attach to the target protein in two different orientations. Whilst the unbound rhodamine BSR 1 is achiral, upon binding to the protein it becomes atropisomeric owing to the ability of the σC–Ar bond to rotate, meaning that the aryl group can adopt one of two positions (Fig. 2A). Whilst computational modelling predicts that upon binding there is no barrier to rotation between these atropisomers, it does show that there is a slight preference for one of them, which is assumed to result from the favored conformation being able to interact with solvent to a greater degree.^12^ To further complicate matters, each atropisomer has a slightly different conformation with respect to their attachment points.^13^ As a result, the measured fluorescence data becomes convoluted, as more than one signal is obtained. Moreover, a previous study showed that the functional effects of incorporating a bifunctional rhodamine along the C-helix of skeletal troponin C in isolated skeletal muscle fibers depends upon the diastereoisomer.^14^ This is likely due to different interactions of the rhodamine probe with the myofilament proteins within the two diastereoisomers.

**Fig. 2:**
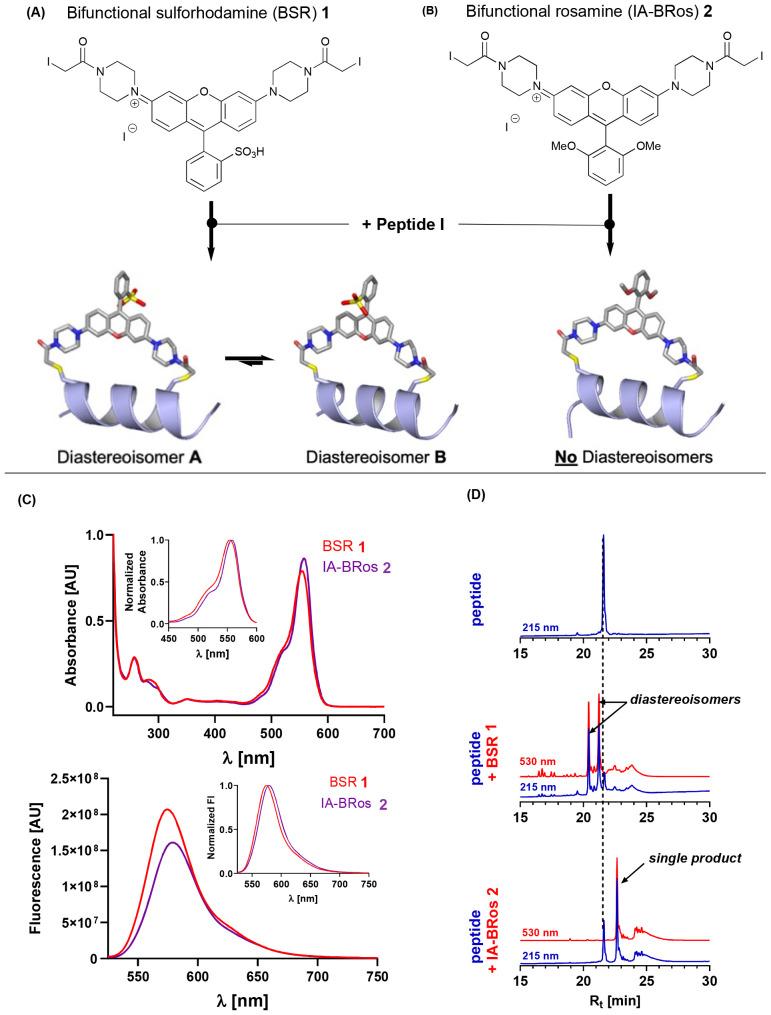
Bifunctional rosamine probes. (A) Chemical structure of Bifunctional Sulforhodamine (BSR, 1) and atomic models of the diastereoisomers after attachment to a protein helix. (B) Chemical structure of Bifunctional Rosamine (BRos, 2) and atomic model of the protein helix-dye complex. (C) Absorbance (top) and emission spectrum of 10 mmol L^−1^ BSR (red) and BRos (purple). Insets show normalized spectra. (D) RP-HPLC traces of unconjugated cardiac troponin C C-helix peptide (top), 1 : 1 reaction mixture of peptide with BSR 1 (middle) and 1 : 1 reaction mixture with IA-BRos 2 (bottom). Blue and red lines indicate absorbance at 215 nm and 530 nm, respectively. See ESI‡ for sequence information of Peptide I.

In order to circumvent this problem, we identified the thiol-reactive symmetric rosamine system 2 whose synthesis is outlined in the ESI.‡ Owing to its symmetric nature, conjugation of the iodoacetamido-bifunctional rosamine (IA-BRos) to its target protein is predicted to give only a single product. This rosamine shows comparable spectroscopic properties to the corresponding commercially-available bifunctional sulforhodamine (BSR) (Fig. 2C) with only a slight bathochromic shift of the emission maximum by about 5 nm. This increases its utility for the use as a probe in already established fluorescence polarization experiments.^9,15^ Moreover, the probe exhibits sufficient water solubility at neutral pH (>500 mmol L^−1^) which allows conjugation to target proteins under near native conditions.

To demonstrate the utility of this new rosamine, we used a synthetic peptide corresponding to the C-helix of cardiac troponin C (cTnC) carrying two cysteines placed at i and i + 7 positions as a model system (peptide I – NPTP**C**ELQEMI**C**EVDEDGS). Bifunctional rhodamine probes attached to this helix on troponin C have been widely used in fluorescence polarization experiments to study both myofilament function and its regulation.^16–18^ First, we reacted the synthetic peptide with the commercially available BSR probe 1 in a 1 : 1 ratio. BSR almost quantitatively reacted with the peptide giving two main products, which were readily separable by reverse phase high performance liquid chromatography (Fig. 2D). Mass spectrometry analysis showed that both peaks have the same mass, indicating that the two products are diastereoisomers in a 5 : 4 ratio.

As predicted, incubation of IA-BRos 2 with the synthetic peptide gave a *single* reaction product corresponding to the crosslinked peptide-dye complex, which was further confirmed by mass spectrometry (see ESI‡). IA-BRos 2 showed slightly lower reactivity than BSR and a shift to the right in the RP-HPLC chromatogram, indicating an increase in product hydrophobicity, likely due to the substitution of the sulfonic group for two methoxy-moieties.

Although thiol-reactive labelling of proteins is well established, this methodology is limited to proteins without intrinsic cysteine residues to allow site-specifically attachment of probes to the engineered target site. Alternatively, native cysteines must be replaced by non-reactive residues (*E.g.* serine or alanine) before engineering of the labelling site, which might compromise the protein structure or function, or both.^19^ Moreover, small molecule effectors or drugs might depend on cysteine-reactivity as part of their mechanism of action (*e.g.* Levosimendan binding to cTnC),^20^ limiting the utility of thiol-reactive probes.

As the introduction of unnatural amino acids such as azido and cyano-functionalized residues is well-known and readily accessible,^21^ we designed bifunctional rosamine probes that could be deployed in such a manner, relying on click chemistry partners to achieve this. Two fluorophores – bisazido-BRos 3 and bispropargyl-BRos 4 was therefore designed and synthesized. Both fluorophores showed similar absorbance and fluorescence spectra compared to the thiol-reactive fluorophore IA-BRos 2 (Fig. 3B). Interestingly, bispropargyl-BRos 4 showed an about ten-fold weaker fluorescence than fluorophores 2 and 3.

**Fig. 3:**
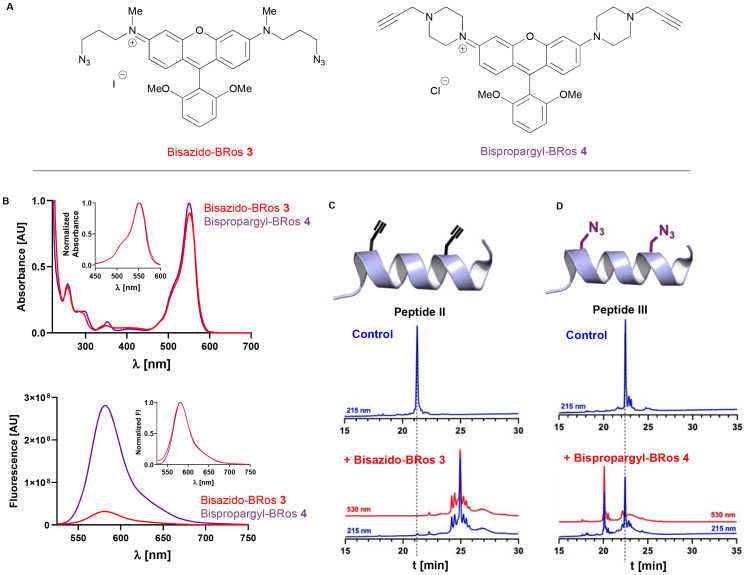
Bio-orthogonal bifunctional rosamine probes. (A) Structures of clickable fluorophores bisazido-BRos 3 and bispropargyl-BRos 4. (B) Absorbance (top) and emission spectra of 10 mmol L^−1^ bisazido-BRos 3 (purple) and bispropargyl-BRos 4 (red). Insets show normalized spectra. (C) RP-HPLC traces of unconjugated cardiac troponin C C-helix peptide containing propargyl-glycine residues (top) and after reaction with equimolar bisazido-BRos 3 (bottom). (D) RP-HPLC traces of unconjugated cardiac troponin C C-helix peptide containing azido-alanine residues (top) and after reaction with equimolar bispropargyl-BRos 4 (bottom). Blue and red lines indicate absorbance at 215 nm and 530 nm, respectively. See ESI‡ for sequence information of peptides II and II.

Static quenching *via* dye stacking can decrease the quantum yields of fluorescent probes.^22^ We therefore measured the absorbance and excitation spectrum of bispropargyl-BRos 4 at different concentrations. The fluorescence intensity of this at its emission maximum is linear related to its concentration between 0–20 mmol L^−1^, which is significantly lower than the concentrations used for the experiments (10 mmol L^−1^). In good agreement, its absorbance spectrum shows a characteristic increase of the shoulder at about 520 nm, indicating fluorophore stacking, only at concentrations greater than 25 mmol L^−1^. This suggests that the low fluorescence quantum yield of the probe is not due to probe stacking. We estimated its extinction coefficient at the maximum by linear regression of the concentration-dependent absorbance to about 135 000 M^−1^ cm^−1^, which is in excellent agreement with previously published results (see ESI‡).^7^

Next, we measured the emission spectrum of rosamines 2, 3 and 4 at 10 mmol L^−1^ in DMSO, methanol and aqueous 10 mmol L^−1^ Tris-HCl pH 7. Surprisingly, the fluorescence maxima exhibits a hypsochromatic shift by about 10–15 nm going from the aprotic polar (DMSO) to a protic polar solvent (MeOH or water). The blue shift of the emission maximum is accompanied by an increase in fluorescence intensity for IA-BRos 2 and bisazido-BRos 3 by about 50% and 100% respectively. In contrast, bispropargyl-BRos 4 fluorescence increases five-fold in aqueous buffer, suggesting a significantly larger solvatochromism (see ESI‡).

Viscosity has also been shown to affect fluorescence quantum yields of fluorophores by restricting molecular motions that can cause non-radiative decay pathways such as rotation and other local motions.^23^ We therefore measured the fluorescence of 10 mmol L^−1^ IA-BRos 2, bisazido-BRos 3 and bispropargyl-BRos 4 in the presence of increasing concentrations of glycerol. These showed an initial small increase in fluorescence with glycerol concentrations <50% (v/v), followed by a drop in fluorescence for glycerol concentrations >50% (v/v), although the effect was stronger for IA-BRos 2 than for bisazido-Bros 3. In contrast, bispropargyl-BRos 4 showed about a 300% increase in fluorescence intensity with increasing glycerol content, suggesting that deactivating internal molecular motions are partially responsible for its lower quantum yield.

Similarly, the protonation state of fluorophores has been shown to strongly affect their fluorescence quantum yield and we tested this idea by measuring the fluorescence intensity of all three probes in aqueous buffers with different pH (Fig. 2C).^24^ The fluorescence of bisazido-BRos 3 was largely unaffected by the pH, and IA-BRos 2 showed an increase by about 30% with a p*K*_a_ of about 5.5. Strikingly, bispropargyl-BRos 4 shows an about five-fold increase in the fluorescence with decreasing pH with an p*K*_a_ of about 4.

Taken together this suggests that solvatochromism, protonation state and internal motion-based deactivation pathways might be responsible for the lower quantum yield of bispropargyl-BRos **4**. This might make it a useful probe for studying protein conformational changes because conjugation to target proteins might change the local chemical environment of the probe and therefore modulate its fluorescent properties.

To test the reactivity of the fluorophores we designed custom-synthesized peptides corresponding to the C-helix of cTnC carrying either a pair of propargyl-glycine or azido-alanine residues and analyzed their reaction with the fluorophores using RP-HPLC and mass spectrometry. Incubating a 1 : 1 mixture of bisazido-BRos 3 with the propargyl-containing peptide in the presence of Cu^+^ resulted in full conversion of the peptide with a single major reaction product in the RP-HPLC chromatogram (Fig. 3C). Mass spectrometry analysis was consistent with a 1 : 1 peptide–fluorophore complex. In contrast, the propargyl-BRos 4 only showed about 50% conversion of the azido-alanine containing peptide with a single major product under the same condition (Fig. 3D). The lower reactivity of propargyl-Bros 4 is likely due to its more rigid piperazine moieties, which results in lower conformational flexibility of the reactive linkers and therefore less favorable thermodynamics, but other factors may well also be at play – including solubility, and also the accessibility of the azidoalanine group to adopt the correct orientation for successful 1,3-dipolar cycloaddition (particularly with respect to reaction of the second functionality).

Understanding the orientations and local motions of fluorophore labels with respect to their designed attachment points on the surface of their target proteins is crucial for accurately interpreting both fluorescence polarization and FRET measurements.^25^ We therefore created structural models of the three fluorophores attached to the C-helix of cTnC using molecular dynamics and simulated probe motion by generating random conformers (Fig. 4A). Probe orientations were quantified by the dihedral angle between the vector joining the Cα-atoms of the amino acids to which the probes were attached and the vector going through the C4 and C5 carbon of the xanthylium group (Fig. 4B). The latter is roughly parallel to the emission dipole of the rosamine probes.^13^ The largest probe motion was observed for Azido-BRos with an interquartile range of dihedral angles of about 15° and a maximum range of >50°. In contrast, PrG-Bros 4 showed the smallest orientational disorder with interquartile and maximum range of 8° and 18°, respectively. The predicted probe motion for IA-BRos 2 was intermediate to bisazido-BRos 3 and bispropargyl-BRos 4 with an interquartile range of about 12°. The latter is in excellent agreement with previous fluorescence polarization measurements using BSR 1 and estimating that the local probe movement (‘wobble’) is restricted within a semi-cone angle of about 10°.^14^

**Fig. 4:**
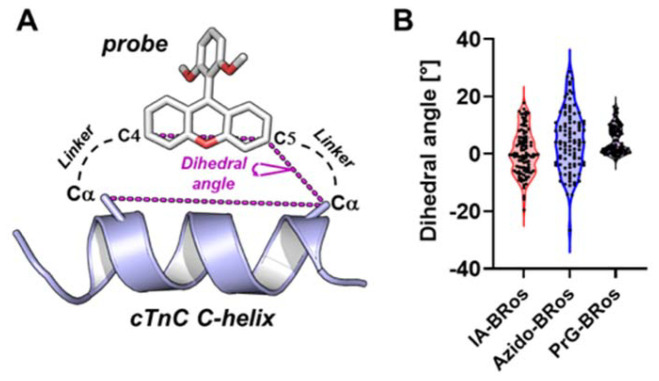
Effect of linker structures on conformational flexibility of rosamine probes. (A) Conformational flexibility of the probes attached to the C-helix of cTnC was quantified by the dihedral angle between the vector joining Cα-atoms of the amino acids used for probe attachment and the vector going through the C4 and C5 carbons of the probe's xanthylium moiety. (B) Distribution of dihedral angles for IA-Bros 2, bisazido-BRos 3 and bispropargyl-BRos 4 attached to the cTnC C-helix peptide for 100 randomly generated conformers.

Although restricted probe motion *via* stiffer linkers is desirable for spectroscopic measurements, previous experiments have shown that more flexible attachments of the fluorescent probe to the protein can have less effects on its native function.^14^ Flexibility within the linkers likely allows the protein to better accommodate the probe without compromising its structure–function activity.

In conclusion, we have developed a new tool kit for the site-specific modification of proteins with bifunctional probes that solve the problems of atropisomeric binding, as well as labelling orthogonality, which will be useful for future fluorescence polarization and FRET measurements in the study of protein conformational and structural dynamics.

## Conflicts of interest

There are no conflicts to declare.

## Supplementary Material

OB-023-D5OB00563A-s001

## Data Availability

The data supporting this article have been included as part of the ESI‡ including NMR spectra, mass spectroscopy, and IR spectra for all synthesized compounds including 2, 3 and 4, peptide labelling and all fluorescence spectra.
