# Supplementary material for: Development of bifunctional fluorescent probes and their application to α-helix labelling
Source: Org Biomol Chem. 2025 May 21;23(24):5788–92. doi: 10.1039/d5ob00563a (PMC12121550; doi:10.1039/d5ob00563a)
Supplement: OB-023-D5OB00563A-s001 [file OB-023-D5OB00563A-s001.pdf]

Supplementary Information (SI) for Organic & Biomolecular Chemistry.
This journal is © The Royal Society of Chemistry 2025

Supporting information

Development of Bifunctional Fluorescent Probes and their Application to a-Helix Labelling

Laszlo Kondacs[1,2], David R. Trentham[2], Thomas Kampourakis*[,3] and Alexander J. A. Cobb*[,1]

[1]Department of Chemistry, King's College London, 7 Trinity Street, London SE1 1DB, UK

[2]Randall Centre for Cell and Molecular Biophysics and British Heart Foundation Centre of Research Excellence,

[3]College of Medicine, University of Kentucky, 900 S. Limestone Street, William R. Willard Medical Education Building, MN 150, Lexington KY, USA 40536-0298

Contents

Chemical Synthesis 1
Synthetic Scheme for Rosamine Probes 2 and 3 2
Synthetic Scheme for Rhodamine Probe 4 3
NMR Spectra, Mass Spec and IR Data 11
Supporting Information Biology 38
Supporting Information Figures 39
Supporting Information Tables 41
Supporting Information References 45

Chemical Synthesis

All commercially available reagents and solvents were obtained from Sigma-Aldrich, Apollo Scientific, Alfa Aesar, Fluorochem, VWR or Fischer Scientific and were used without further purification. Melting points were recorded on Stuart SMP30 melting point apparatus and are uncorrected. Infrared spectra were recorded in the range 4000 – 600 cm^{-1} using a Shimadzu IRAffinity-1S FTIR Spectrophotometer as a thin film. Bruker Ascend 400 at 400 MHz for ^{1}H spectra or at 100 MHz for ^{13}C spectra. Chemical shifts (δ) are quoted in parts per million (ppm) using the abbreviations: s: singlet; d: doublet; dd: double of dublets; t: triplet; quart: quartet; quint: quintet. Signals that could not be interpreted were designated multiplets (m) or broad (br). Coupling constants *J* are quoted in Hz. High resolution accurate mass measurements were collected by King's College London mass spec service with an Acquity UPLC coupled to a Xevo G2-XS QTOF. UV – VIS measurements were carried out by PerkinElmer LAMBDA 465 UV/Vis Spectrophotometer in quartz cuvettes. Fluorescence was measured with an Agilent Cary Eclipse Fluorescence Spectrophotometer in quartz cuvettes. VWR silica gel 60 (40-63 micron) was used for column chromatography; the samples were pre-absorbed onto silica 60 (40-63 micron). Teledyne ISCO CombiFlash Rf+ was used for flash chromatography. Air and moisture sensitive reactions were carried out in oven dried glassware under a nitrogen atmosphere. Microwave reactions were carried out in a Biotage Initiator+ microwave synthesizer. Thin layer chromatography was performed on VWR silica gel 60 F254. Thin layer chromatography results were analysed by UV lamp (254 nm), ninhydrin stain (amine, amide content) and/or ceric ammonium molybdate stain.

Synthetic Scheme for Rosamine Probes 2 and 3

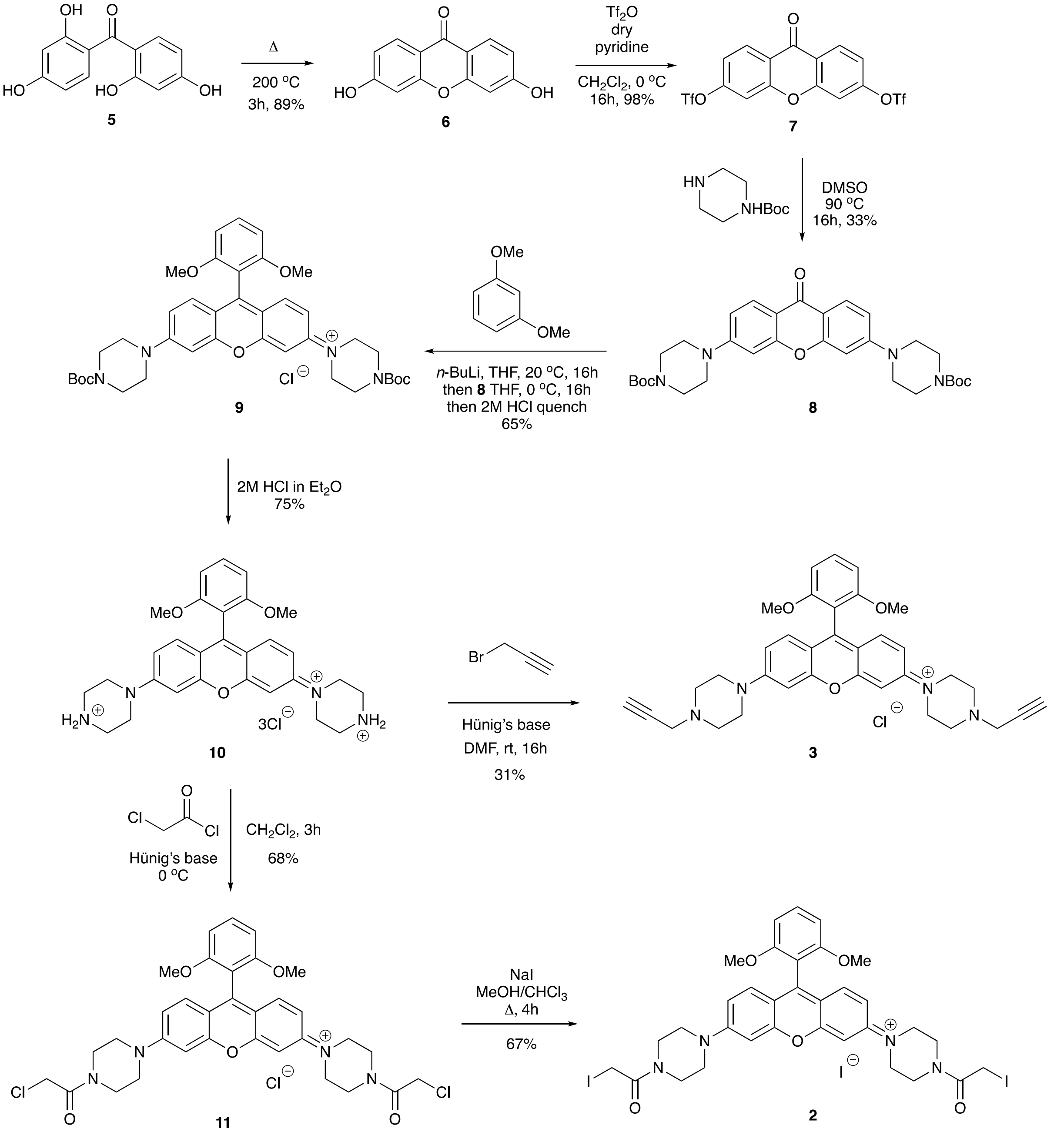

Synthetic Scheme for Rhodamine Probe 4

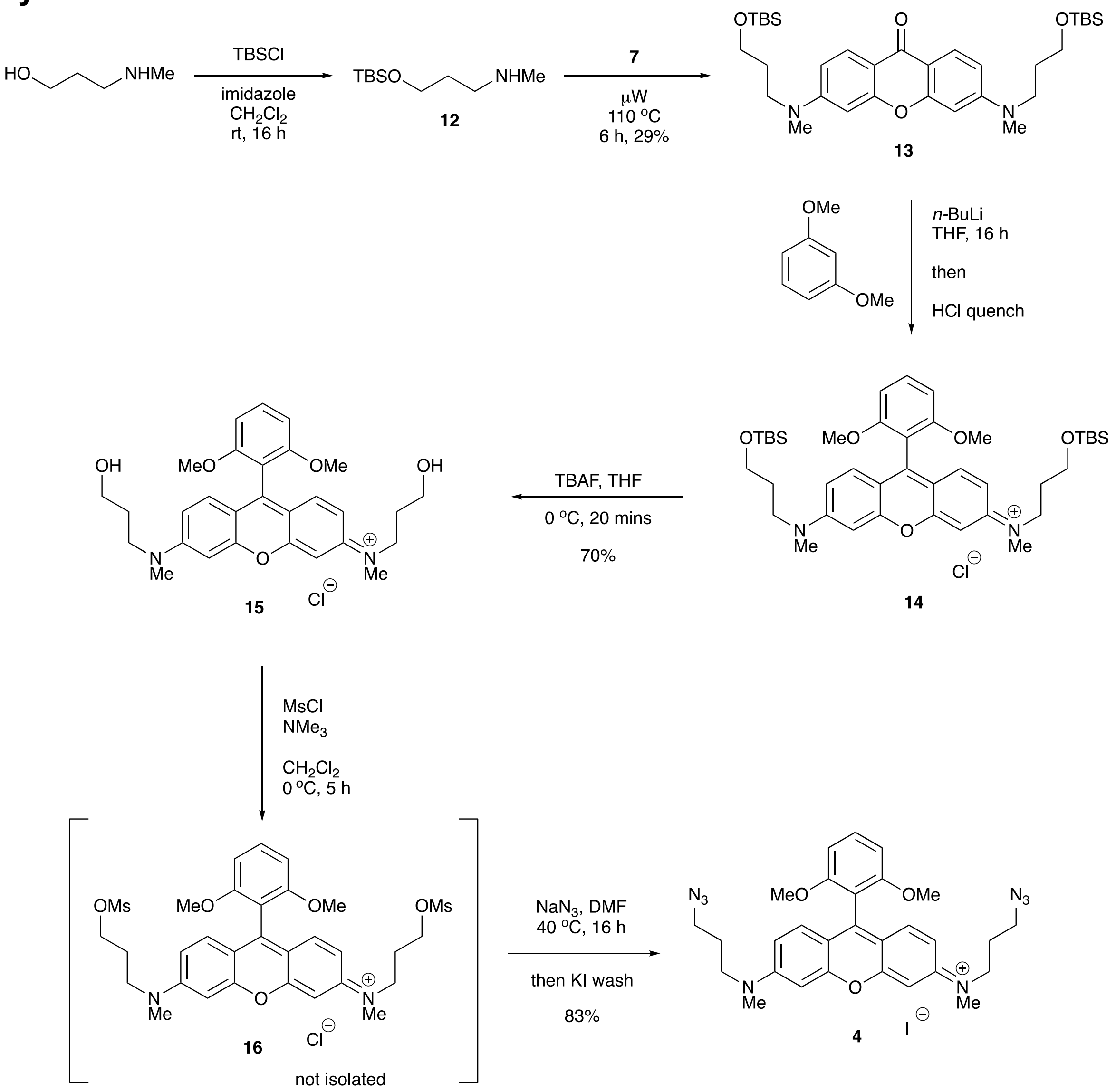

3,6-dihydroxy-9H-xanthen-9-one **6**[1]

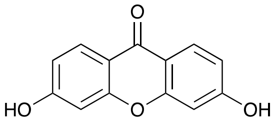

A suspension of 2,2',4,4'tetrahydroxybenzophenonein **5** (1.60 g, 6.80 mmol) in distilled water (20 mL) was stirred and heated for 3 h at 200 °C in a microwave reactor. After the reaction mixture was cooled down to room temperature, it was filtered and washed with hot water (80 - 90 °C) to give the pure product as a peach-coloured fluffy solid (1.38 g, 89%). Mp: 358 °C (decomposed); ^{1}H NMR (400 MHz, DMSO-d$_6$) δ_H 10.85 (2H, br s , 3-OH), 7.99 (2H, d, *J* 8.8, 4-H), 6.86 (2H, dd, *J* 8.8, 2.4, 2-H), 6.82 (2H, d, *J* 2.0, 1-H); ^{13}C NMR (101 MHz, DMSO-d$_6$) δ_c 174.4 (9-C), 163.8 (3-C), 157.9 (4a-C), 128.2 (1-C), 114.5 (9a-C), 114.1 (2-C), 102.6 (4-C); IR : v_{max}/cm^{-1} 3127 (O-H), 1612 (C=O), 1247 (C-O); HRMS : Found (M+H)$^+$ 229.0515. Calcd. for $C_5H_{11}ON_6$: (M+H)$^+$ 228.0495.

9-oxo-9H-xanthene-3,6-diyl bis(trifluoromethanesulfonate) **7**[1]

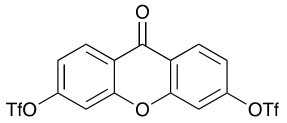

To a solution of 3,6-dihydroxy-9H-xanthen-9-one **6** (3.32 g, 14.6 mmol) in dichloromethane (60 mL) at 0 °C was added pyridine (146.0 mmol, 11.8 mL) and the mixture was stirred for 15 minutes. Triflic anhydride (5.88 mL, 35.0 mmol) was added dropwise to the reaction mixture at 0 °C, and the resulting solution stirred overnight at room temperature. After the reaction was complete, it was quenched with 1M aqueous HCl solution (30 mL). The organic phase was washed with brine (2 x 40 mL), dried over $MgSO_4$, and evaporated under reduced pressure to give the product as butter-coloured crystals (7.06 g, 98%). Mp: 113 - 115 °C). ^{1}H NMR (400 MHz, $CDCl_3$) : δ_H 8.47 (2H, d, *J* 8.8 Hz, 1-H), 7.52 (2H, d, *J* 2.4 Hz, 4-H), 7.38 (2H, dd, *J*=8.8 Hz, *J*=2.4 Hz, 2-H); ^{13}C NMR (101 MHz, $CDCl_3$) δ_c 174.5 (9-C), 156.6 (4a-C), 153.4 (3-C), 129.6 (1-C), 121.4 (9a-C), 118.7 (J_{C-F}=322.2, 12-C), 118.2 (2-C), 111.4 (4-C); ^{19}F NMR (376 MHz, $CDCl_3$) δ_F 72.5 (12-F); v_{max}/cm^{-1} 1664 (C=O), 1611 (C=O), 1420 (S=O), 1206 (C-O), 1134 (S=O), 1119 (C-F); HRMS: Found (M+H)$^+$ 492.9470. Calcd. for $C_{15}H_6F_6O_8S_2$: (M+H)$^+$ 492.9487.

Di-tert-butyl 4,4'-(9-oxo-9H-xanthene-3,6-diyl)bis(piperazine-1-carboxylate) **8**[2]

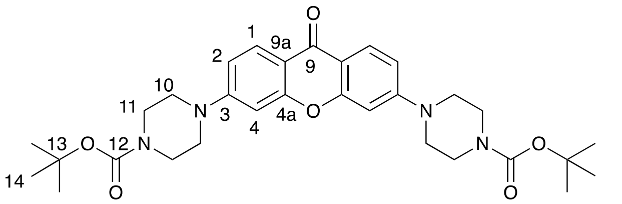

To the solution of 9-oxo-9H-xanthene-3,6-diyl bis(trifluoromethanesulfonate) **7** (3.00 g, 6.1 mmol) in DMSO (30 mL), was added Boc-piperazine (11.35 g 61.0 mmol) in one portion and the resulting mixture heated overnight at 90 °C. After the reaction was complete, it was cooled to room temperature and quenched with saturated aqueous Na_2CO_3 solution (30 mL). The precipitate was filtered, washed with water and dried to give the crude product as yellow solid (8.00 g). The crude was purified by recrystallization from ethyl acetate and hexane solvent mixture to give the title product as yellow crystals (1.15 g, 33%). Mp: 217 - 219 °C; ^{1}H NMR (400 MHz, $CDCl_3$) δ_H 8.16 (2H, d, *J* 8.8, 1-H), 6.89 (2H, dd, *J* 8.8, 2.0, 2-H), 6.69 (2H, *J* 2.0, 4-H), 3.63 (8H, t, *J* 4.8, 11-H), 3.40 (8H, t, *J* 5.2, 10-H), 1.51 (18H, s, 14-H). ^{13}C NMR (101 MHz, $CDCl_3$) δ_c 175.1 (9-C), 158.1 (4a-C), 155.0 (3-C), 154.6 (12-C), 127.8 (1-C), 114.0 (9a-C), 111.6 (2-C), 100.3 (4-C), 80.3 (13-C), 47.4 (10-C), 43.0 (br s, 11-C), 28.4 (14-C); v_{max}/cm^{-1} 1676 (C=O), 1606 (C=O), 1233 (C-O); HRMS (Found (M+H)$^+$ 565.3098. Calcd. for $C_{31}H_{41}O_6N_4$: (M+H)$^+$ 565.3026.).

4-(tert-butoxycarbonyl)-1-(6-(4-(tert-butoxycarbonyl)piperazin-1-yl)-9-(2,6-dimethoxyphenyl)-3H-xanthen-3-ylidene)piperazin-1-ium chloride **9**[2]

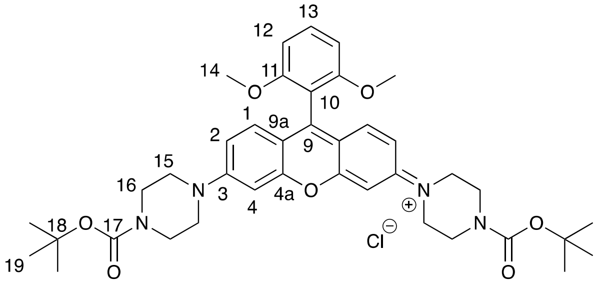

To the solution of 1,3-dimethoxybenzene (0.49 g, 3.50 mmol) in dry tetrahydrofuran (5 mL) at 0 °C under a nitrogen atmosphere, was added dropwise 2.5 M solution of *n*-butyl lithium in tetrahydrofuran (1.4 mL, 3.50 mmol), and the resulting solution stirred overnight at room temperature to give suspension A, which was then added to a solution of di-tert-butyl 4,4'-(9-oxo-9H-xanthene-3,6-diyl)bis(piperazine-1-carboxylate) **8** (0.40 g, 0.71 mmol) in dry tetrahydrofuran (10 mL) at 0°C under nitrogen atmosphere with a canule. The orange solution generated was stirred overnight at room temperature. After the reaction was complete, it was quenched with 2 M HCl (1.8 mL), and dichloromethane added (30 mL). The organic layer was washed with water (30 mL), brine (30 mL), dried over $MgSO_4$, filtered and the solvent evaporated under reduced pressure to give the crude product as a dark purple solid (0.87 g). This crude compound was purified by column chromatography (eluent: dichloromethane – methanol, 0% - 8%) to give the pure product as a dark solid with a golden shine (0.33 g, 65%). Mp: 215 °C (decomposed); ^{1}H NMR (400 MHz, $CDCl_3$) δ_H 7.50 (1H, t, *J* 8.4, 13-H), 7.31 (2H, d, *J* 9.6, 1-H), 7.23 (2H, dd, *J* 9.6, 2.4 Hz, 2-H), 7.14 (2H, d, *J*=2.4 Hz, 4-H), 6.73 (2H, d, *J* 8.8, 12-H), 3.81 (8H, br m, 15-H), 3.66 (8H, m, 16-H), 3.64 (6H, s, 14-H), 1.47 (18H, s, 19-H); ^{13}C NMR (101 MHz, $CDCl_3$) δ_c 158.4 (3-C), 157.5 (11-C), 157.1 (4a-C), 155.4 (9-C), 154.5 (17-C), 132.6 (13-C), 132.1 (1-C), 115.6 (9a-C), 115.1(2-C), 108.5 (10-C), 104.2 (10-C), 97.9 (4-C), 80.7 (18-C), 56.0 (14-C), 47.1 (15-C), 43.1 (16-C), 28.4 (19-C); v_{max}/cm^{-1} 1701 (C=O), 1342 (C-N), 1220 (C-O); HRMS (Found $(M+H)^+$ 686.3690. Calcd. for $C_{39}H_{50}O_7N_4$: $(M+H)^+$ 686.3669.).

1-(9-(2,6-dimethoxyphenyl)-6-(piperazin-1-ium-1-yl)-3H-xanthen-3-ylidene)piperazine-1,4-diium chloride **10**

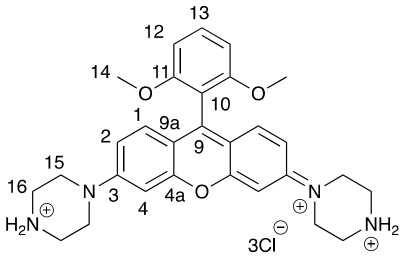

4-(tert-butoxycarbonyl)-1-(6-(4-(tert-butoxycarbonyl)piperazin-1-yl)-9-(2,6-dimethoxyphenyl)-3H-xanthen-3-ylidene)piperazin-1-ium chloride **9** (0.34, 0.47 mmol) was dissolved in a solution of 2M HCl in diethyl ether (10 mL), and stirred overnight. After the reaction was complete, the precipitate was collected by filtration, and washed with diethyl ether (20 mL) to give the product as a red solid (0.21 g, 75%). Due to its slight hygroscopic nature it was dissolved in water, and freeze dried for easier handling. Mp: 259 °C (melted and decomposed); ^{1}H NMR (400 MHz, MeOD-*d*$_4$) δ_H 7.68 (1H, t, *J* 8.8, 13-H), 7.47 (2H, d, *J* 10.0, 2-H), 7.40 (2H, m, 1-H), 7.38 (2H, m, 4-H), 6.97 (2H, d, 8.8, 12-H), 4.11 (8H, t, *J* 5.2, 15-H), 3.70 (6H, s, 14-H), 3.49 (8H, t, *J* 5.2, 16-H); ^{13}C NMR (101 MHz, MeOD-*d*$_4$) δ_c 159.8 (3-C), 158.8 (11-C), 158.5 (4a-C), 134.1 (13-C), 133.2 (4-C), 117.0 (9a-C), 116.5 (2-C), 109.3 (10-C), 105.3 (12-C), 99.2 (1-C), 56.4 (14-C), 45.0 (15-C), 43.9 (16-C); v_{max}/cm^{-1} 3365 (N-H), 1580 (N-H), 1240 (C-O), 1180 (C-N); HRMS: Found $(M+H)^{2+}$ 243.1334. Calcd. for $C_{29}H_{34}O_3N_4$: $(M+H)^{2+}$ 243.1310; Found $(M)^+$ 485.2576. Calcd. For $C_{29}H_{33}O_3N_4$: $(M)^+$ 485.2547.

4-(2-chloroacetyl)-1-(6-(4-(2-bromo/chloroacetyl)piperazin-1-yl)-9-(2,6-dimethoxyphenyl)-3H-xanthen-3-ylidene)piperazin-1-ium chloride **11**

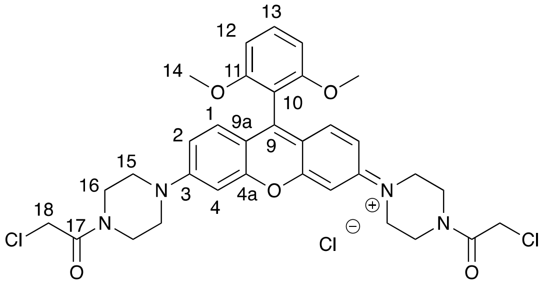

To a solution of compound **10** (0.20 g, 0.36 mmol) in dichloromethane (15 mL) and diisopropylethylamine (0.49 mL, 2.9 mmol) at 0 °C, was added chloroacetyl chloride (0.12 mL, 0.14mmol) dropwise. The resulting mixture was stirred for 3 hours whilst warming up to room temperature. After the reaction was over, a mixture of chloroform and methanol (9:1 ratio) (45 mL) and 0.5 M HCl (45 mL) were added to the reaction mixture. The aqueous phase was extracted with a further mixture of chloroform and methanol (9:1 ratio) (2 x 45 mL). The combined organic layers were washed with saturated $NaHCO_3$ solution (2 x 30 mL), brine (2 x 30 mL), dried over $MgSO_4$, fitered and concentrated under reduced pressure. The crude material (0.64 g) was purified by flash chromatography (eluent: dichloromethane – methanol 0 – 20%) to give the title compound as a dark purple solid (164 mg, 68%). Mp: 123 °C (melted and decomposed); ^{1}H NMR (400 MHz, $CDCl_3$) δ_H 7.54 (1H, t, *J* 8.4, 13-H), 7.33 (2H, d, *J* 9.6, 1-H), 7.22 (2H, dd, *J* 2.4, *J* 9.2, 2-H), 7.15 (2H, d, *J* 2.4, 4-H), 6.74 (2H, d, *J* 7.6, 12-H), 4.24 (4H, s, 18-H), 4.04 (4H, m, 15a-H), 3.92 (4H, m, 16a-H), 3.83 (4H, m, 16b-H), 3.80 (4H, m, 15b-H), 3.65 (6H, s, 14-H). ^{13}C NMR (101 MHz, $CDCl_3$) δ_C 165.9 (17-C), 158.4 (4a-C), 157.5 (11-C), 157.1 (3-C), 156.0 (9-C), 132.7 (13-C), 132.2 (1-C), 115.7 (9a-C), 115.1 (2-C), 108.3 (10-C), 104.2 (12-C), 98.0 (4-C), 56.0 (14-C), 47.2 (15a-C), 46.4 (15b-C), 45.2 (16a-C), 41.5 (16b-C), 41.2 (18-C); v_{max}/cm^{-1} 1643 (C=O), 1015 (C-O), 779 (C-Cl); HRMS: Found $(M)^+$ 637.1987. Calcd. For $C_{33}H_{35}O_5N_4Cl_2$: $(M)^+$ 637.1979.

1-(9-(2,6-dimethoxyphenyl)-6-(4-(2-iodoacetyl)piperazin-1-yl)-3H-xanthen-3-ylidene)-4-(2-iodoacetyl)piperazin-1-ium iodide **2**

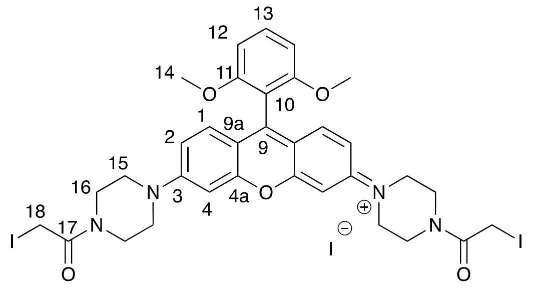

To the solution of compound **20** (0.14 g, 0.21 mmol) in methanol (4 mL) and chloroform (9 mL) mixture sodium iodide (1.56 g, 10.4 mmol) was added and the mixture was heated at reflux temperature for 4 hours under nitrogen atmosphere. After the reaction was complete, it was diluted with chloroform and methanol mixture (100 mL, ratio: 4:1). The solution was washed with 5% sodium ascorbate (30 mL), 1 M potassium phosphate buffer (3 x 30 mL, pH=7), and after the addition of methanol (1.5 mL) once more with 1 M potassium phosphate buffer (30 mL). The organic phase was dried over Na_2SO_4 and the solvent was removed under reduced pressure. The residue was triturated with diethyl ether to give the pure product as metallic purple solid (0.13 g, 67%). Mp: 190 °C (melted and decomposed); ^{1}H NMR (400 MHz, $CDCl_3$) δ_H 7.49 (1H, t, *J* 10.8, 13-H), 7.28 (2H, d, *J* 9.2, 1-H), 7.12 (2H, dd, *J* 2.4, 9.2, 2-H), 7.09 (2H, d, *J* 2.0 Hz, 4-H), 6.70 (2H, d, *J* 8.8, 12-H), 3.98 (4H, m, 15a-H), 3.82 (4H, s, 18-H), 3.80 (8H, m, 16-H), 3.73 (4H, m, 15b-H), 3.60 (6H, s, 14-H); ^{13}C NMR (101 MHz, $CDCl_3$) δ_C 167.3 (17-C), 158.4 (4a-C), 157.5 (11-C), 157.0 (3-C), 156.1 (9-C), 132.8 (13-C), 132.2 (1-C), 115.7 (9a-C), 115.1 (2-C), 108.3 (10-C), 104.3 (12-C), 98.1 (4-C), 56.1 (14-C), 46.9 (15a-C), 46.4 (15b-C), 46.2 (16a-C), 41.5 (16b-C), -3.44 (18-C); v_{max}/cm^{-1} 1636 (C=O), 1224 (C-O); HRMS: Found $(M)^+$ 821.0749. Calcd. for $C_{33}H_{35}I_2N_4O_5$: $(M)^+$ 821.0691; UV/VIS (10.7 μM in water and 1 % DMSO): λ_{max}= 553 nm, A_2= 0.97; Fluorescence (1.07 μM in water and 0.1% DMSO): λ_{ex}= 554 nm, $Intensity_{ex}$= 191 a.u.; λ_{em}= 580 nm, $Intensity_{em}$= 197 a.u.

1-(9-(2,6-dimethoxyphenyl)-6-(4-(prop-2-yn-1-yl)piperazin-1-yl)-3H-xanthen-3-ylidene)-4-(prop-2-yn-1-yl)piperazin-1-ium chloride **3**

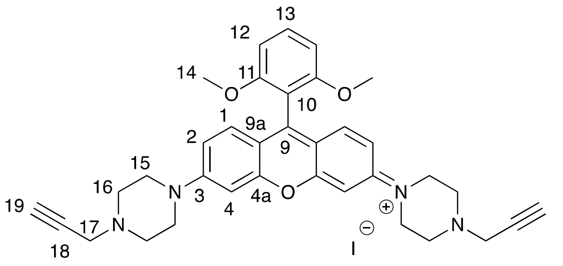

To the solution of compound **10** (0.25 g, 0.44 mmol) in DMF (5 mL) was added N,N-diisopropylethylamine (0.43 mL, 2.49 mmol) and the resulting solution stirred at room temperature for 20 minutes. Propargyl bromide (0.12 mL, 1.07 mmol) was then added and the mixture stirred overnight at room temperature. After the reaction was complete, ethyl acetate (20 mL) and saturated $NaHCO_3$ solution (20 mL) were added, and the aqueous phase washed with a mixture of isopropanol and dichloromethane (5 – 15 mL x 2). The combined organic phases were dried over $MgSO_4$, filtered and evaporated under reduced pressure. The crude product was purified by flash chromatography (eluent: dichloromethane – methanol, 0 – 20 %) to give the pure product as a metallic purple solid (83 mg, 31%). Mp: 189 - 191°C; ^{1}H NMR (400 MHz, $CDCl_3$) δ_H 7.52 (1H, t, *J* 8.4, 13-H), 7.28 (2H, d, *J* 9.2, 1-H), 7.21 (2H, dd, *J* 2.4, 2-H), 7.08 (2H, d, *J* 2.4, 4-H), 6.74 (2H, d, *J* 8.4, 12-H), 3.82 (8H, t, *J* 4.8, 15-H), 3.65 (6H, s, 14-H), 3.38 (4H, d, *J* 2.4, 17-H), 2.76 (8H, t, *J* 4.8, 16-H), 2.26 (2H, t, *J* 2.0, 19-H); ^{13}C NMR (101 MHz, $CDCl_3$) δ_C 158.5 (4a-C), 157.6 (11-C), 157.1 (3-C), 155.1 (9-C), 132.7 (13-C), 132.1 (1-C), 115.5 (9a-C), 115.2 (2-C), 108.6 (10-C), 104.3 (12-C), 97.9 (4-C), 78.0 (18-C), 74.1 (19-C), 56.2 (14-C), 51.5 (16-C), 47.5 (15-C), 46.7 (17-C); ν_{max}/cm^{-1} 3380 (C-H_{alkyne}), 1096 (C-O); HRMS: Found $(M)^+$ 561.2916. Calcd. for $C_{35}H_{37}N_4O_3^+$: $(M)^+$ 561.2860. UV/VIS (10.7 μM in water and 1 % DMSO): λ_{max}= 555 nm, A_2= 0.46; Fluorescence (1.07 μM in water and 0.1% DMSO): λ_{ex}= 555 nm, $Intensity_{ex}$= 70 a.u.; λ_{em}= 581 nm, $Intensity_{em}$= 71 a.u.

3-((*tert*-butyldimethylsilyl)oxy)-N-methylpropan-1-amine **12**

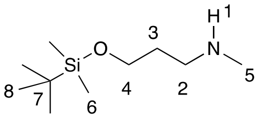

To the solution of 3-(methylamino)propan-1-ol (2.14 mL, 22.0 mmol) in dichloromethane (30 mL) was added TBDMS-chloride (3.73 g, 22.0 mmol) and imidazole (2.09 g, 30.8 mmol), and the resulting solution stirred overnight at room temperature. After this time, the reaction was quenched with water (30 mL), and the aqueous layer was extracted with dichloromethane (3 x 30 mL). The combined organic layers were washed with saturated $NaHCO_3$ solution (30mL), brine (30 mL), dried over $MgSO_4$, filtered and the solvent removed under reduced pressure. The crude material was purified by column chromatography (eluent: ethyl acetate – methanol 10 – 20 % – saturated aqueous NH_3 1%) to give the pure product as a colourless oil (3.23 g, 72%). ^{1}H NMR (400 MHz, $CDCl_3$) δ_H 3.67 (2H t, *J* 6.0, 4-H), 2.66 (2H, t, *J* 7.2, 2-H), 2.42 (3H, s, 5-H), 1.85 (1H, br s, 1-H), 1.70 (2H, quint, *J* 6.8, 3-H), 0.88 (9H, s, 8-H), 0.04 (6-H); ^{13}C NMR (101 MHz, $CDCl_3$) δ_c 61.8 (4-C), 49.4 (2-C), 36.4 (5-C), 32.6 (3-C), 25.9 (8-C), 18.3 (7-C), -5.4 (6-C); ν_{max}/cm^{-1} 3387 (N-H), 1095 (Si-O); HRMS. Found $(M+H)^+$ 204.1824. Calcd. for $C_{10}H_{26}NOSi$: $(M+H)^+$ 204.1778.

3,6-bis((3-((*tert*-butyldimethylsilyl)oxy)propyl)(methyl)amino)-9H-xanthen-9-one **13**

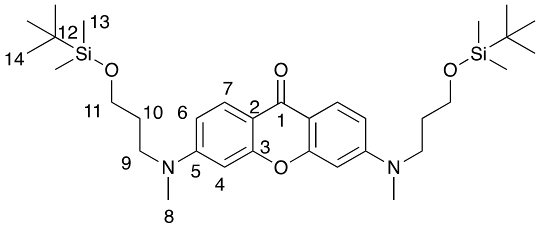

To the solution of 9-oxo-9H-xanthene-3,6-diyl bis(trifluoromethanesulfonate) **7** (1.36 g, 2.8 mmol) in DMSO (14 mL) was added 3-((*tert*-butyldimethylsilyl)oxy)-N-methylpropan-1-amine **12** (2.23 g, 11.0 mmol). The solution was stirred and heated at 110 °C in a microwave reactor for 6 hours. After the reaction was complete, water (30 mL) and dichloromethane (40 mL) were added to the mixture, and the aqueous phase extracted with dichloromethane (2 x 40 mL). The combined organic layers were washed with saturated Na_2CO_3 solution (40mL), brine (40 mL), dried over $MgSO_4$, filtered and the solvent removed under reduced pressure. The crude was purified by column chromatography (eluent: hexane - ethyl acetate 20%) to give the pure product as pale yellow oil (0.48 g, 29%). ^{1}H NMR (400 MHz, $CDCl_3$) δ_H 8.10 (2H, d, *J* 8.8, 7-H), 6.70 (2H, dd, *J* 8.8, 2.4 Hz, 6-H), 6.49 (2H, d, *J* 2.4, 4-H), 3.68 (4H, t, *J* 5.6, 11-H), 3.55 (4H, t, *J* 7.2, 9-H), 3.06 (6H, s, 8-H), 1.82 (4H, quint, *J* 6.0, 10-H), 0.94 (18H, s, 14-H), 0.08 (12H, s, 13-H); ^{13}C NMR (101 MHz, $CDCl_3$) δ_c 175.1 (1-C), 158.3 (2-C), 153.5 (5-C), 127.6 (7-C), 111.8 (3-C), 108.9 (6-C), 96.8 (4-C), 60.1 (11-C). 49.2 (9-C), 38.6 (8-C), 30.1 (10-C), 26.0 (14-C), 18.3 (12-C), -5.4 (13-C); ν_{max}/cm^{-1} 1601 (C=O), 1119 (Si-O); HRMS: Found $(M+H)^+$ 599.3696. Calcd. for $C_{33}H_{54}N_2O_4Si_2$: $(M+H)^+$ 599.3695.

(*E*)-3-((*tert*-butyldimethylsilyl)oxy)-*N*-(6-((3-((*tert*-butyldimethylsilyl)oxy)propyl)(methyl)amino)-9-(2,6-dimethoxyphenyl)-3H-xanthen-3-ylidene)-*N*-methylpropan-1-aminium chloride **14**

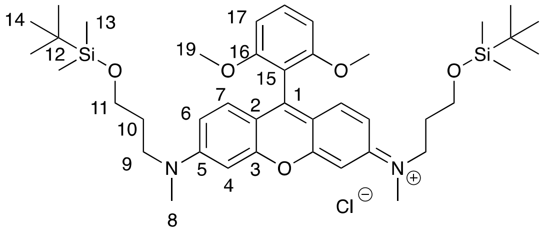

To the solution of 1,3-dimethoxybenzene (1.04 g, 7.51 mmol) in dry tetrahydrofuran (10 mL) at 0°C under nitrogen atmosphere, was added dropwise a 2.5 M solution of *n*-butyl lithium in tetrahydrofuran (3.0 mL, 7.51 mmol). The resulting solution was stirred overnight at room temperature to give a suspension which was added dropwise at 0 °C to a solution of compound **13** (0.90 g, 1.50 mmol) in dry tetrahydrofuran (20 mL) under a nitrogen atmosphere with a cannula. The resulting orange solution was stirred overnight at r.t.. After this time, the reaction was quenched with 2 M HCl (3.9 mL), and dichloromethane added (30 mL). The organic layer was washed with water (60 mL), brine (60 mL), dried over $MgSO_4$, filtered and the solvent was evaporated under reduced pressure to

give the crude product as dark purple oil (1.97 g). The crude material was purified by column chromatography (eluent: dichloromethane – methanol 2 - 6%) to give the pure product as dark solid with golden shine (0.81 g, 71%). Mp: 128 – 130 °C; ^{1}H NMR (400 MHz, $CDCl_3$) δ_H 7.53 (1H, t, *J* 8.4, 18-H), 7.22 (2H, d, *J* 9.6, 7-H), 7.00 (2H, dd, *J* 9.6, 2.4, 6-H), 6.85 (2H, m, 4-H), 6.74 (2H, d, *J* 8.4, 17-H), 3.73 (4H, t, *J* 6.8, 9-H), 3.68 (4H, t, *J* 5.2, 11-H), 3.65 (6H, s, 19-H), 3.31 (6H, s, 8-H), 1.89 (4H, quint, *J* 6.4, 10-H), 0.91 (18H, s, 14-H), 0.06 (12H, s, 13-H); ^{13}C NMR (101 MHz, $CDCl_3$) δ_c 158.0 (3-C), 157.6 (16-C), 156.9 (5-C), 154.7 (1-C), 132.5 (18-C), 131.6 (7-C), 114.5 (2-C), 114.3 (6-C), 108.6 (15-C), 104.2 (17-C), 96.4 (4-C), 59.6 (11-C), 56.1 (19-C), 50.1 (9-C), 39.7 (8-C), 30.2 (10-C), 25.9 (14-C), 18.2 (12-C), -5.4 (13-C); ν_{max}/cm^{-1} 1096 (C-O), 1068 (Si-O); HRMS: Found $(M)^+$ 719.3998. Calcd. for $C_{41}H_{63}N_2O_5Si_2^+$: $(M)^+$ 719.4270.

(*E*)-N-(9-(2,6-dimethoxyphenyl)-6-((3-hydroxypropyl)(methyl)amino)-3H-xanthen-3-ylidene)-3-hydroxy-N-methylpropan-1-aminium chloride **15**

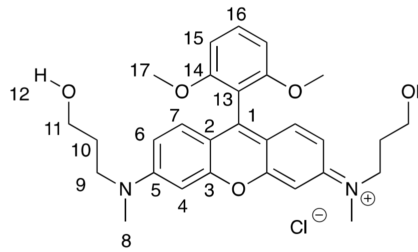

To the solution of (*E*)-3-((*tert*-butyldimethylsilyl)oxy)-*N*-(6-((3-((*tert*-butyldimethylsilyl)oxy)propyl)(methyl)amino)-9-(2,6-dimethoxyphenyl)-3H-xanthen-3-ylidene)-*N*-methylpropan-1-aminium chloride **14** (0.80 g, 1.06 mmol) in THF (10 mL) at 0 °C was added a solution of TBAF in THF (1.11 mL, 1M, 1.11 mmol), and the resulting solution stirred for 20 minutes at that temperature. After the reaction was complete, dichloromethane (10 mL) was added and the resulting organic layer washed with saturated aqueous $NaHCO_3$ (3 x 10 mL). The aqueous phase was freeze dried to give a crude product that was purified by column chromatography (eluent: dichloromethane – methanol 10 - 50% then ethanol – water 0 - 40%) and purified by reversed phase flash chromatography (column: C18, eluent: water – methanol 0 - 100% and 0.1 % TFA) to give the product as purple solid (0.45 g, 70%, containing 23 mol% TBAF). Mp: 193 – 194 °C; ^{1}H NMR (400 MHz, MeOD-d$_4$) δ_H 7.62 (1H, t, *J* 8.8, 16-H), 7.27 (2H, d, *J* 9.2, 7-H), 7.10 (2H, dd, *J* 9.6, 2.4, 6-H), 6.98 (2H, d, *J* 2.0, 4-H), 6.91 (2H, d, *J* 8.4, 15-H), 3.77 (4H, t, *J* 7.2, 9-H), 3.70 (6H, s, 17-H), 3.66 (4H, t, *J* 5.6, 11-H), 3.30 (6H, s, 8-H), 1.92 (4H, quint, *J* 6.0, 10-H); ^{13}C NMR (101 MHz, MeOD-d$_4$) δ_c 157.9 (3-C), 157.7 (14-C), 157.1 (5-C), 154.7 (1-C), 132.3 (16-C), 131.2 (7-C), 114.1 (2-C), 114.0 (6-C), 108.6 (13-C), 104.0 (15-C), 95.9 (4-C), 58.3 (11-C), 55.1 (17-C), 49.5 (9-C), 38.2 (8-C), 29.6 (10-C); ν_{max}/cm^{-1} 3372 (O-H), 1099 (C-O); HRMS: Found $(M)^+$ 491.2380. Calcd. for $C_{29}H_{35}N_2O_5$: $(M)^+$ 491.2540.

(*E*)-N-(9-(2,6-dimethoxyphenyl)-6-(methyl(3-((methylsulfonyl)oxy)propyl)amino)-3H-xanthen-3-ylidene)-N-methyl-3-((methylsulfonyl)oxy)propan-1-aminium iodide **16**

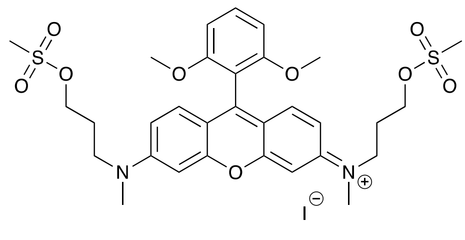

To the solution of compound **15** (0.20 g, 0.38 mmol) in anhydrous dichloromethane (6.0 mL) was added trimethylamine (0.32 mL, 2.28 mmol). After the solution was cooled down to 0 °C, methanesulfonylchloride (0.18 mL, 2.28 mmol) was added dropwise, and the resulting solution stirred at this temperature for 5 hours. After this time, the reaction was quenched with saturated aqueous ammonium chloride solution (10 mL), and the aqueous phase was extracted with dichloromethane (3 x 10 mL). The combined organic layers were washed with aqueous saturated KI solution (10 mL), dried over $MgSO_4$, filtered and concentrated under reduced pressure. The resulting crude product was used in the next step without further purification and characterization.

(*E*)-3-azido-N-(6-((3-azidopropyl)(methyl)amino)-9-(2,6-dimethoxyphenyl)-3H-xanthen-3-ylidene)-*N*-methylpropan-1-aminium iodide **4**

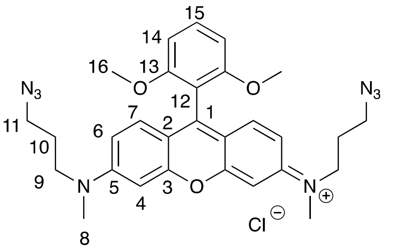

To the solution of crude compound **16** (0.38 mmol) in dry dimethylformamide (6.0 mL) behind a blast shield, was added NaN_3 (0.49 g, 7.60 mmol) using a plastic spatula portion-wise. The resulting mixture was stirred at 40 °C for 16h. After this time, the solvent was removed under reduced pressure and the residue dissolved in dichloromethane (10 ml), which was washed with aqueous saturated KI solution (10 mL), dried over $MgSO_4$, filtered and then removed under reduced pressure. The crude product was purified by column chromatography (eluent: dichloromethane methanol (0 – 5%)), and again by reversed phase flash chromatography (C18, eluent: water – methanol – TFA (0.1%)) to give the product as metallic purple solid (0.15 g, 83%). Mp: 88 – 89 °C; ^{1}H NMR (400 MHz, $CDCl_3$) δ_H 7.54 (1H, t, *J* 8.4, 15-H), 7.26 (2H, d, *J* 9.6, 7-H), 6.92 (2H, dd, *J* 9.6, 2.4, 6-H), 6.88 (2H, d, *J* 2.4, 4-H), 6.75 (2H, d, *J* 8.4, 14-H), 3.71 (4H, t, *J* 7.2, 9-H), 3.66 (6H, s, 16-H), 3.47 (4H, t, *J* 6.0, 11-H), 3.27 (6H, s, 8-H), 1.96 (4H, quint, *J* 6.4, 10-H). ^{13}C NMR (101 MHz, $CDCl_3$) δ_c 158.1 (3-C), 157.6 (13-C), 156.8 (5-C), 155.5 (1-C), 132.6 (15-C), 131.9 (7-C), 114.8 (2-C), 113.9 (6-C), 108.5 (12-C), 104.1 (14-C), 96.7 (4-C), 56.0 (16-C), 50.3 (9-C), 48.6 (11-C), 39.4 (8-C), 26.6 (10-C); ν_{max}/cm^{-1} 2095 (N=N-N), 1099 (C-O); HRMS: Found $(M)^+$ 541.2747. Calcd. for $C_{29}H_{33}N_8O_3$: $(M)^+$ 541.2670. UV/VIS (10.7 μM in water and 1 % DMSO): λ_{max}= 558 nm, A_2= 0.76; Fluorescence (107 nM in water and 0.01% DMSO): λ_{ex}= 557 nm, Intensity$_{ex}$= 127 a.u.; λ_{em}= 580 nm, Intensity$_{em}$= 127 a.u.

NMR Spectra, Mass Spec and IR Data

3,6-dihydroxy-9*H*-xanthen-9-one **6**

1H NMR

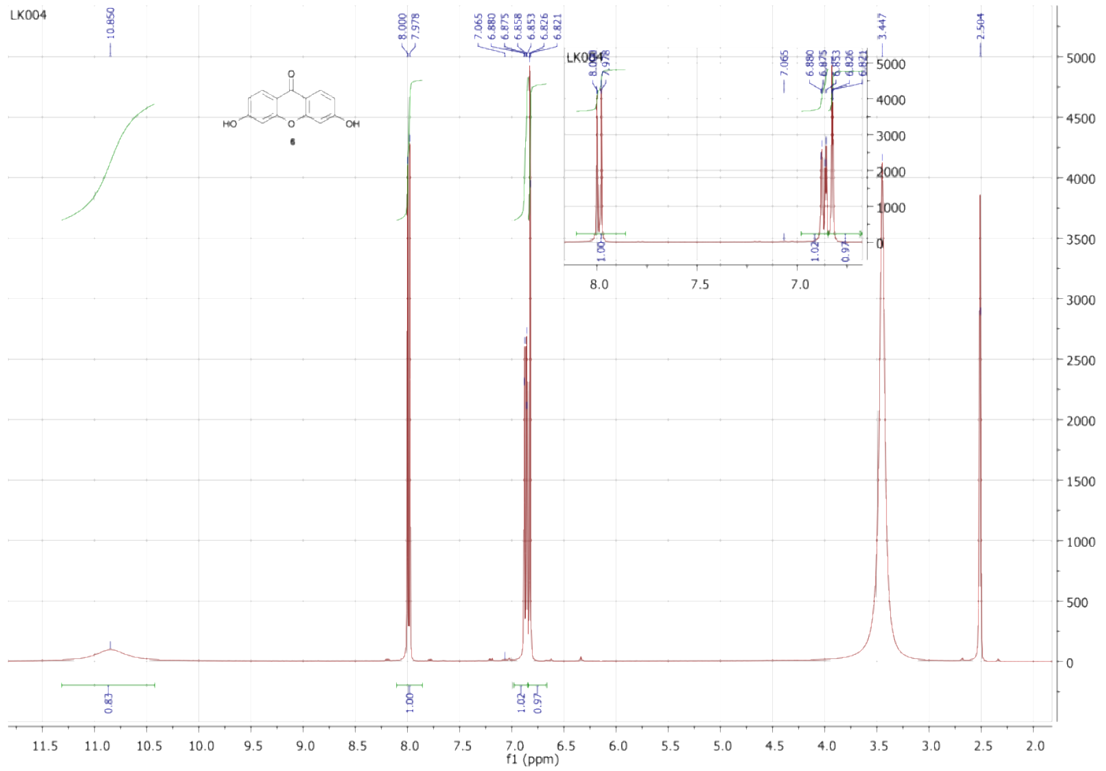

13C NMR

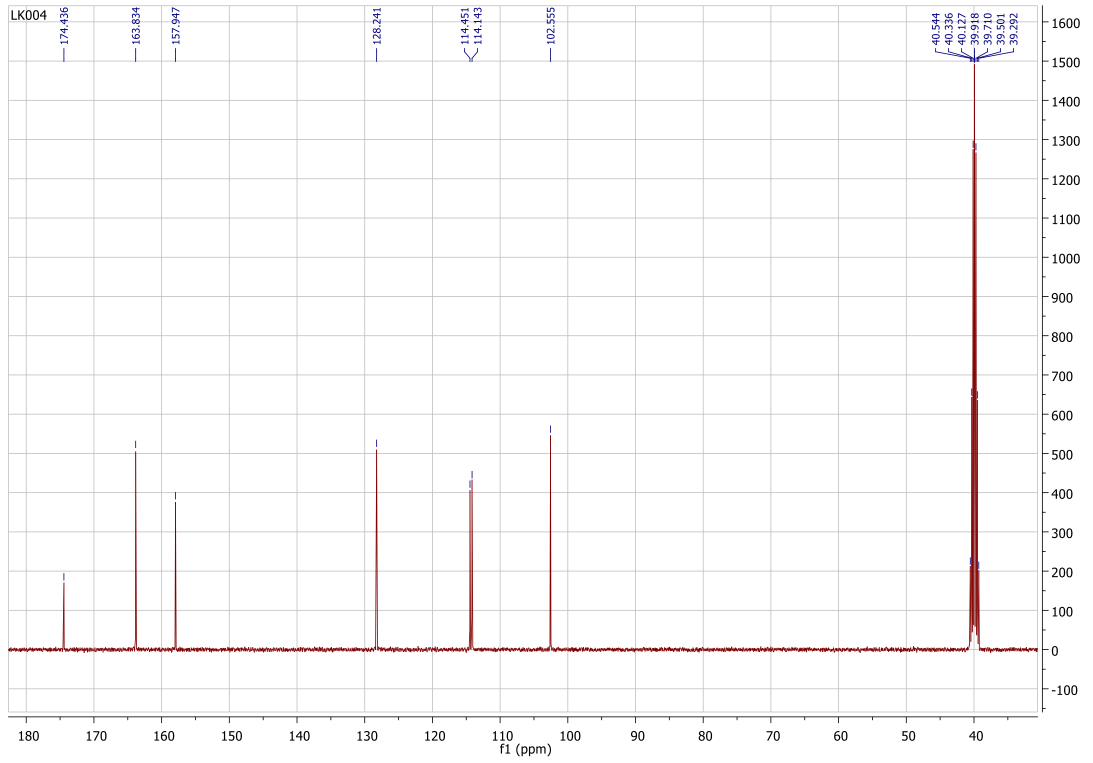

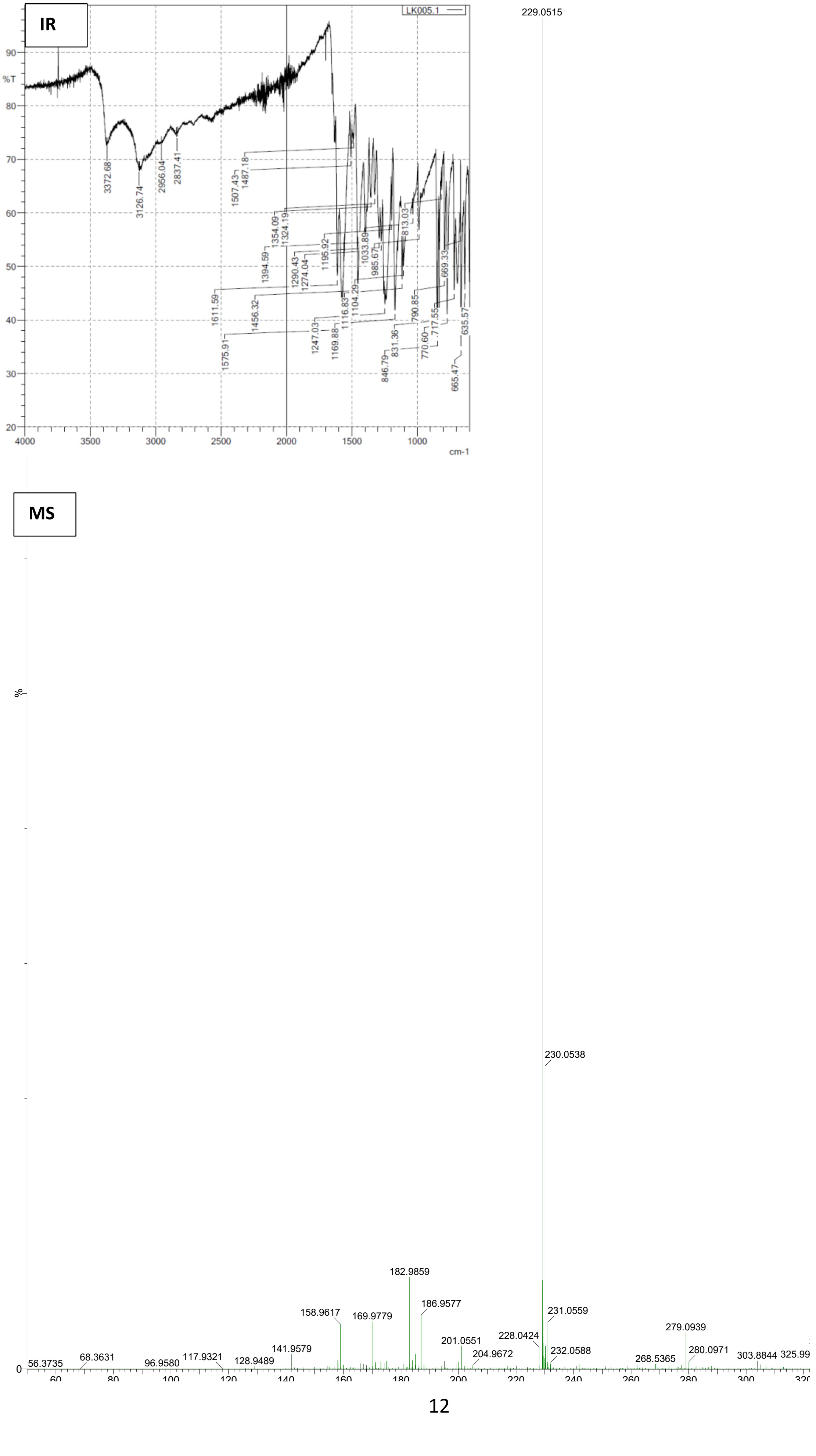
IR
LK005.1
%T
cm-1
3372.68
3126.74
2956.04
2837.41
1611.59
1575.91
1507.43
1487.18
1456.32
1394.59
1354.09
1324.19
1290.43
1274.04
1247.03
1195.92
1169.88
1116.83
1104.29
1033.89
985.67
846.79
831.36
813.03
790.85
770.60
717.55
669.33
665.47
635.57
MS
%
229.0515
230.0538
182.9859
186.9577
169.9779
158.9617
141.9579
231.0559
279.0939
228.0424
201.0551
204.9672
232.0588
280.0971
268.5365
303.8844
325.99
56.3735
68.3631
96.9580
117.9321
128.9489

9-oxo-9*H*-xanthene-3,6-diyl bis(trifluoromethanesulfonate) **7**

1H NMR

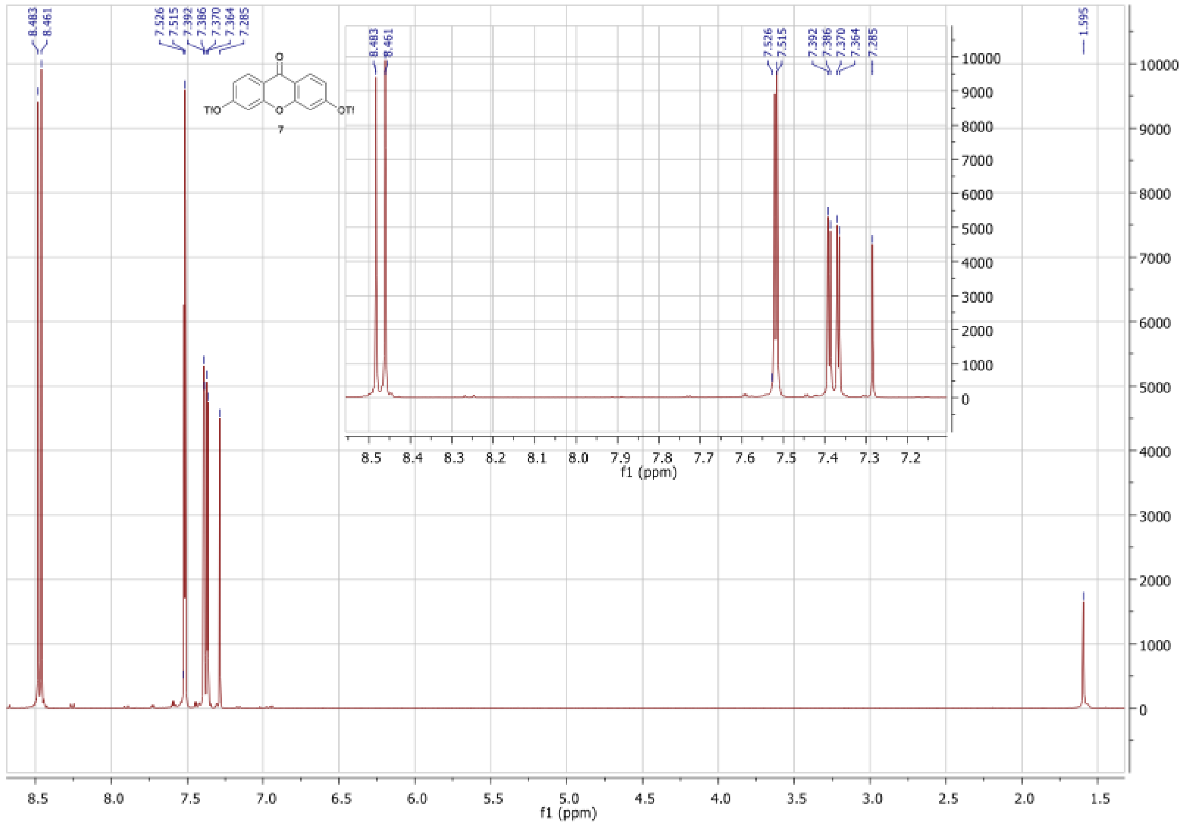

13C NMR

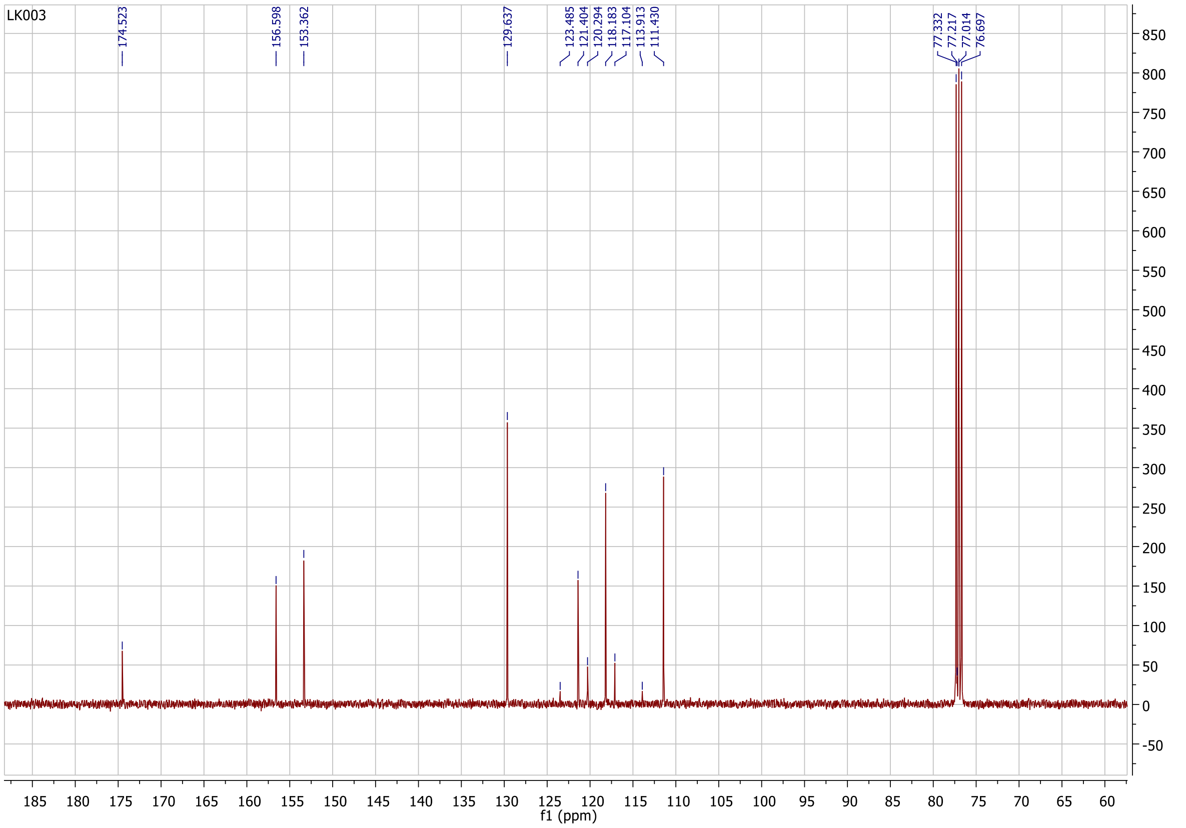

19F NMR

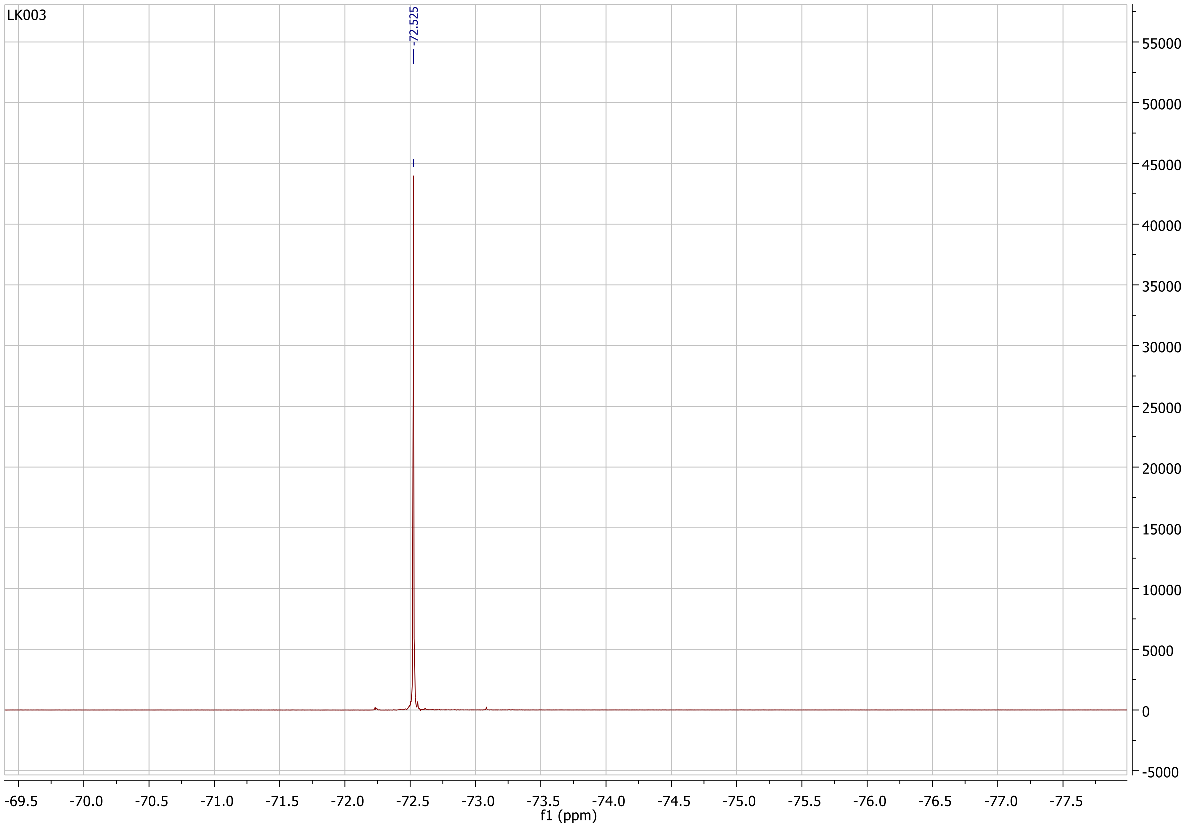
LK003
-72.525
f1 (ppm)

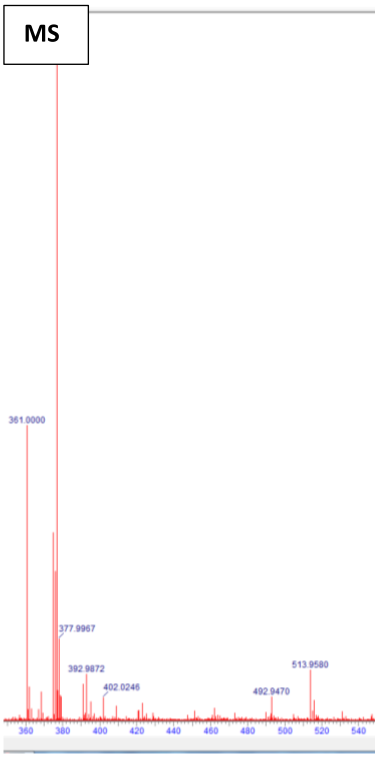
MS
361.0000
377.9967
392.9872
402.0246
492.9470
513.9580

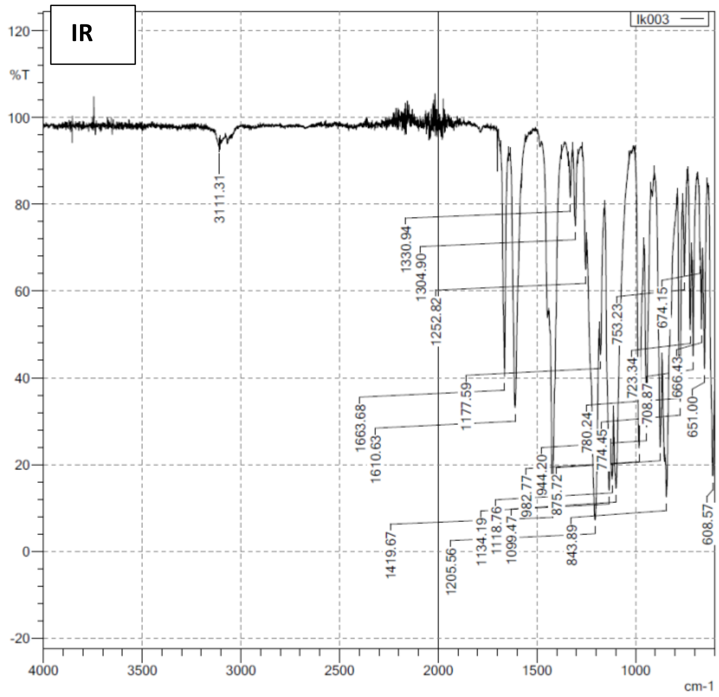
IR
lk003
%T
3111.31
1330.94
1304.90
1252.82
1663.68
1610.63
1177.59
1419.67
1205.56
1134.19
1118.76
1099.47
982.77
944.20
875.72
843.89
780.24
774.45
753.23
723.34
708.87
674.15
666.43
651.00
608.57
cm-1

Di-tert-butyl 4,4'-(9-oxo-9H-xanthene-3,6-diyl)bis(piperazine-1-carboxylate) **8**

1H NMR

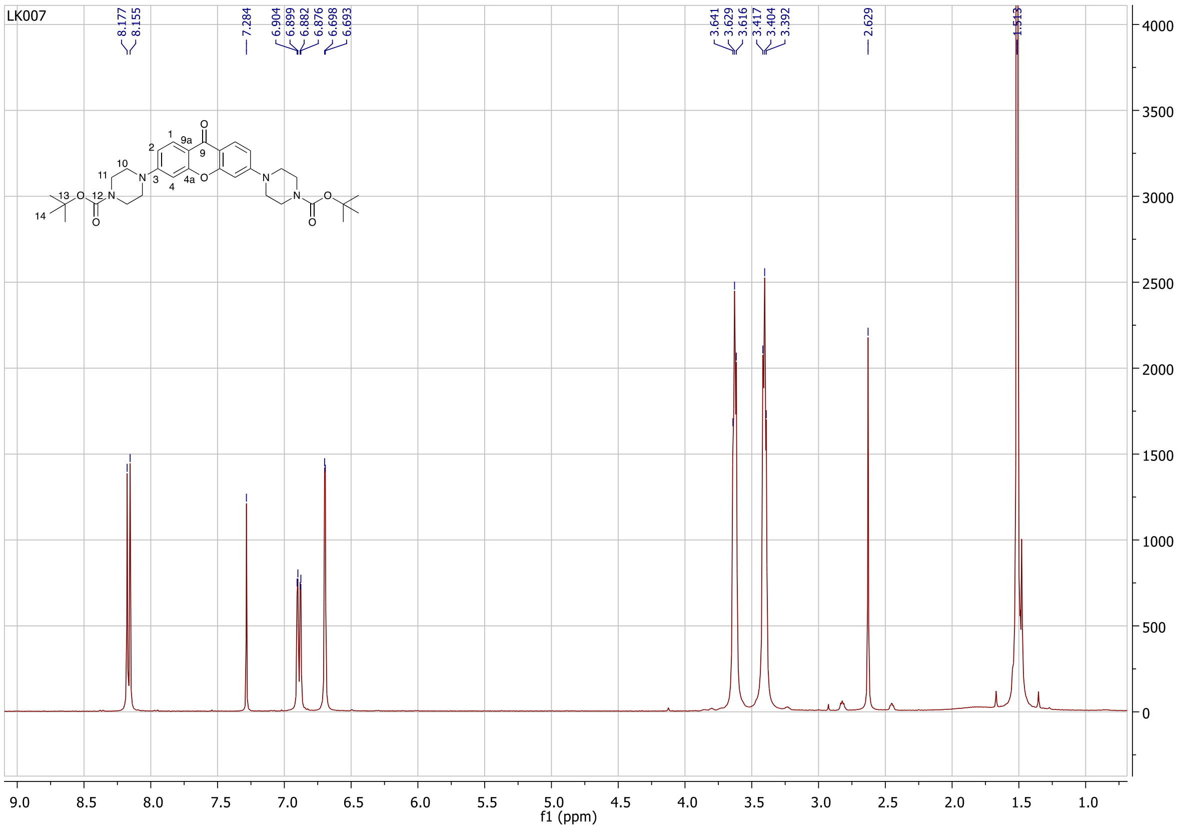

13C NMR

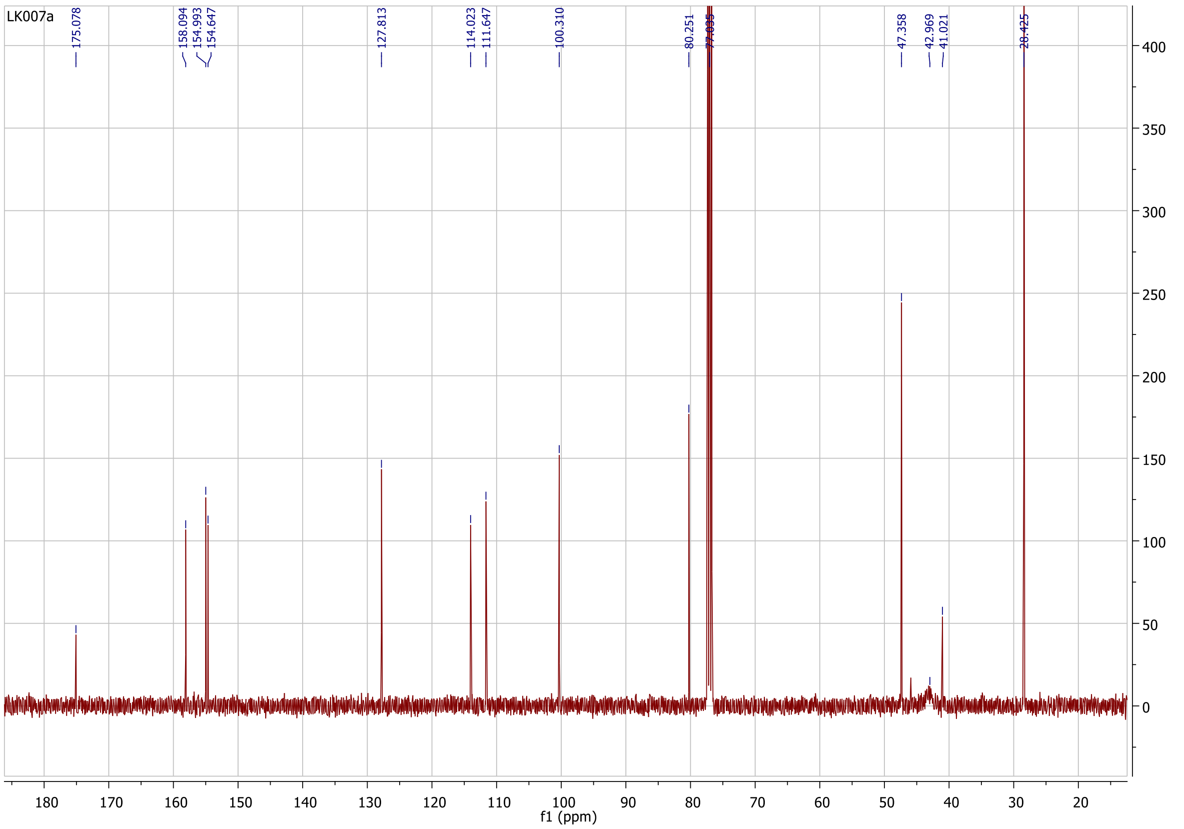

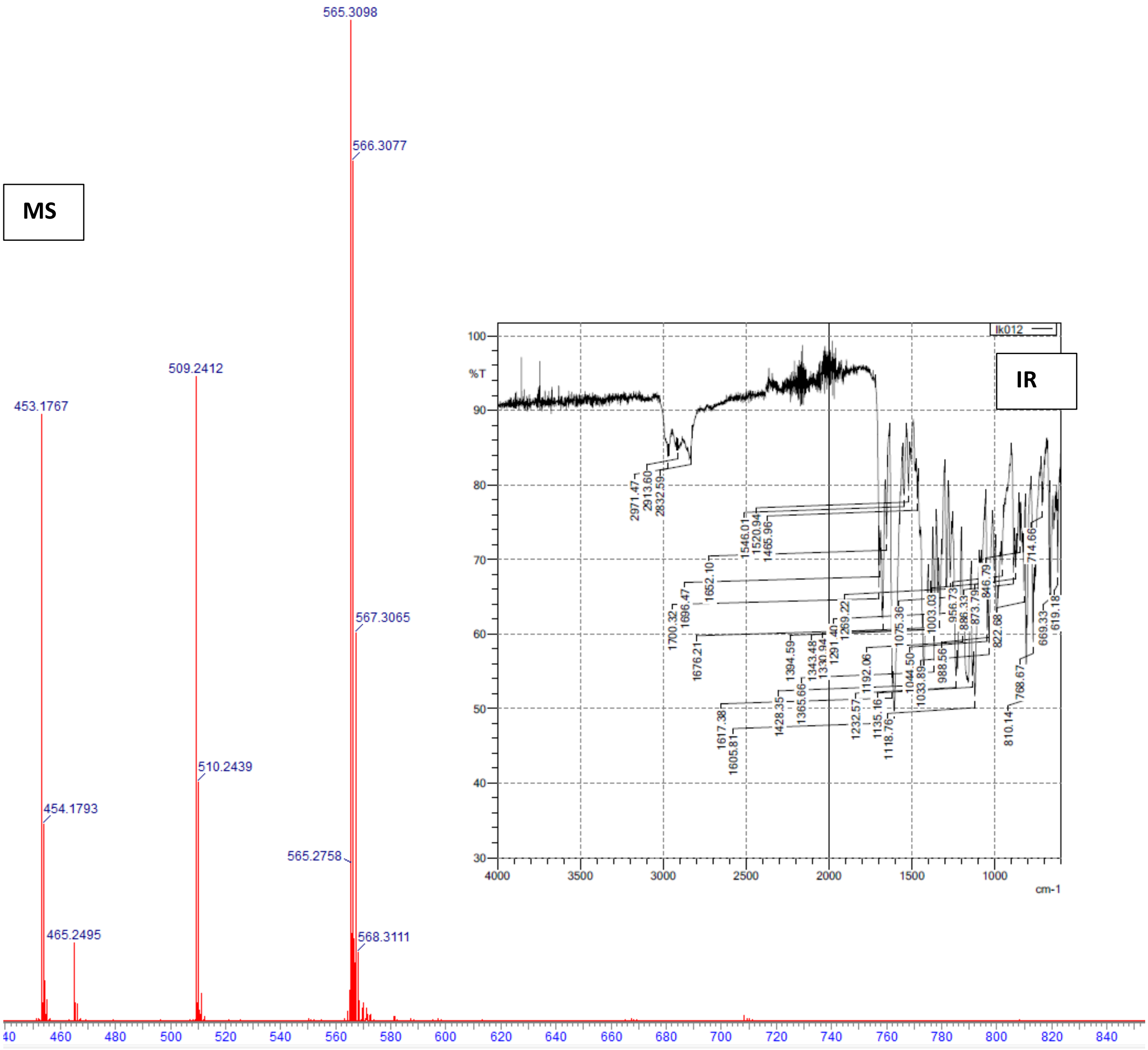
MS
565.3098
566.3077
567.3065
568.3111
565.2758
509.2412
510.2439
453.1767
454.1793
465.2495
IR
lk012
%T
cm-1
2971.47
2913.60
2832.59
1546.01
1520.94
1465.96
1652.10
1696.47
1700.32
1676.21
1617.38
1605.81
1428.35
1394.59
1365.66
1343.48
1330.94
1291.40
1269.22
1232.57
1192.06
1135.16
1118.76
1075.36
1044.50
1033.89
1003.03
988.56
956.73
886.33
873.79
846.79
822.68
810.14
768.67
714.66
669.33
619.18

4-(tert-butoxycarbonyl)-1-(6-(4-(tert-butoxycarbonyl)piperazin-1-yl)-9-(2,6-dimethoxyphenyl)-3H-xanthen-3-ylidene)piperazin-1-ium chloride **9**

1H NMR

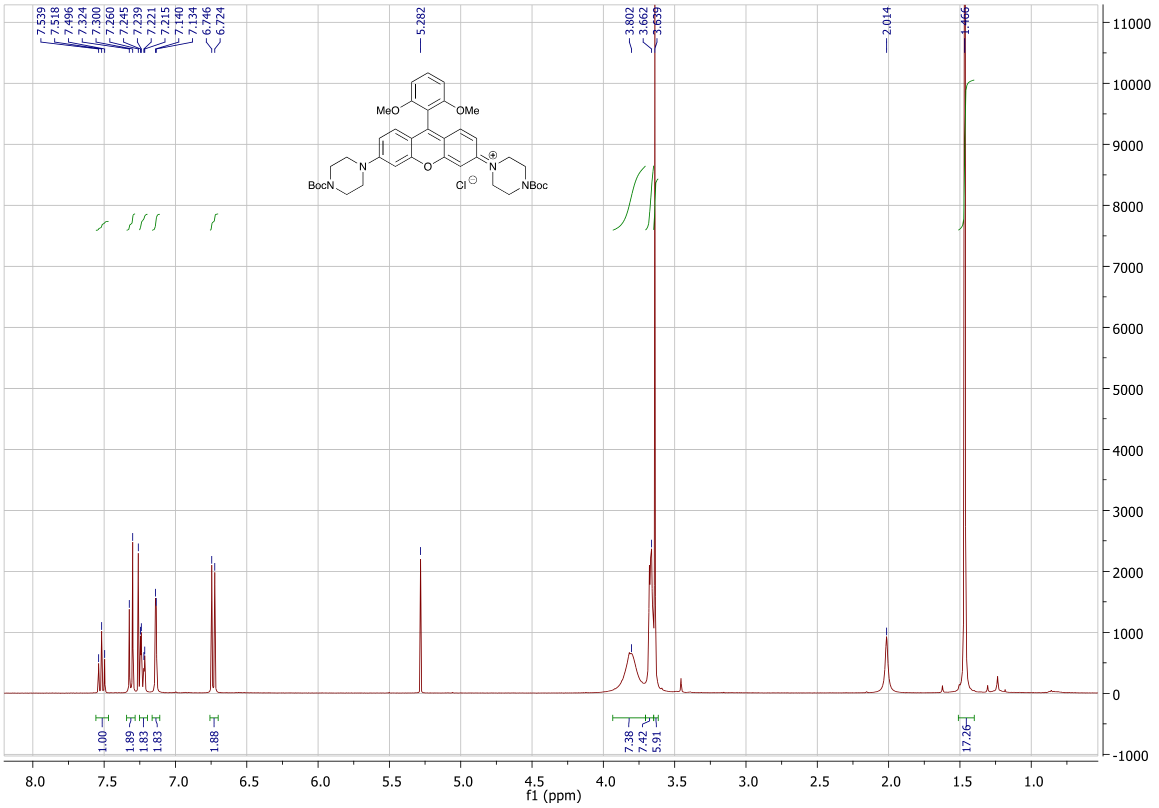

13C NMR

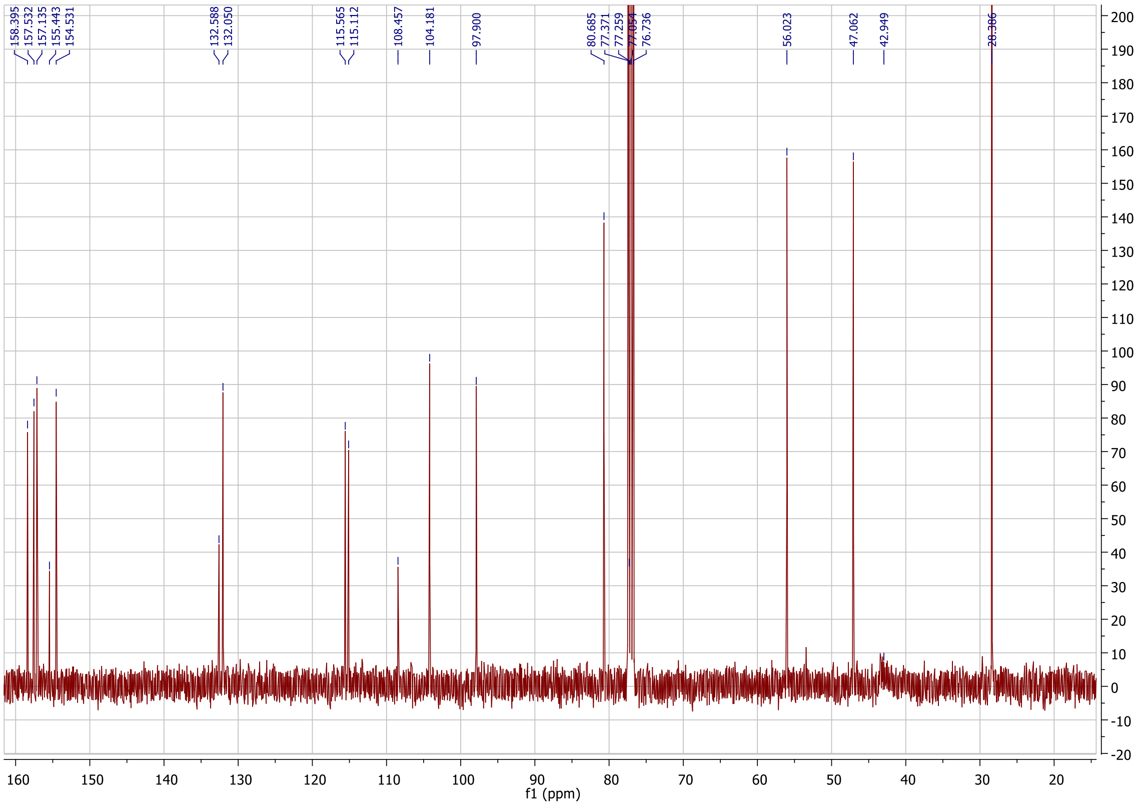

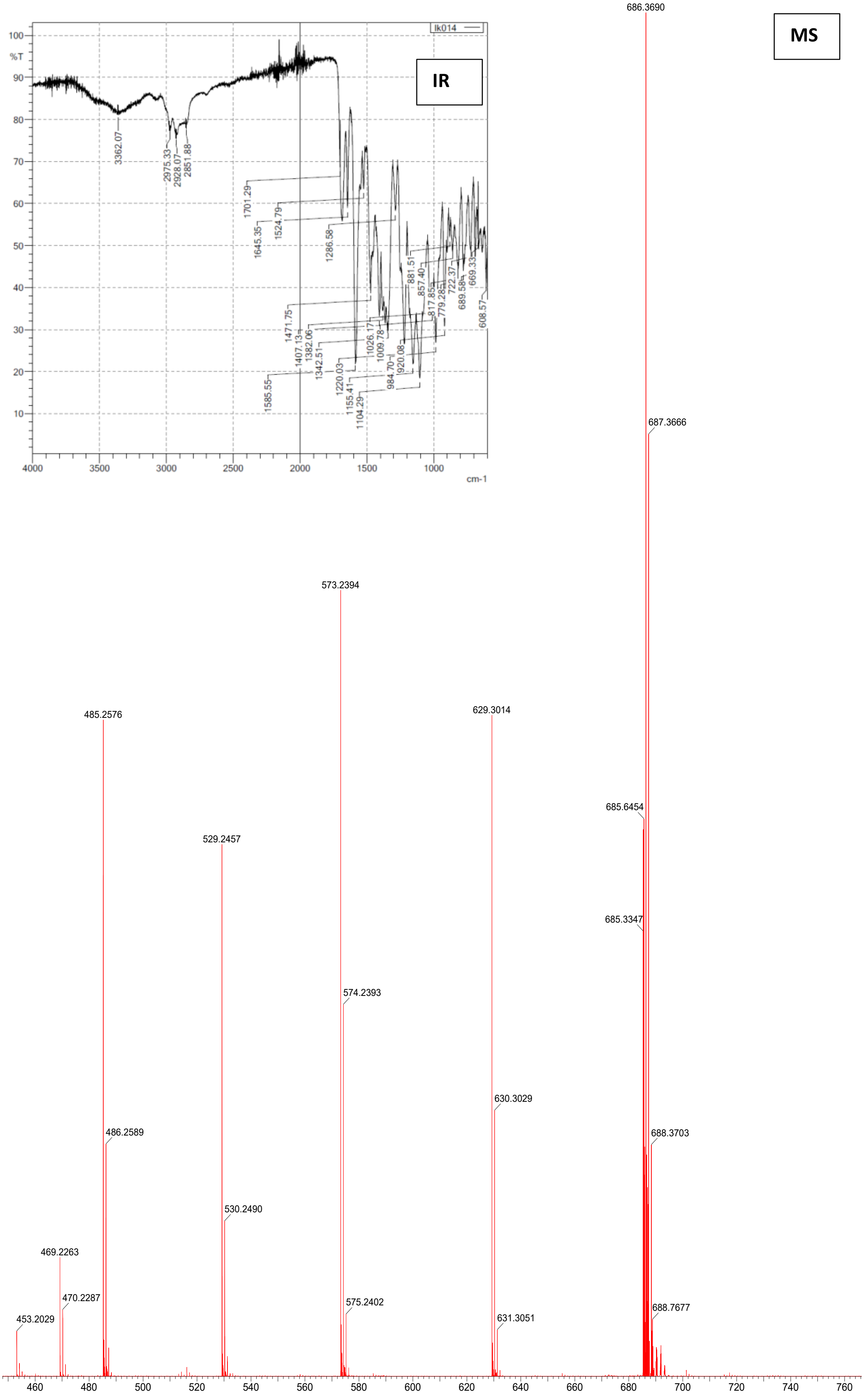
IR
lk014
%T
cm-1
3362.07
2975.33
2928.07
2851.88
1701.29
1645.35
1524.79
1286.58
1471.75
1407.13
1382.06
1342.51
1585.55
1220.03
1155.41
1104.29
1026.17
1009.78
984.70
920.08
881.51
857.40
817.85
779.28
722.37
689.58
669.33
608.57
MS
686.3690
687.3666
573.2394
485.2576
629.3014
685.6454
529.2457
685.3347
574.2393
630.3029
486.2589
688.3703
530.2490
469.2263
470.2287
575.2402
688.7677
453.2029
631.3051

1-(9-(2,6-dimethoxyphenyl)-6-(piperazin-1-ium-1-yl)-3H-xanthen-3-ylidene)piperazine-1,4-diium chloride **10**

1H NMR

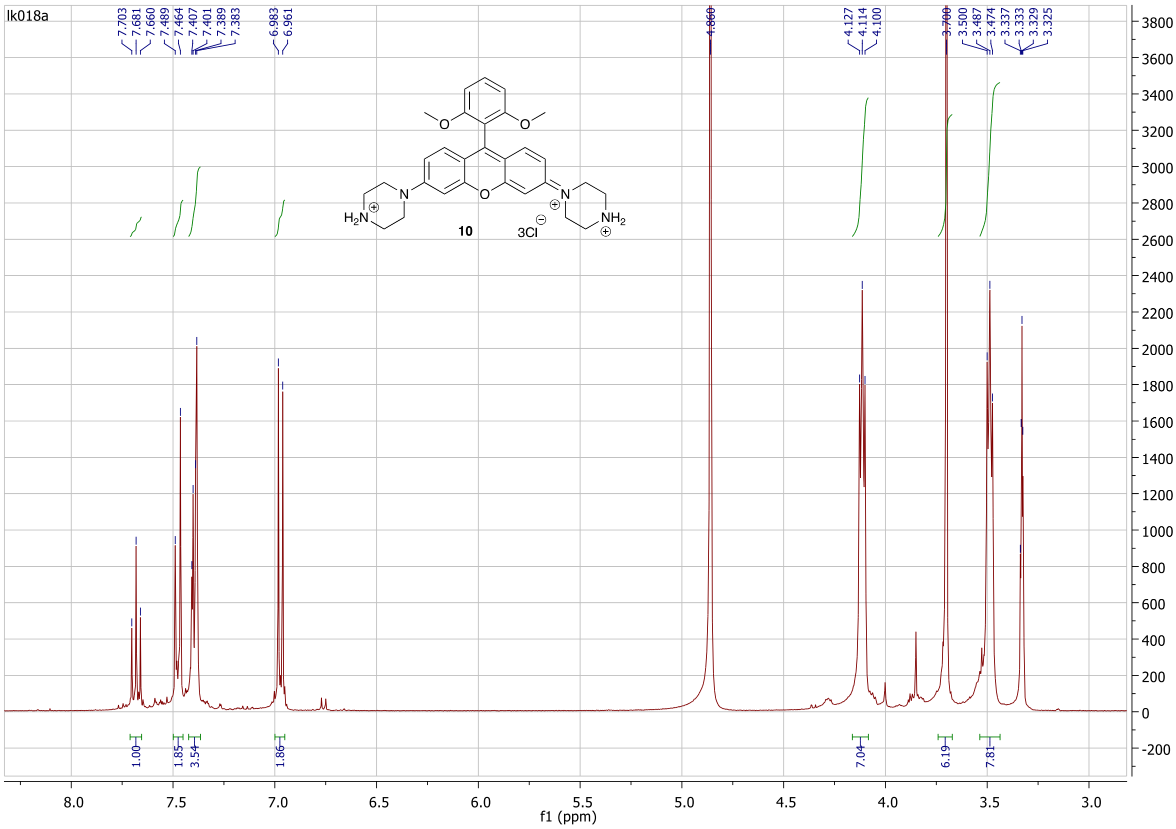

13C NMR

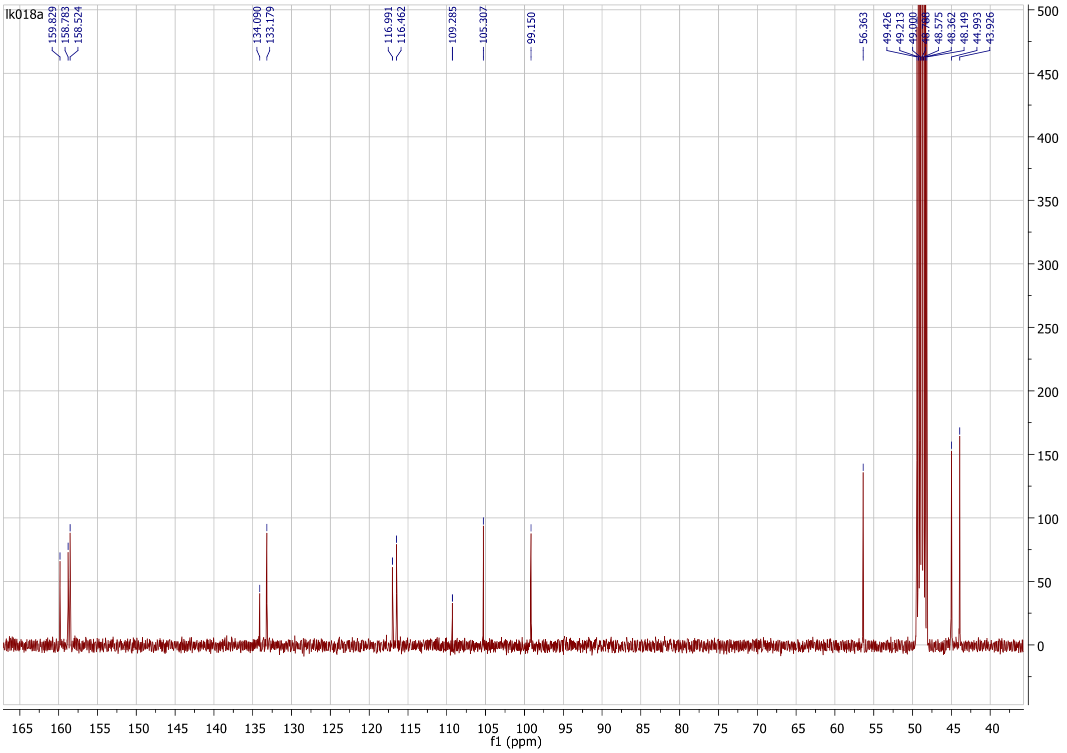

MS

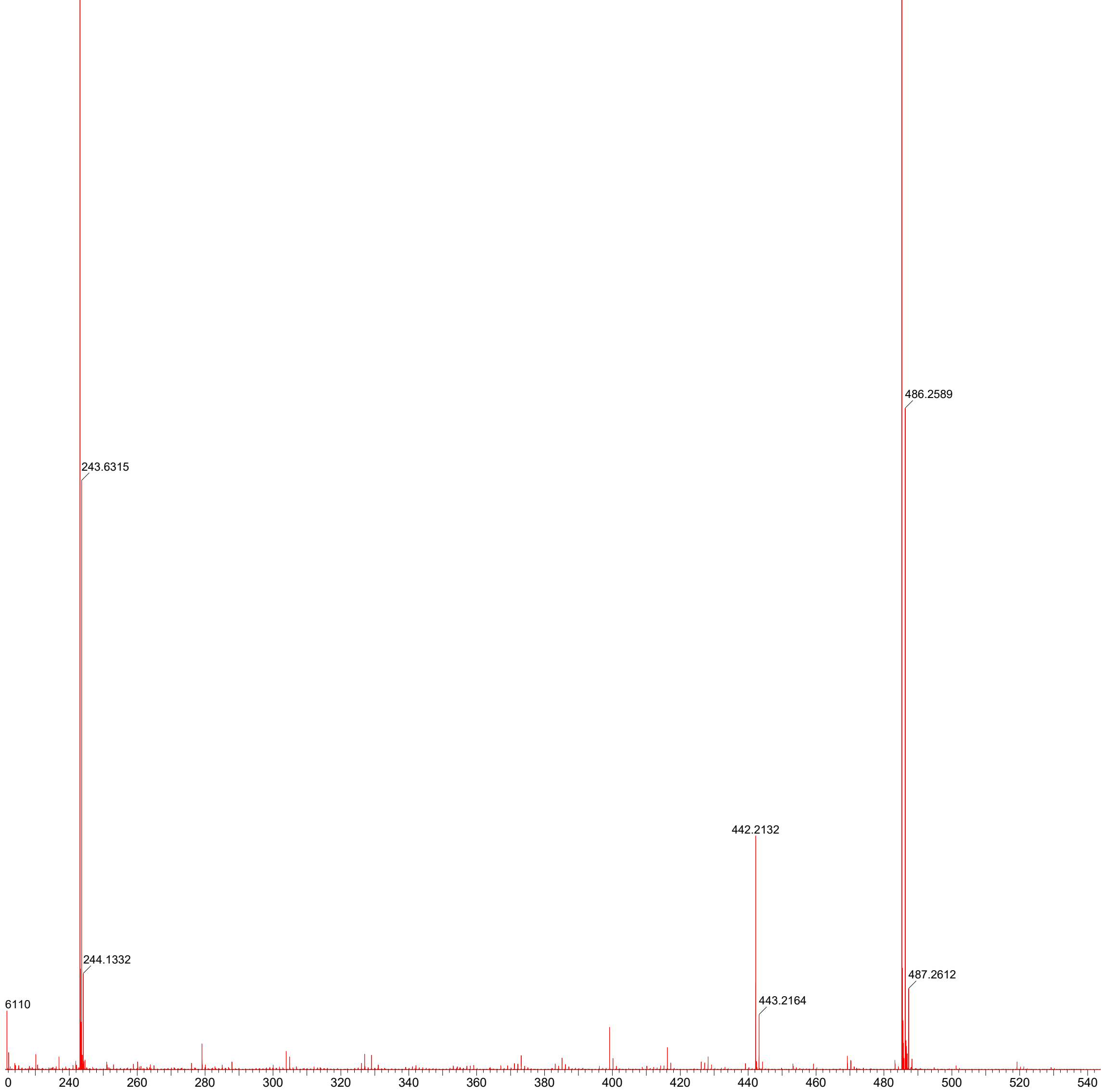
243.1334
243.6315
244.1332
6110
442.2132
443.2164
485.2576
486.2589
487.2612
0
240
260
280
300
320
340
360
380
400
420
440
460
480
500
520
540

IR

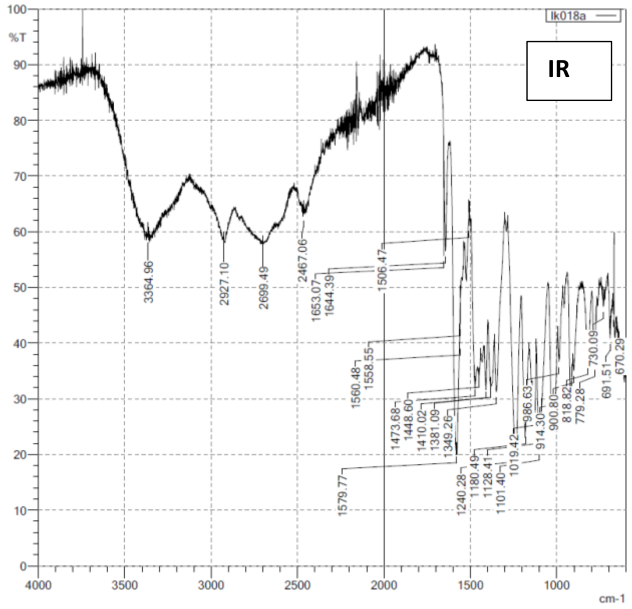
lk018a
%T
100
90
80
70
60
50
40
30
20
10
0
4000
3500
3000
2500
2000
1500
1000
cm-1
3364.96
2927.10
2699.49
2467.06
1653.07
1644.39
1506.47
1560.48
1558.55
1579.77
1473.68
1448.60
1410.02
1381.09
1349.26
1240.28
1180.49
1128.41
1101.40
1019.42
986.63
914.30
900.80
818.82
779.28
730.09
691.51
670.29

4-(2-chloroacetyl)-1-(6-(4-(2-bromo/chloroacetyl)piperazin-1-yl)-9-(2,6-dimethoxyphenyl)-3H-xanthen-3-ylidene)piperazin-1-ium chloride **11**

1H NMR

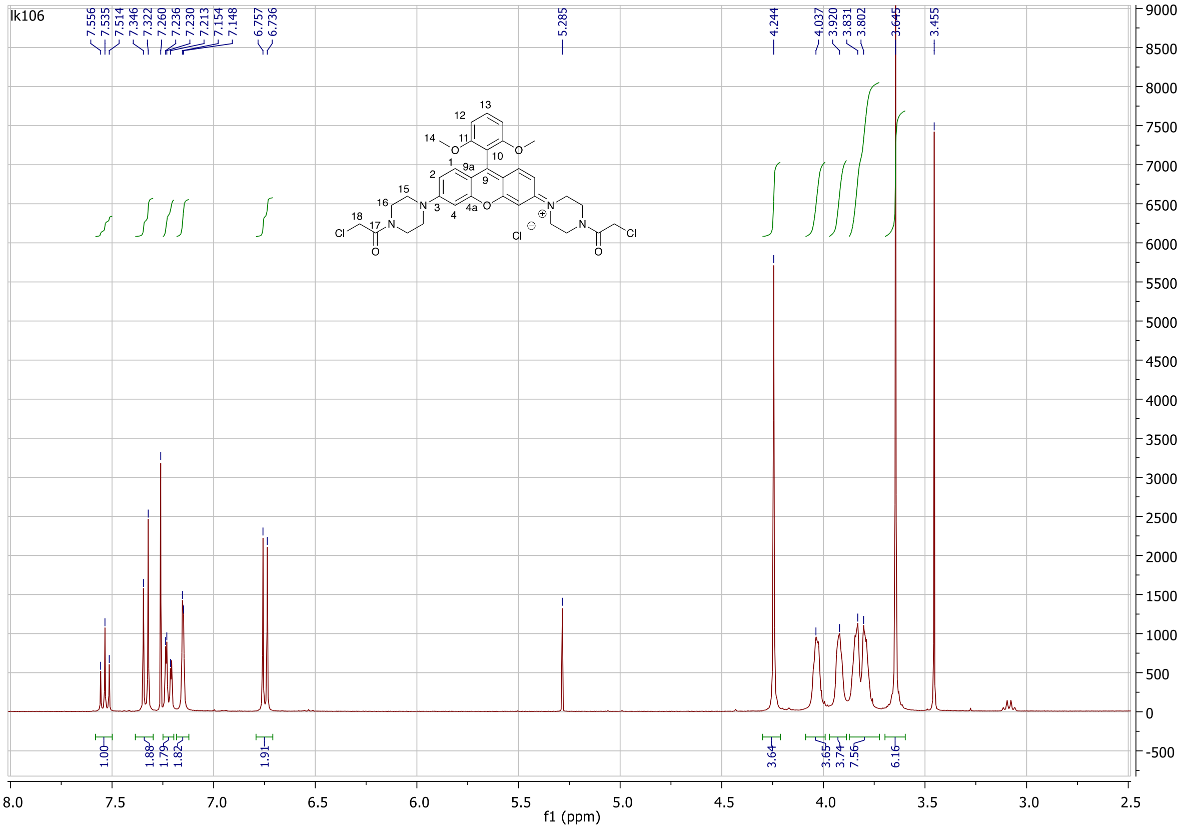

13C NMR

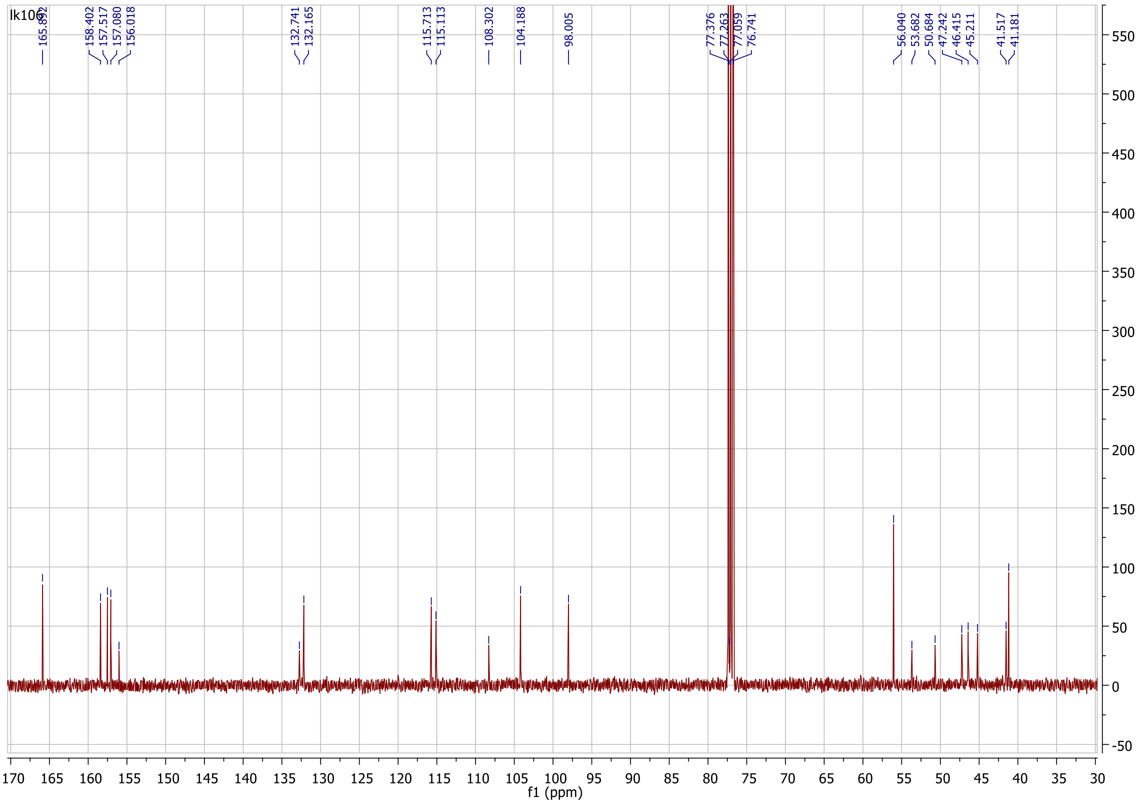

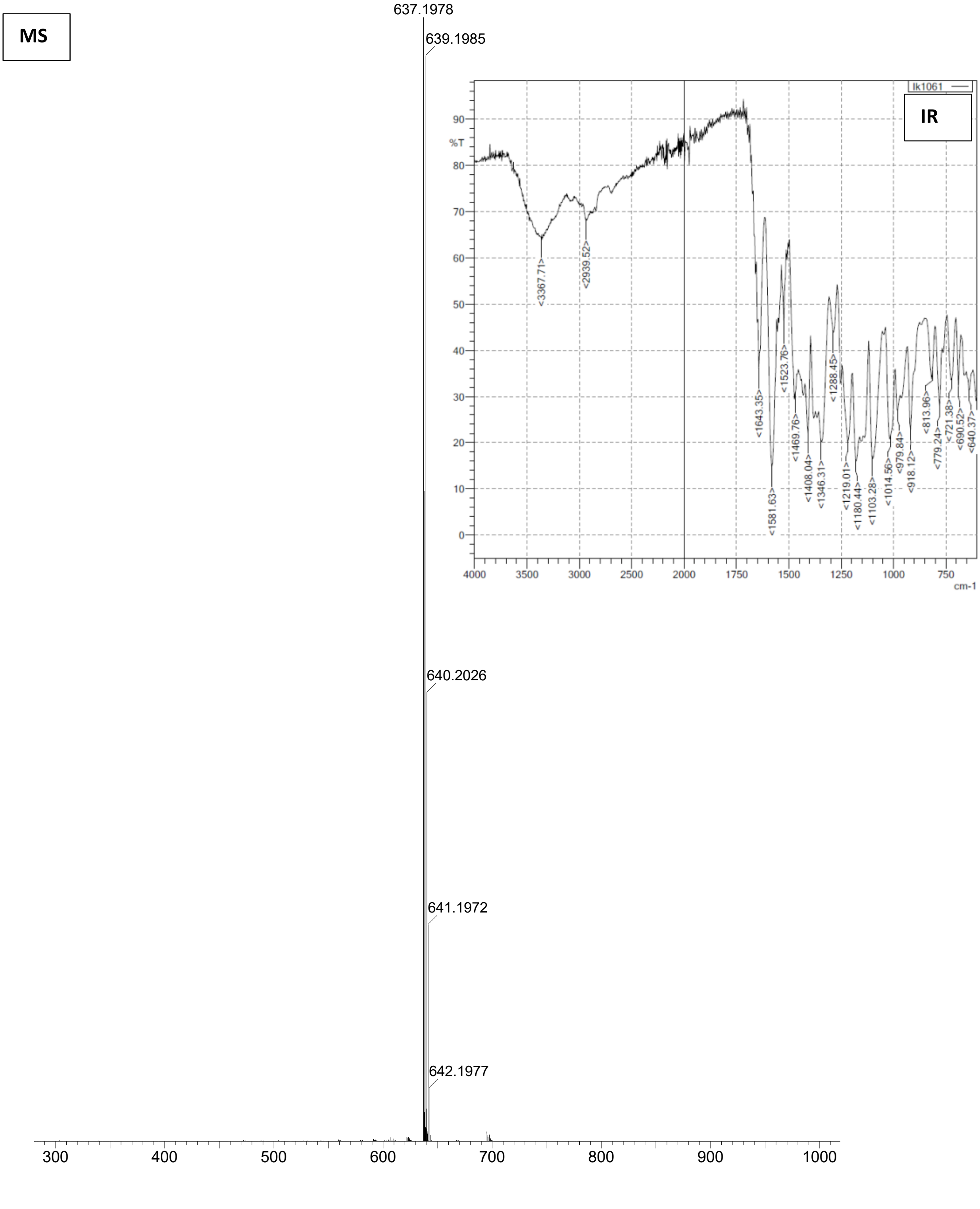
MS
637.1978
639.1985
640.2026
641.1972
642.1977
300
400
500
600
700
800
900
1000
IR
lk1061
%T
90
80
70
60
50
40
30
20
10
0
4000
3500
3000
2500
2000
1750
1500
1250
1000
750
cm-1
<3367.71>
<2939.52>
<1643.35>
<1581.63>
<1523.76>
<1469.76>
<1408.04>
<1346.31>
<1288.45>
<1219.01>
<1180.44>
<1103.28>
<1014.56>
<979.84>
<918.12>
<813.96>
<779.24>
<721.38>
<690.52>
<640.37>

1-(9-(2,6-dimethoxyphenyl)-6-(4-(2-iodoacetyl)piperazin-1-yl)-3H-xanthen-3-ylidene)-4-(2-iodoacetyl)piperazin-1-ium iodide **2**

1H NMR

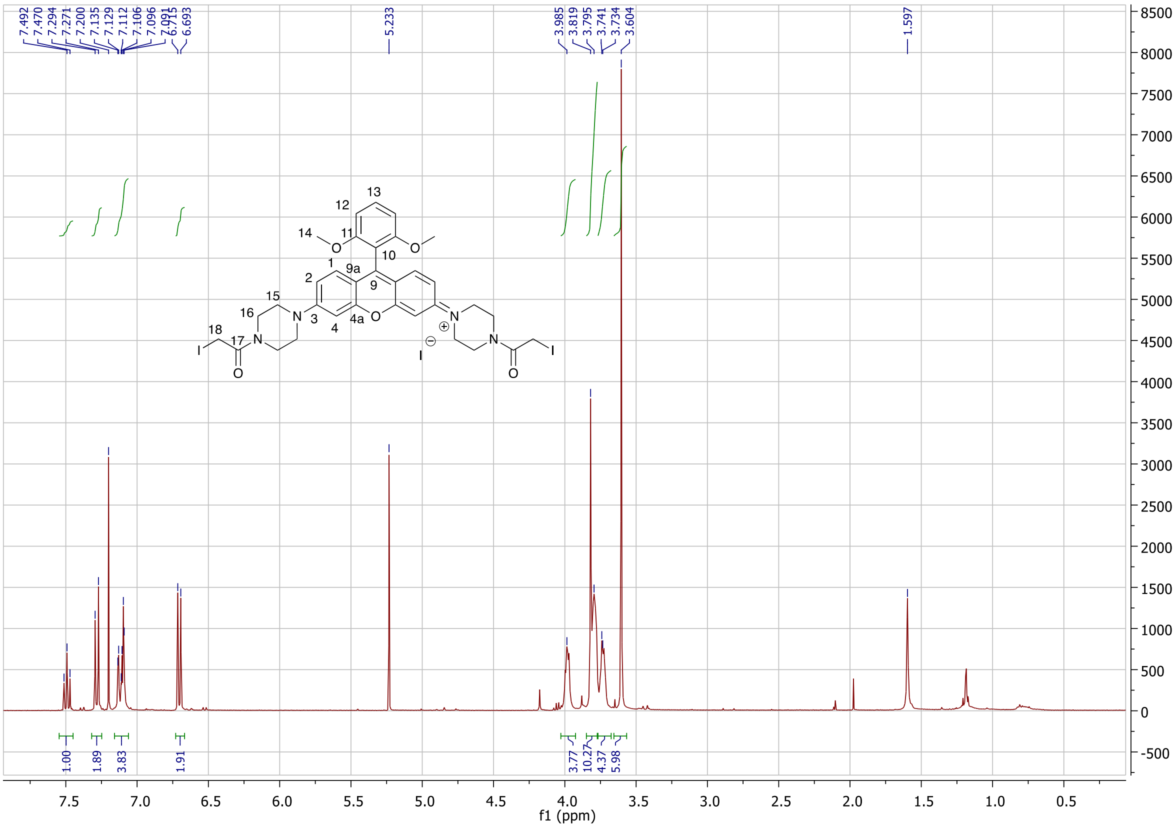

13C NMR

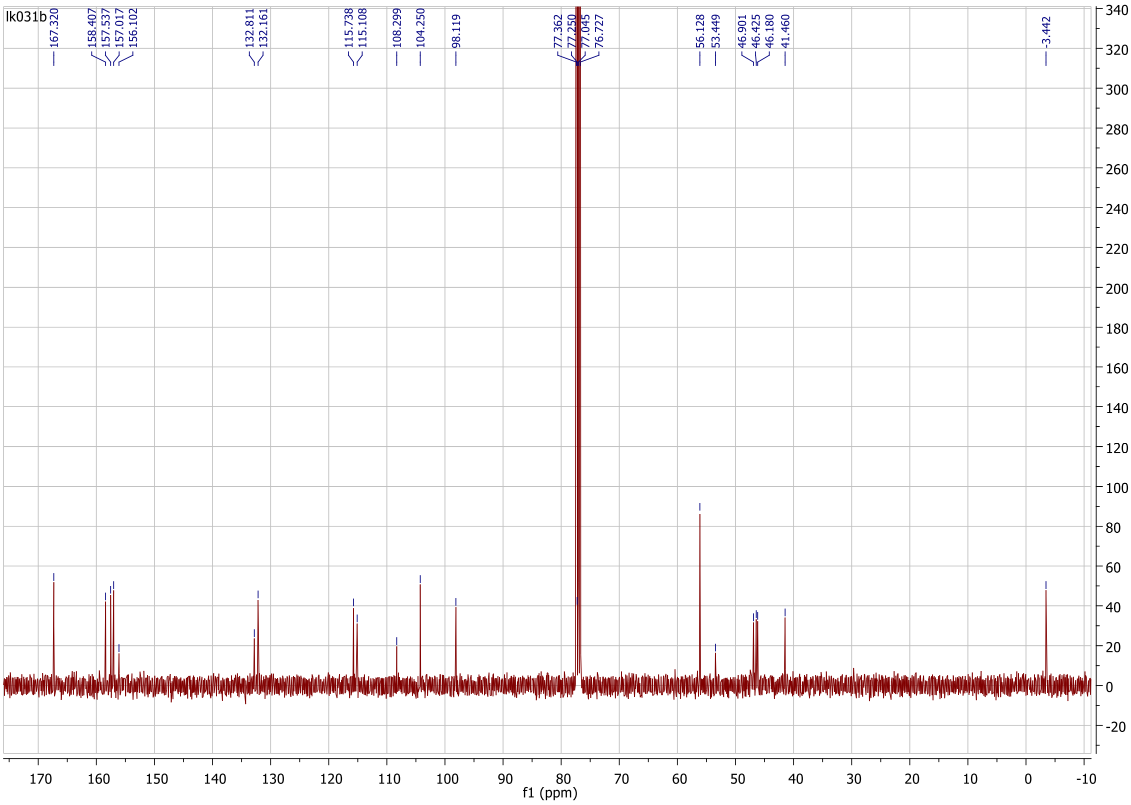

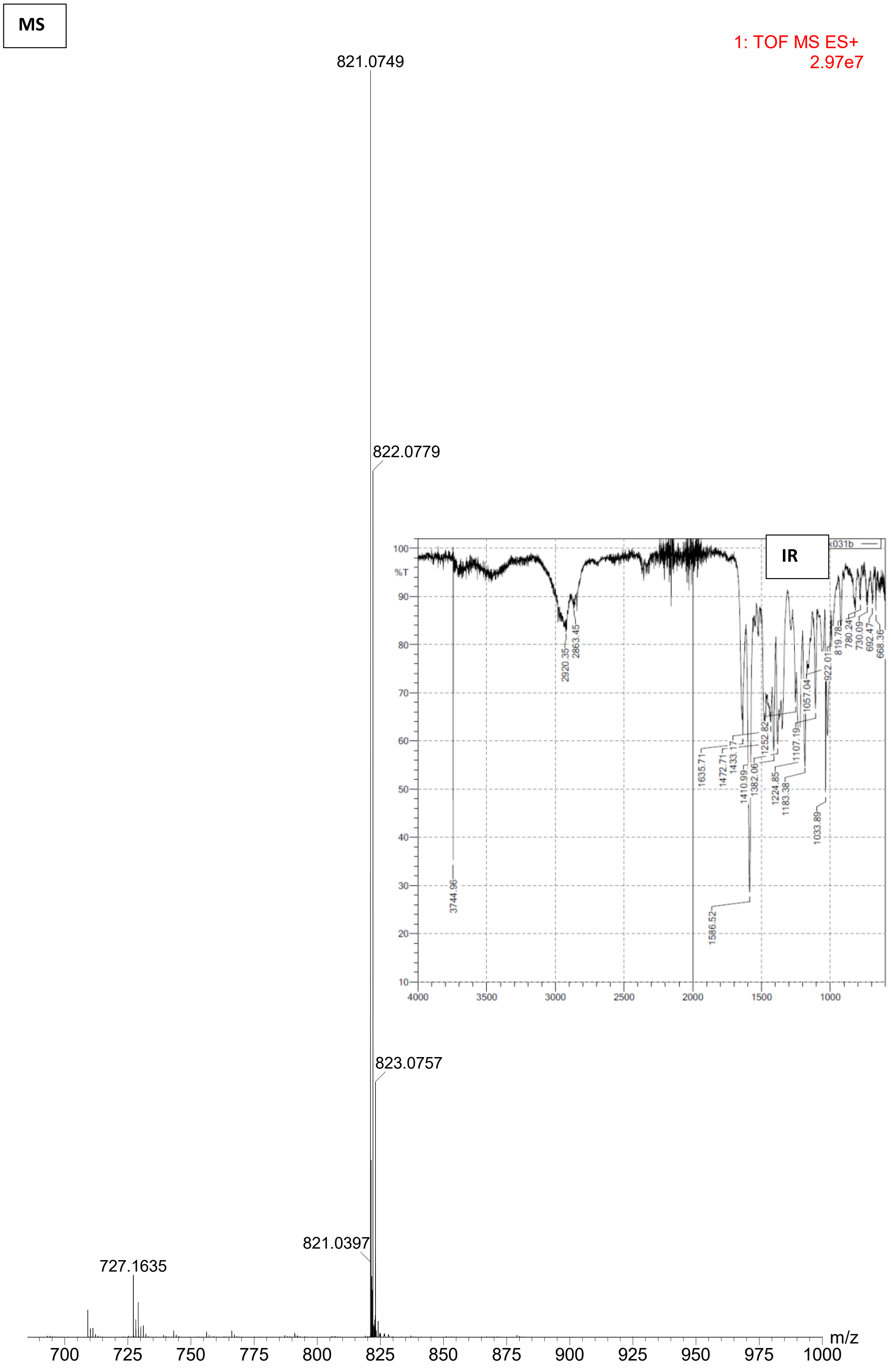
MS
1: TOF MS ES+
2.97e7
821.0749
822.0779
823.0757
821.0397
727.1635
m/z
700
725
750
775
800
825
850
875
900
925
950
975
1000
IR
%T
3744.96
2920.35
2863.45
1635.71
1586.52
1472.71
1433.17
1410.99
1382.06
1252.82
1224.85
1183.38
1107.19
1057.04
1033.89
922.01
819.78
780.24
730.09
692.47
668.36
4000
3500
3000
2500
2000
1500
1000

Spectral Data **2**

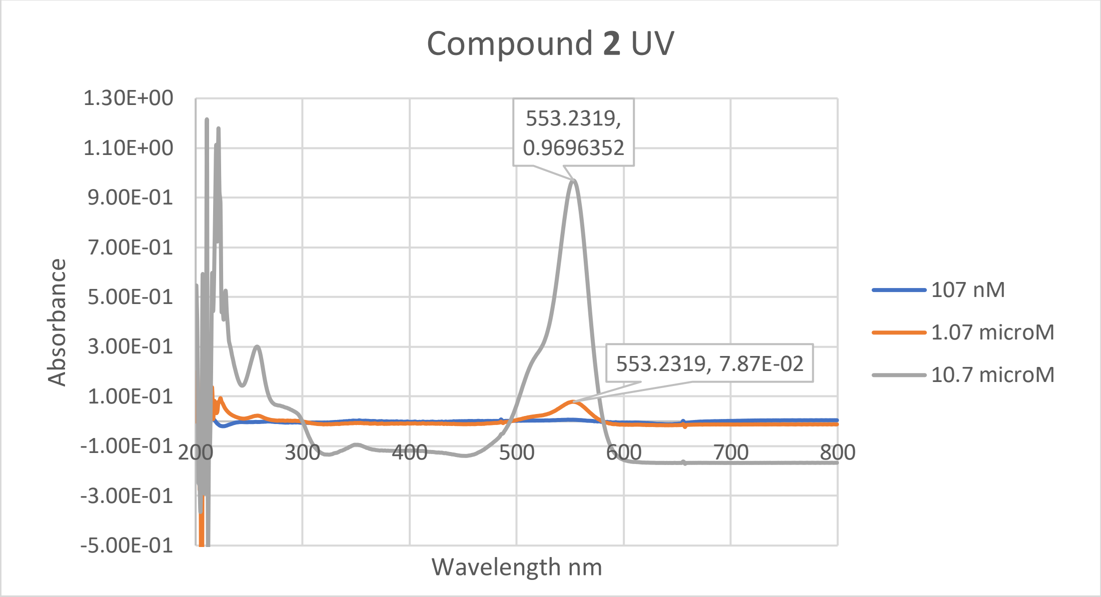

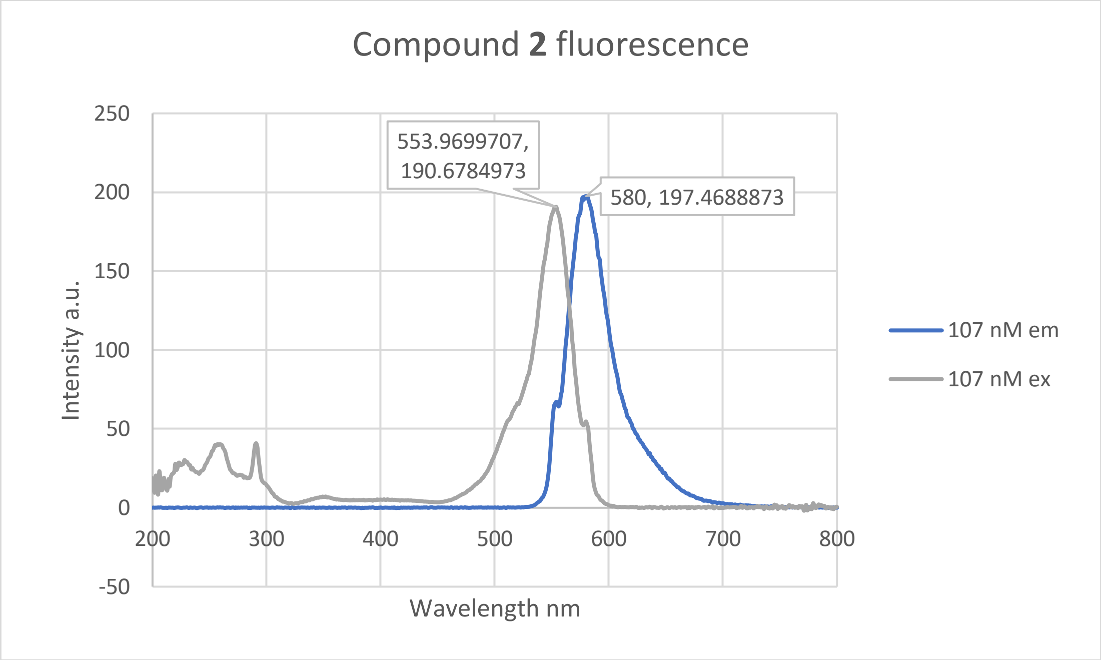

1-(9-(2,6-dimethoxyphenyl)-6-(4-(prop-2-yn-1-yl)piperazin-1-yl)-3H-xanthen-3-ylidene)-4-(prop-2-yn-1-yl)piperazin-1-ium chloride **3**

1H NMR

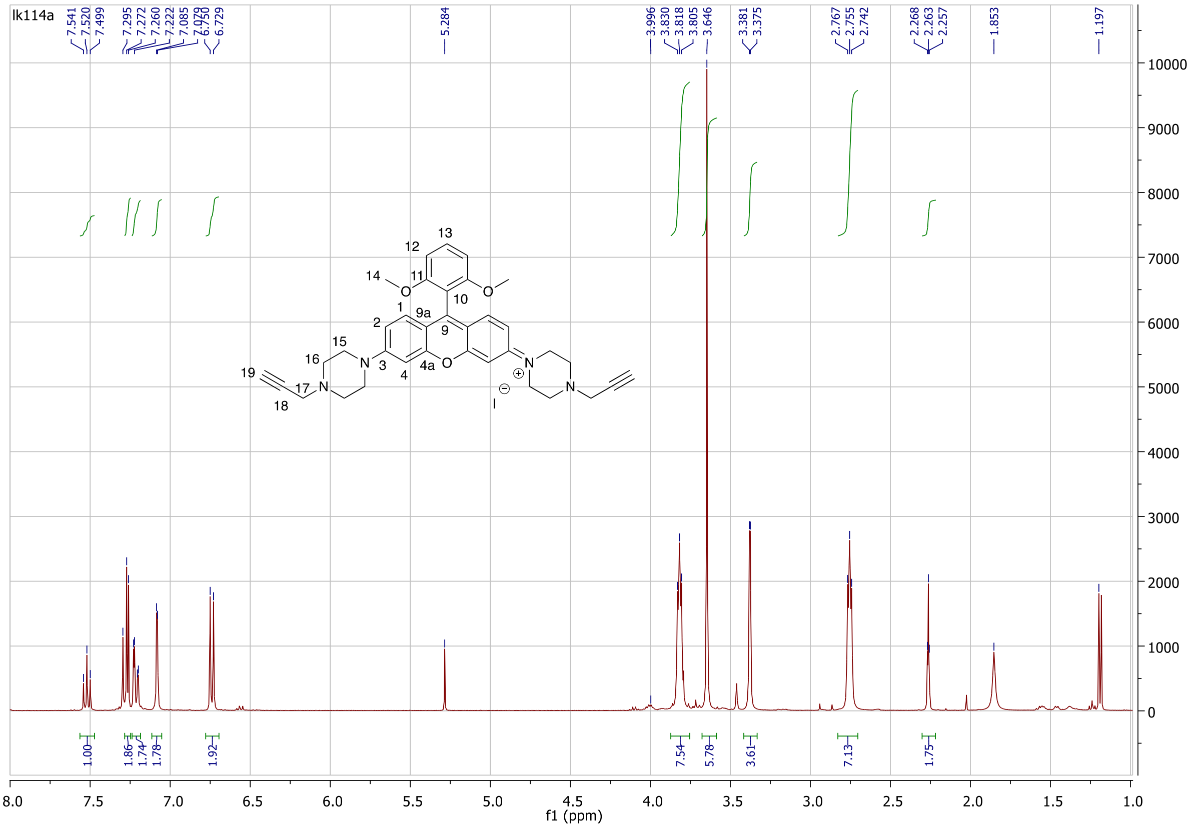

13C NMR

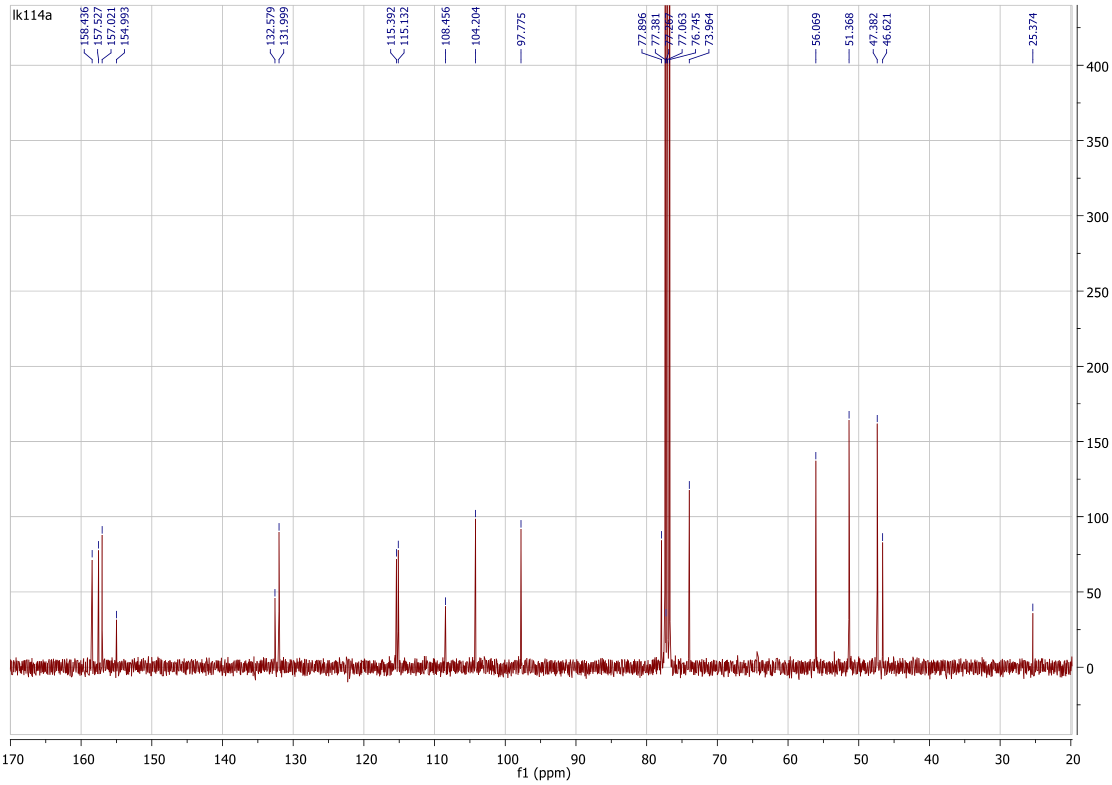

ıol

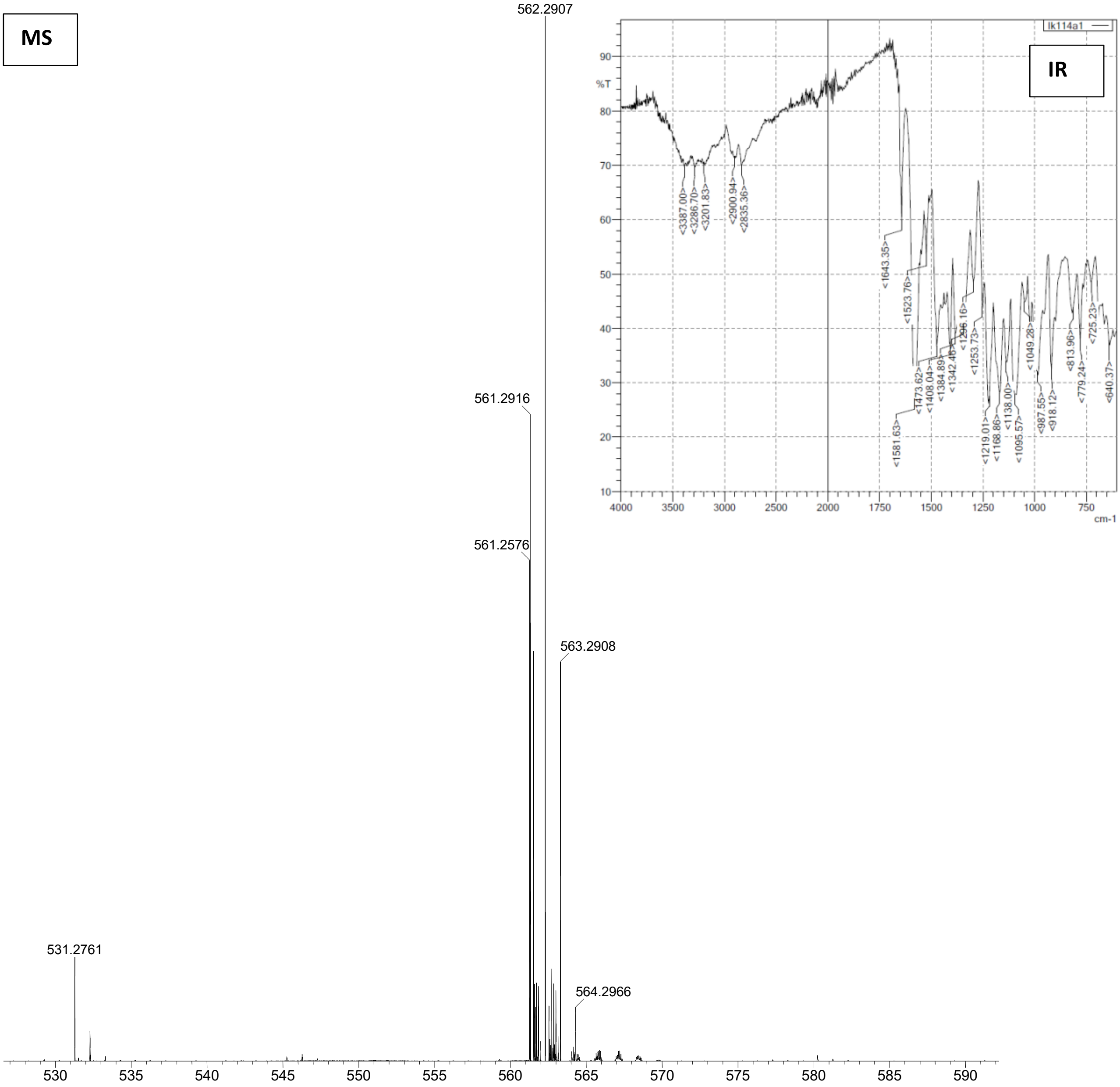

3-((tert-butyldimethylsilyl)oxy)-N-methylpropan-1-amine **12**

1H NMR

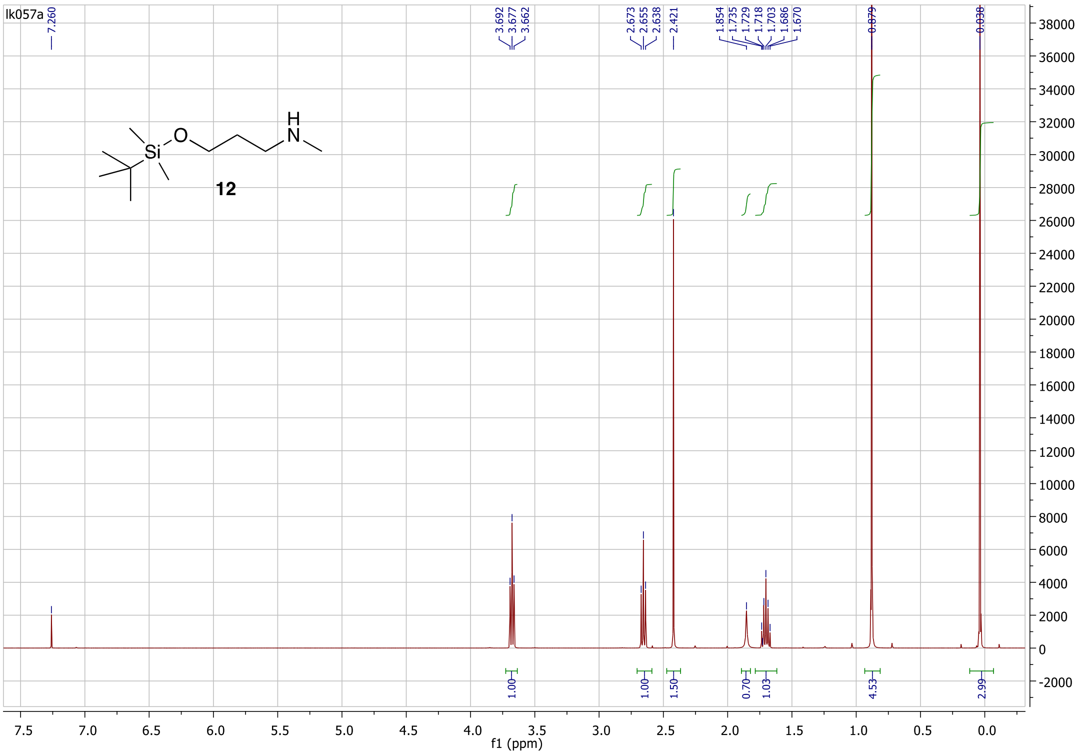

13C NMR

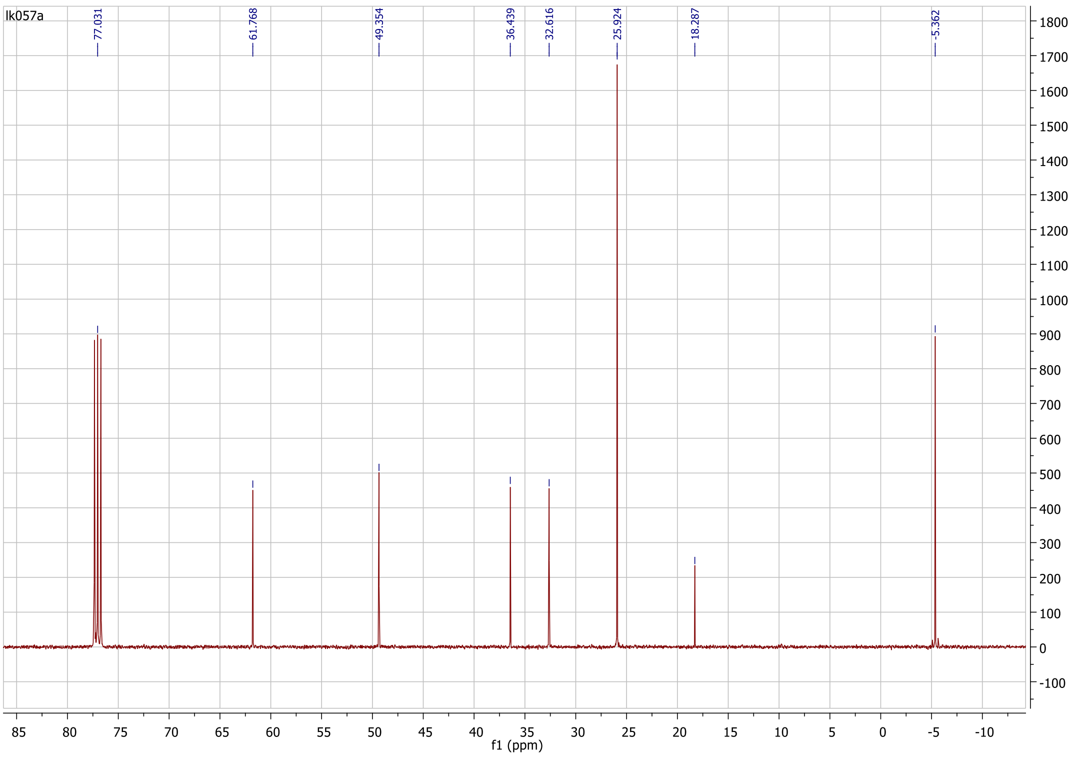

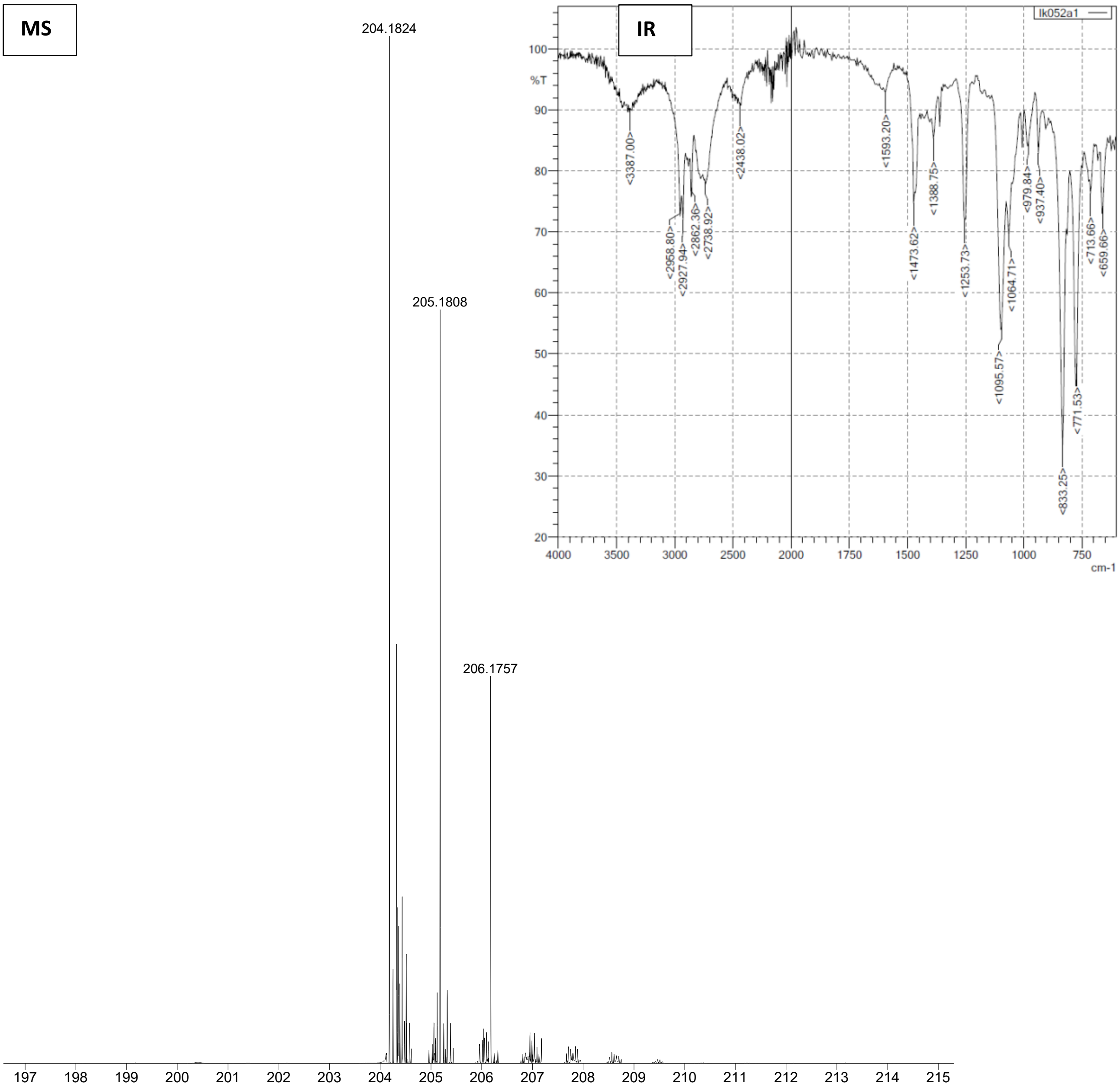
MS
204.1824
205.1808
206.1757
197 198 199 200 201 202 203 204 205 206 207 208 209 210 211 212 213 214 215
IR
lk052a1
%T
100 90 80 70 60 50 40 30 20
4000 3500 3000 2500 2000 1750 1500 1250 1000 750
cm-1
<3387.00>
<2958.80>
<2927.94>
<2862.36>
<2738.92>
<2438.02>
<1593.20>
<1473.62>
<1388.75>
<1253.73>
<1095.57>
<1064.71>
<979.84>
<937.40>
<833.25>
<771.53>
<713.66>
<659.66>

3,6-bis((3-((tert-butyldimethylsilyl)oxy)propyl)(methyl)amino)-9H-xanthen-9-one **13**

1H NMR

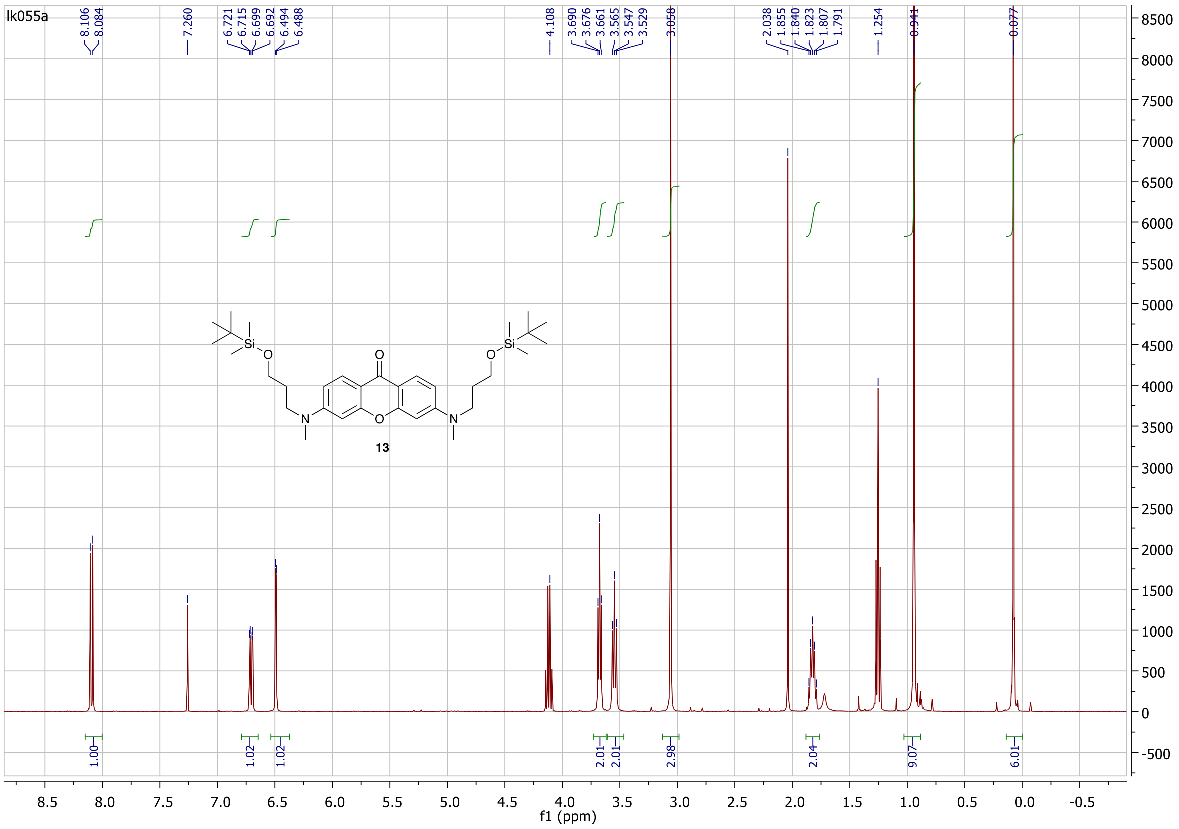

13C NMR

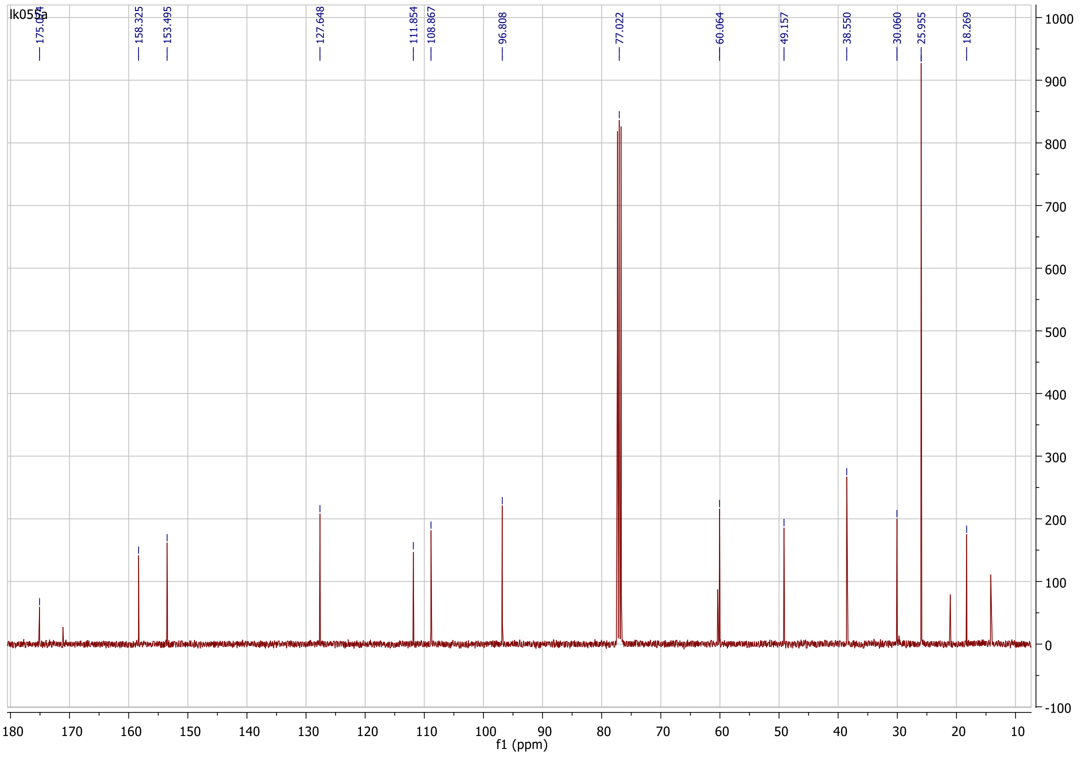

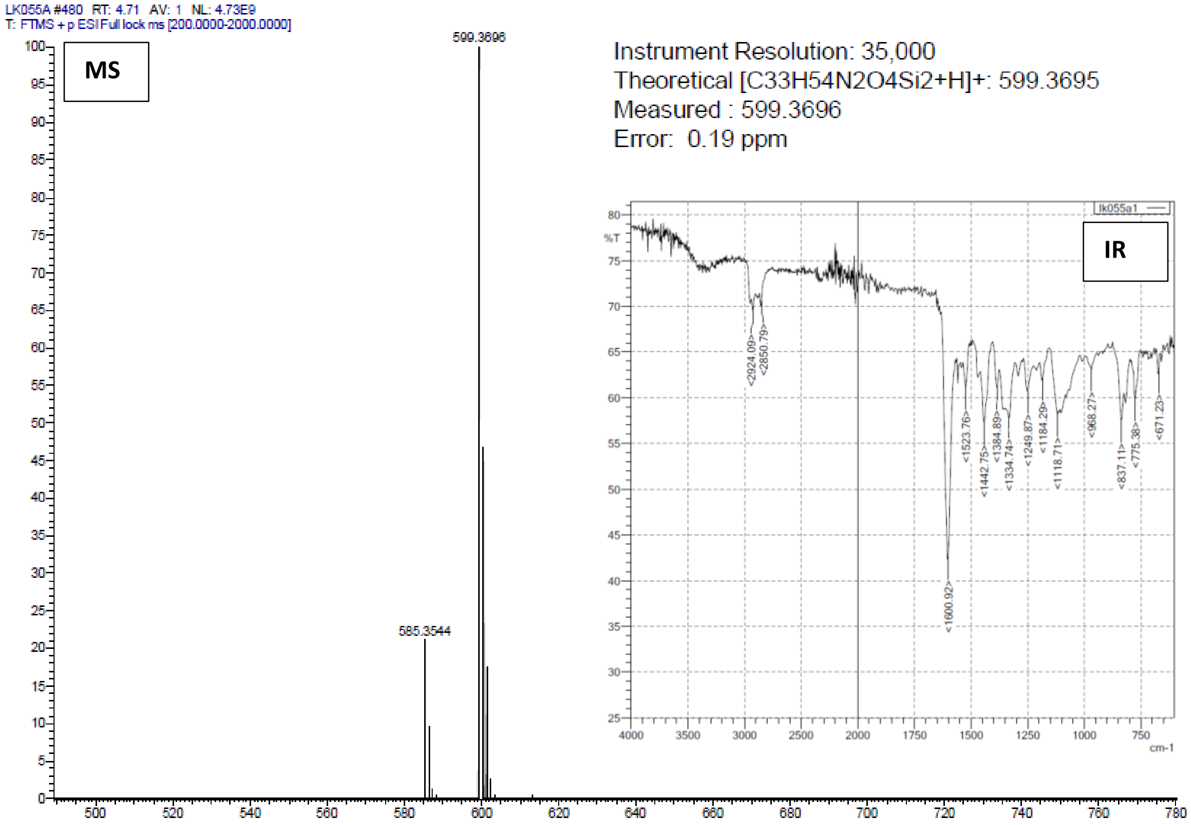
LK055A #480 RT: 4.71 AV: 1 NL: 4.73E9
T: FTMS + p ESI Full lock ms [200.0000-2000.0000]
MS
599.3696
585.3544
Instrument Resolution: 35,000
Theoretical [C33H54N2O4Si2+H]+: 599.3695
Measured : 599.3696
Error: 0.19 ppm
IR
lk055a1
%T
cm-1
<2924.09>
<2850.79>
<1600.92>
<1523.76>
<1442.75>
<1384.89>
<1334.74>
<1249.87>
<1184.29>
<1118.71>
<968.27>
<837.11>
<775.38>
<671.23>

(*E*)-3-((*tert*-butyldimethylsilyl)oxy)-*N*-(6-((3-((*tert*-butyldimethylsilyl)oxy)propyl)(methyl)amino)-9-(2,6-dimethoxyphenyl)-3H-xanthen-3-ylidene)-*N*-methylpropan-1-aminium chloride **14**

1H NMR

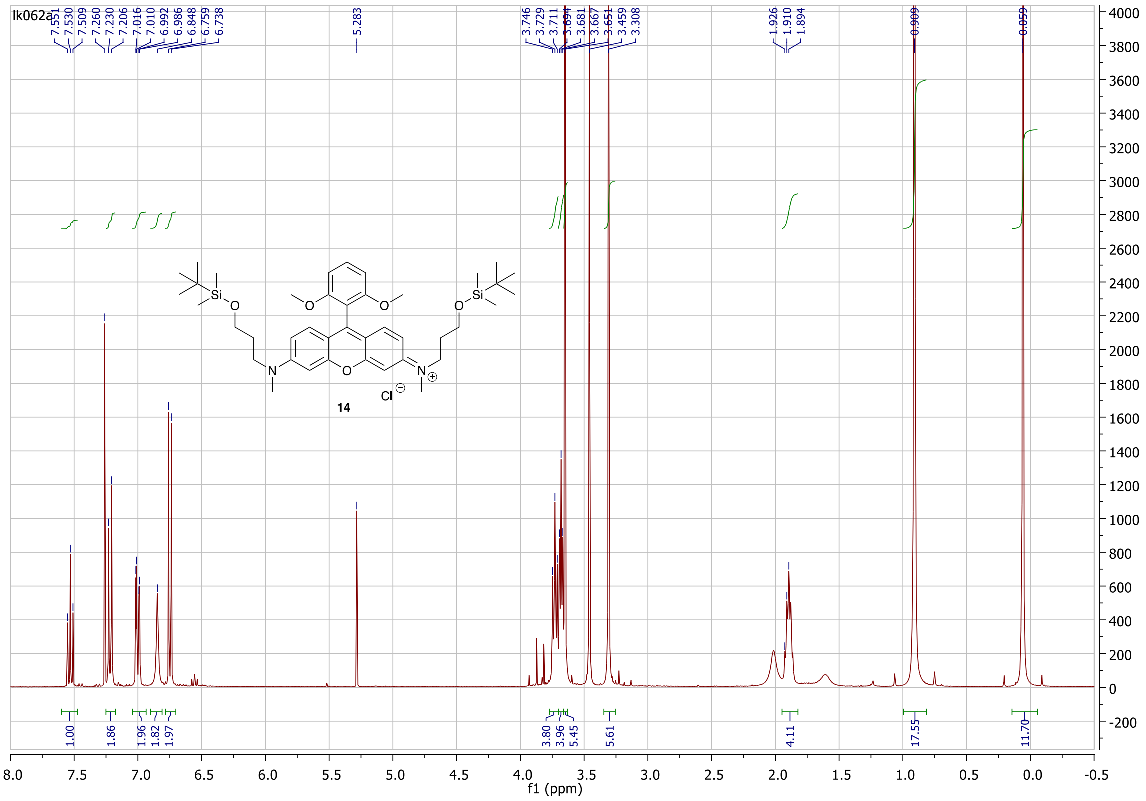

13C NMR

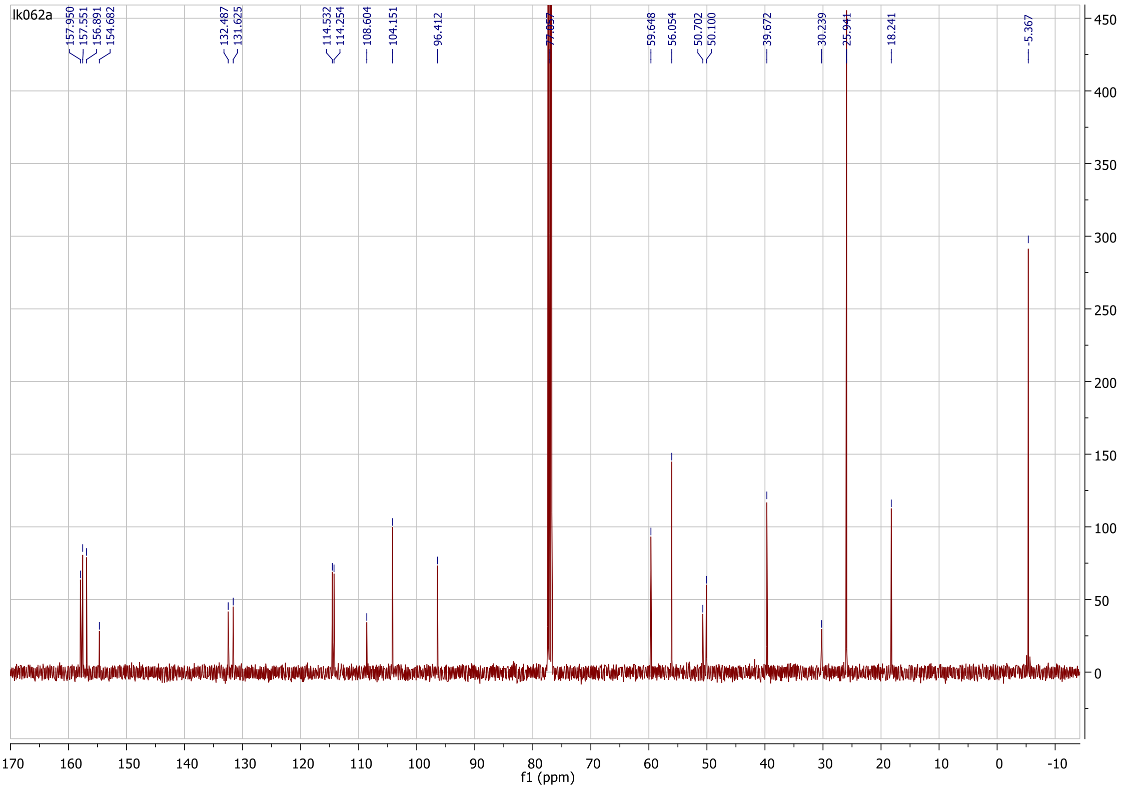

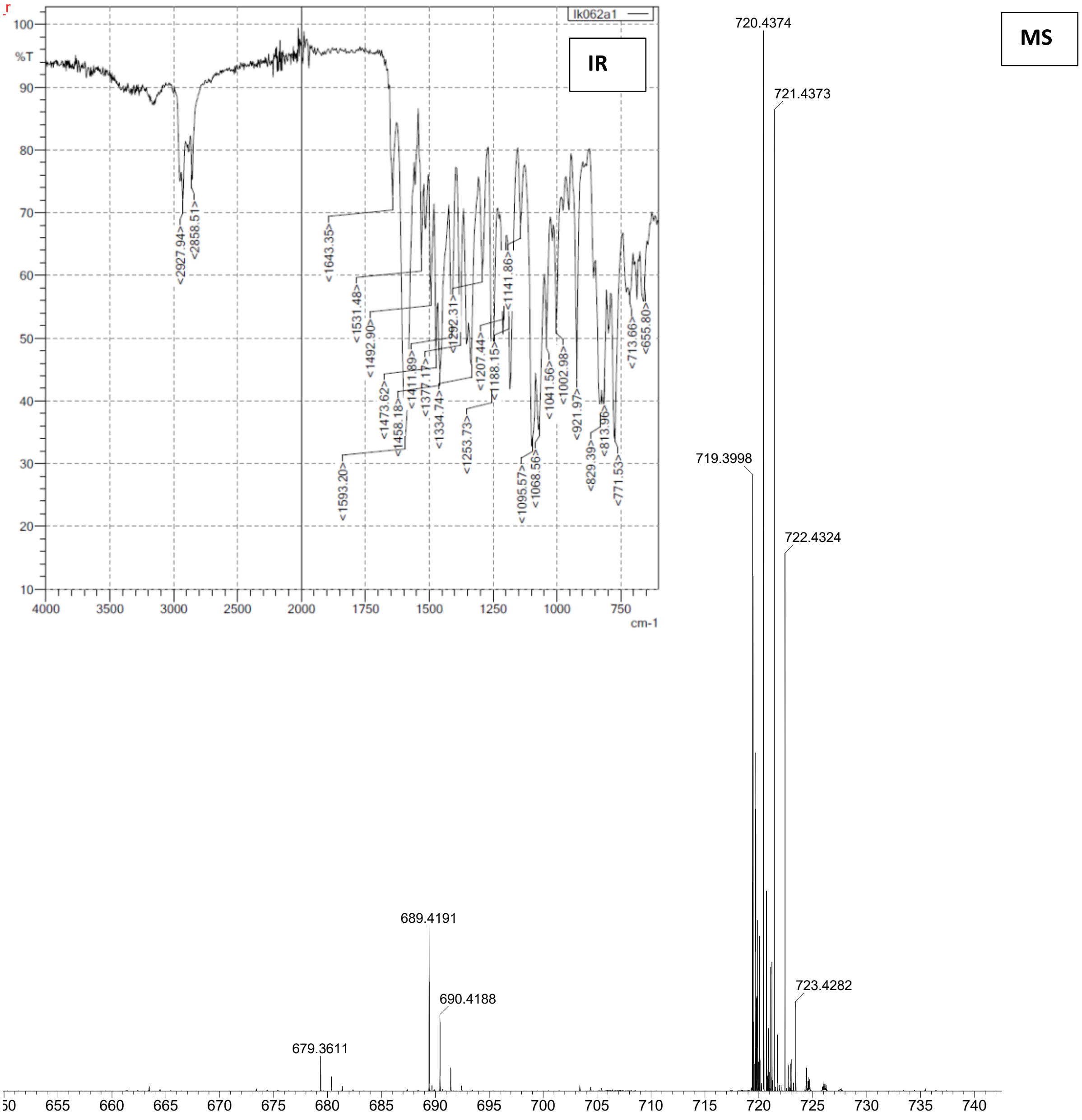
IR
lk062a1
%T
100
90
80
70
60
50
40
30
20
10
4000
3500
3000
2500
2000
1750
1500
1250
1000
750
cm-1
<2927.94>
<2858.51>
<1643.35>
<1593.20>
<1531.48>
<1492.90>
<1473.62>
<1458.18>
<1411.89>
<1377.17>
<1334.74>
<1292.31>
<1253.73>
<1207.44>
<1188.15>
<1141.86>
<1095.57>
<1068.56>
<1041.56>
<1002.98>
<921.97>
<829.39>
<813.96>
<771.53>
<713.66>
<655.80>
MS
720.4374
721.4373
719.3998
722.4324
723.4282
689.4191
690.4188
679.3611
655
660
665
670
675
680
685
690
695
700
705
710
715
720
725
730
735
740

(*E*)-*N*-(9-(2,6-dimethoxyphenyl)-6-((3-hydroxypropyl)(methyl)amino)-3H-xanthen-3-ylidene)-3-hydroxy-*N*-methylpropan-1-aminium chloride **15**

1H NMR

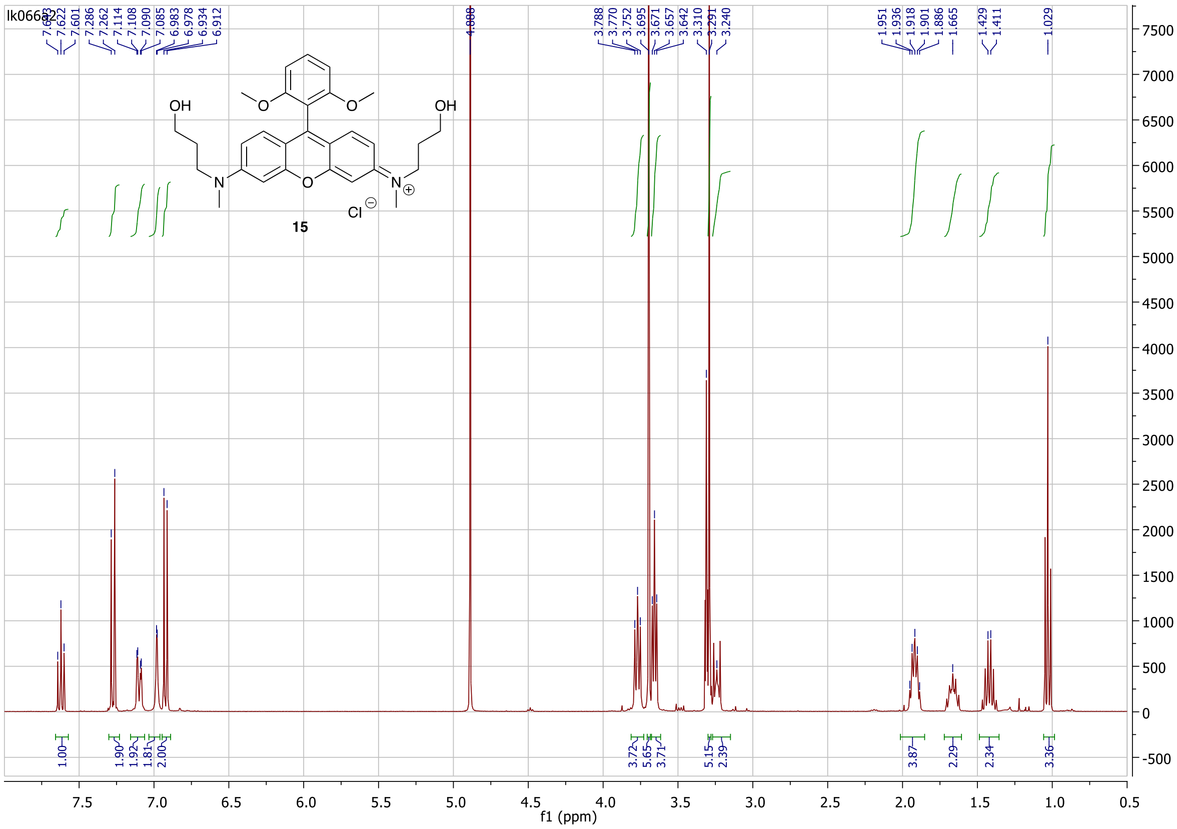

13C NMR

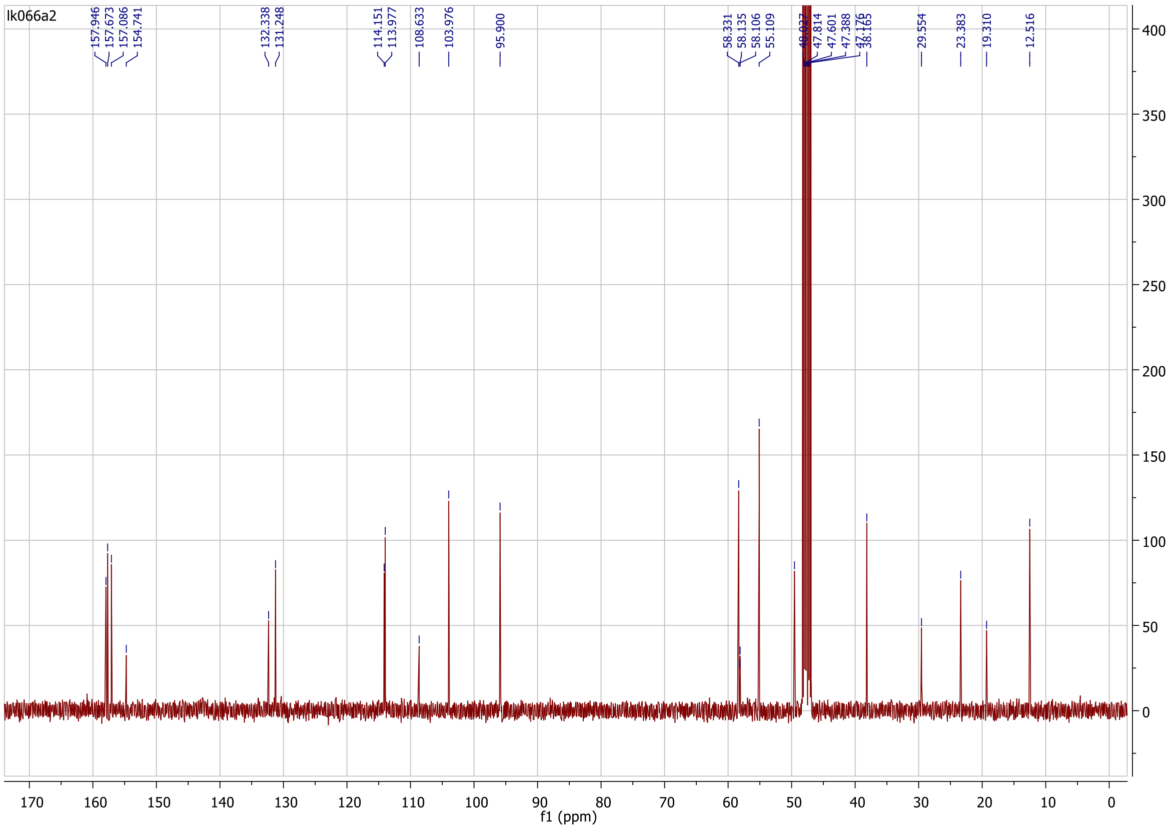

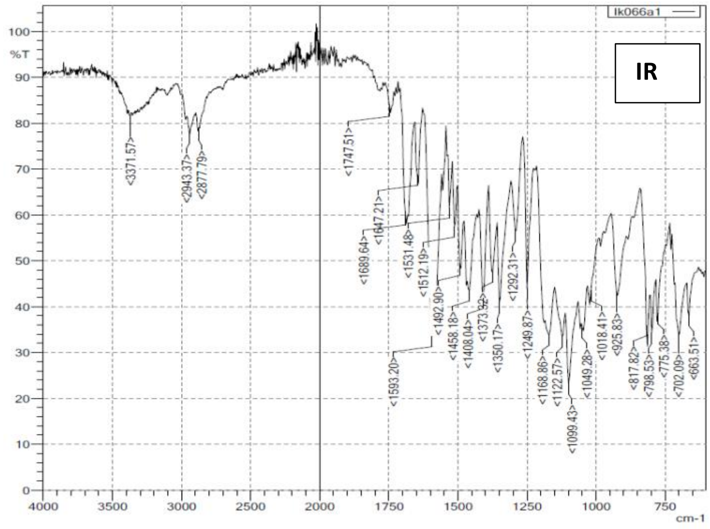

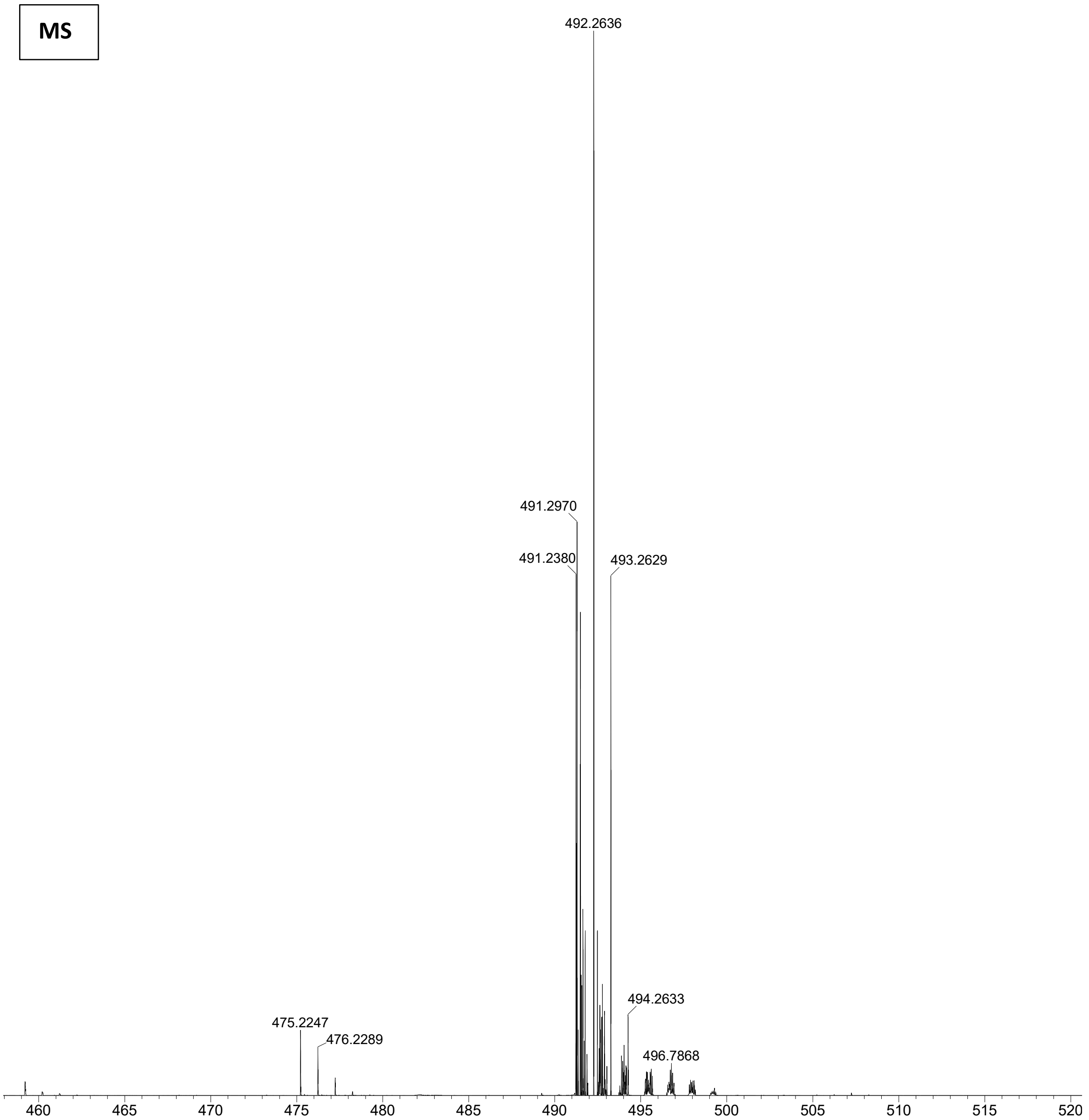

(*E*)-3-azido-N-(6-((3-azidopropyl)(methyl)amino)-9-(2,6-dimethoxyphenyl)-3H-xanthen-3-ylidene)-*N*-methylpropan-1-aminium iodide **4**

1H NMR

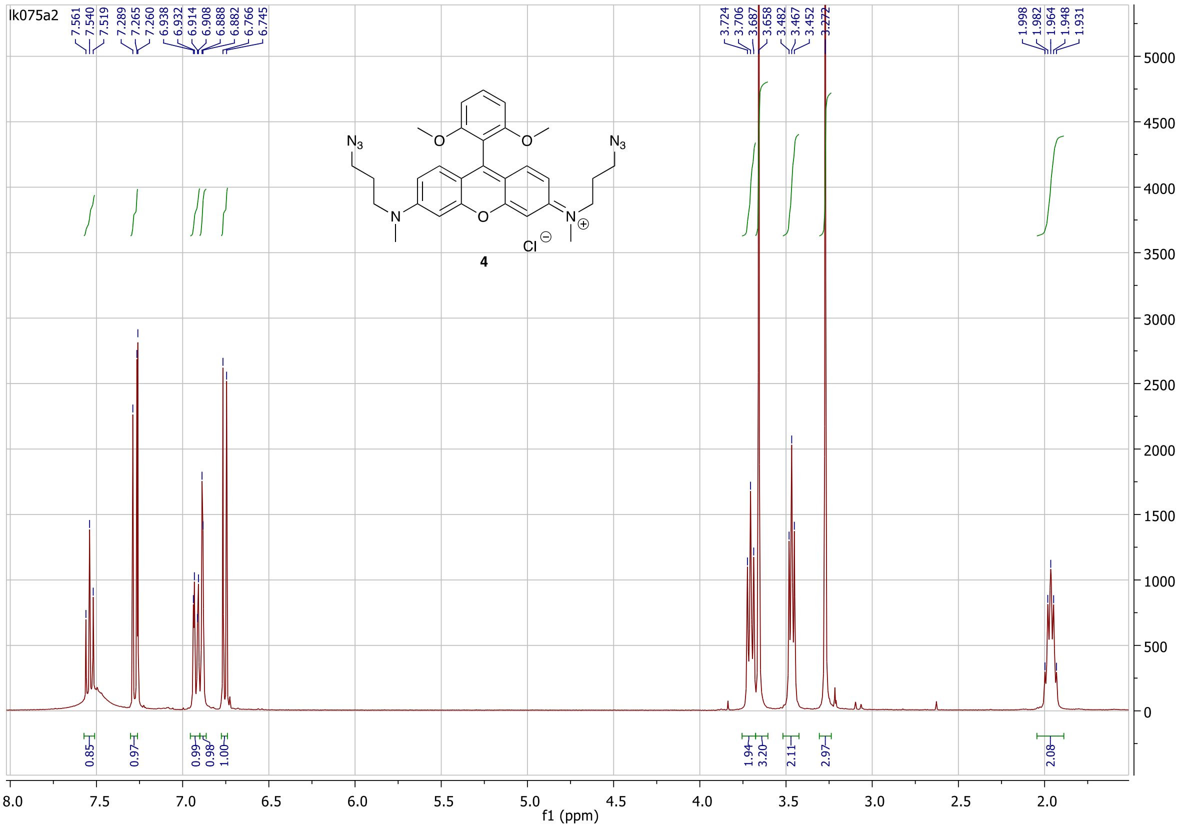

13C NMR

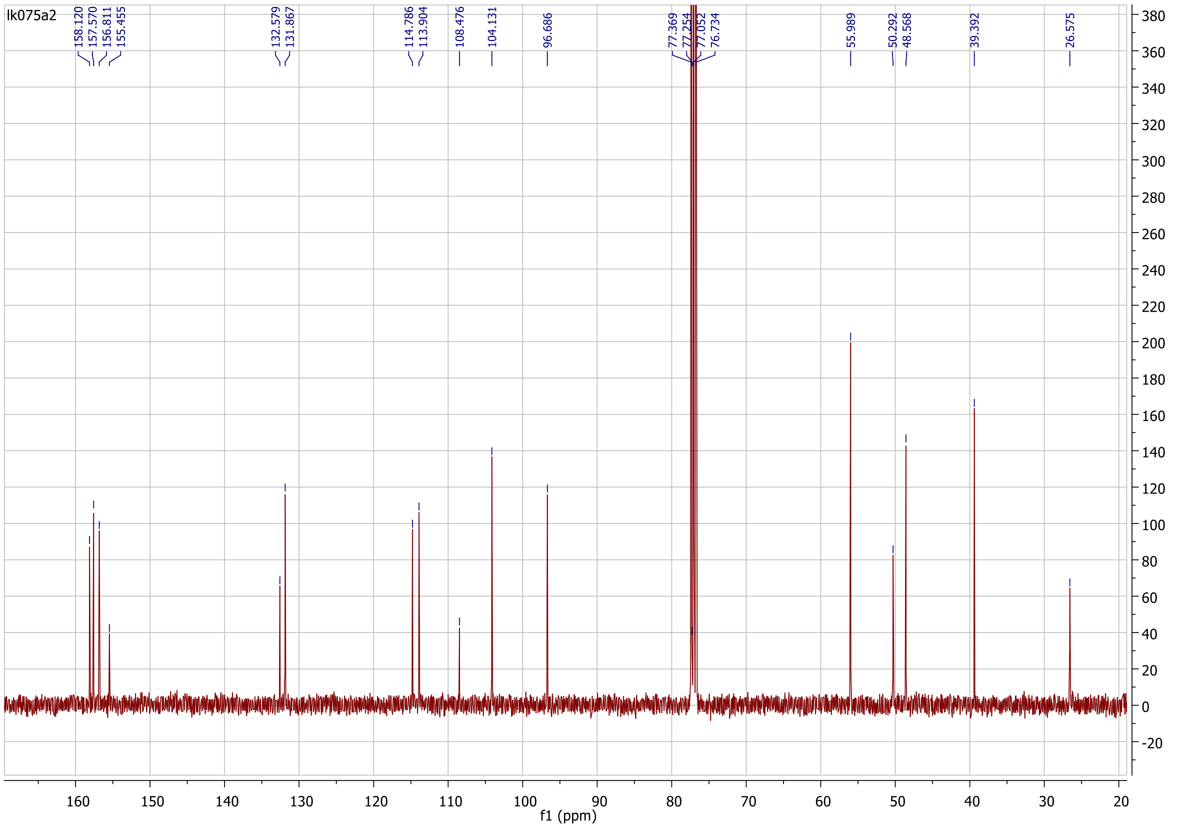

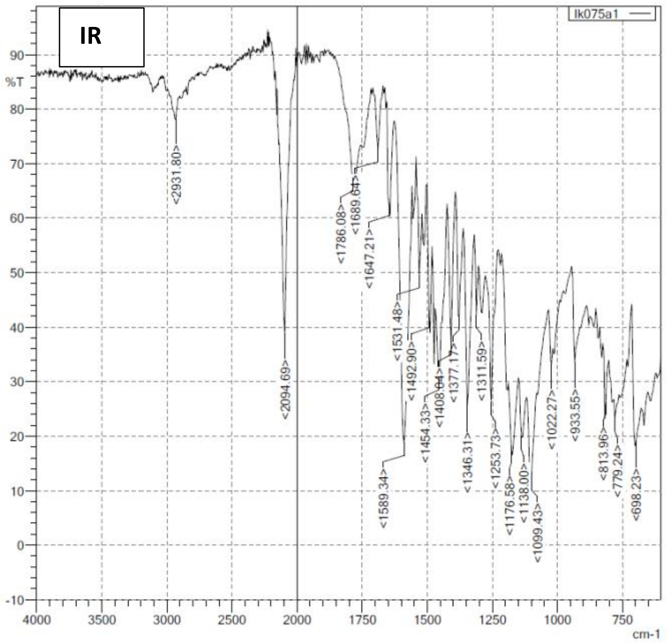
IR
lk075a1
%T
<2931.80>
<2094.69>
<1786.08>
<1689.64>
<1647.21>
<1589.34>
<1531.48>
<1492.90>
<1454.33>
<1377.17>
<1346.31>
<1311.59>
<1253.73>
<1176.58>
<1138.00>
<1099.43>
<1022.27>
<933.55>
<813.96>
<779.24>
<698.23>
cm-1

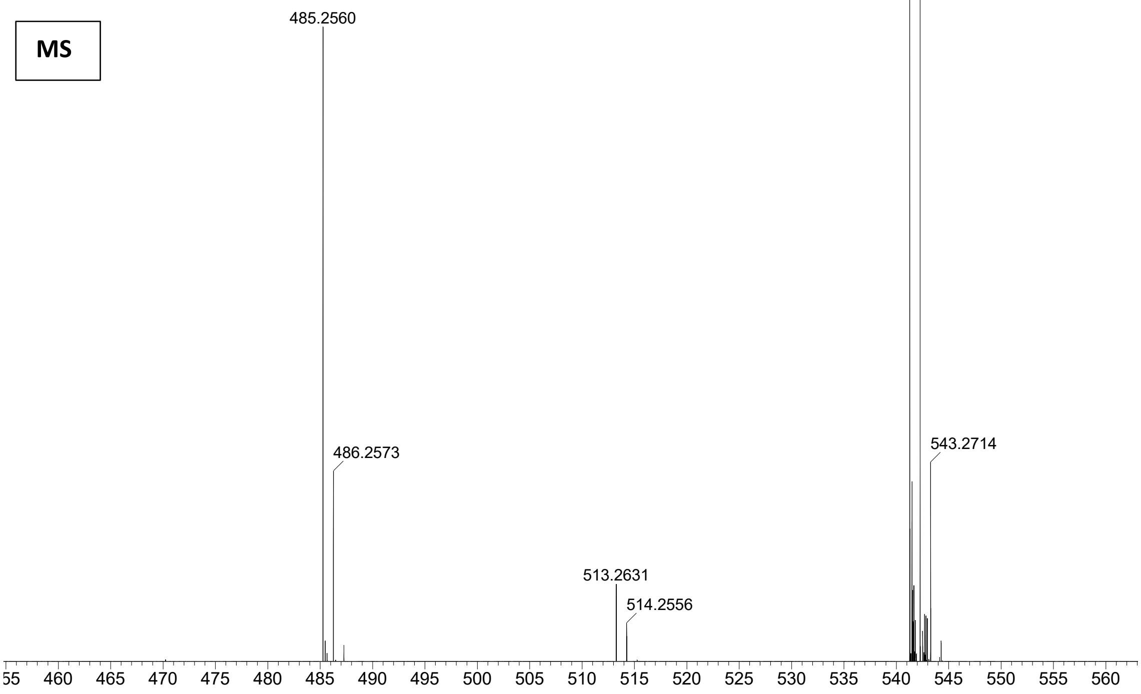
MS
485.2560
486.2573
513.2631
514.2556
541.2747
542.2750
543.2714

Supporting Information Biology

Peptide Labelling

Peptides corresponding to the C-helix of cardiac troponin C were custom-synthesized by ThermoFisher (>95% purity, TFA removal; **peptide I** sequence: NPTPCELQEMICEVDEDGS, **peptide II** sequence: NPTP[G]ELQEMI[G]EVDEDGS, **peptide III** sequence: NPTP[A]ELQEMI[A]EVDEDGS. ([G] – L-propargyl-glycine, [A] – L-azido-alanine,).

For thiol-reactive labelling, peptide 1 was solubilized in reaction buffer (50 mmol/L HEPES pH 7.5, 100 mmol/L NaCl and 50 mmol/L tris(2-carboxyethyl)phosphine) to a final concentration of 5 mmol/L. The peptide was further diluted to 50 μmol/L in the same buffer and bifunctional rhodamine (BSR) or bifunctional rosamine (BRos) were added to a final concentration of 50 μmol/L from a 50 mmol/L stock solution in DMSO. The reaction was incubated at room temperature for 60 mins and subsequently analyzed by reverse-phase chromatography on a C18 RP column using an Agilent 1100 HPLC system with detection wavelengths of 215 nm and 530 nm for the peptide backbone and fluorophore, respectively. Peptides were eluted with a linear gradient of 5% (v/v) to 100% (v/v) acetonitrile in 0.1% (v/v) trifluoro acetic acid.

For click reactions, peptides 2 and 3 were solubilized in 100 mmol/L potassium phosphate buffer pH 7, diluted to 50 mmol/L and equimolar amounts of either Azido-BRos or Propargyl-BRos added. The reaction was started by adding a pre-mixed solution of $CuSO_4$ and tris(3-hydroxypropyltriazolyl-methyl)amine (THPTA) with final concentrations of 0.1 mmol/L and 0.5 mmol/L, respectively, and a sodium ascorbate to final concentration of 5 mmol/L. Reactions were incubated for 60 mins at room temperature in the dark and analyzed by Reverse phase - HPLC as described above.

Samples were further analyzed by mass spectrometry on an Agilent 6130B Single Quadrupole Mass Spec w/ Agilent 1200 MS/LC System with electron spray ionization (ESI) (Agilent Technologies). Peptides were eluted with a linear gradient of 5% (v/v) to 100% (v/v) acetonitrile in 0.1% (v/v) formic acid.

Absorbance and fluorescence spectroscopy

Absorbance spectra of fluorophores were collected in 10 mmol/L Tris-HCl pH 7 in a Mettler Toledo UV-VIS Easy photo-spectrometer in quartz cuvettes after background correction. For probe concentrations >20 mml/L, the absorbance spectra were recorded on a ThermoFisher Nanodrop. Fluorescence spectra were collected in DMSO, methanol or 10 mmol/L Tris-HCl pH 7 a SpectraMax i3x plate reader using black 96-well plates with a resolution of 1 nm and bandwidth of 5 nm.

Molecular Modelling

Fluorophore structures were prepared in ChemDraw Suite 23 and energy minimized using the built-in MM2 force field calculations. Peptide structures were built in PyMol and linked to the fluorophore using built-in modelling functions. Conformers of the peptide-fluorophore complex were randomly sampled using the MMFF94 force field.

Supporting Information Figures

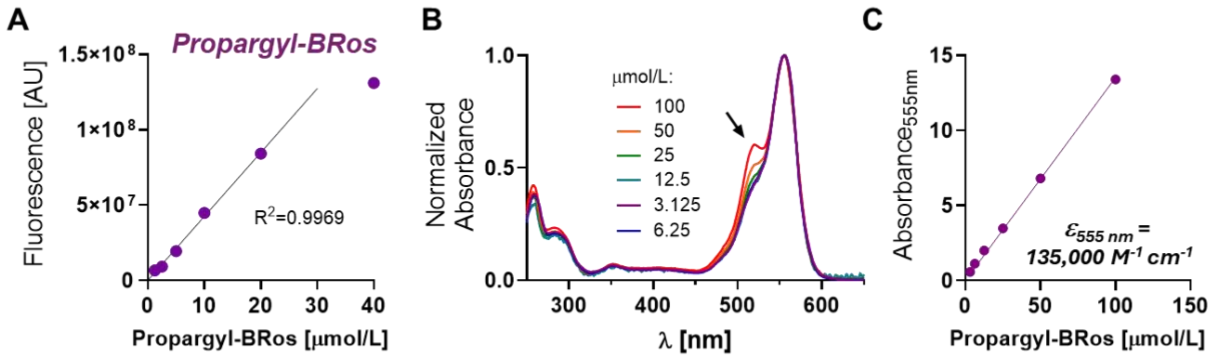

Supplemental Information Figure S1. (A) Concentration dependence of PrG-BRos fluorescence (exc 500 nm; em 580 nm). Linear regression to data points for <40 µmol/L is shown as a black continuous line (B) Normalized absorbance spectrum of PrG-BRos at different concentrations. The characteristic shoulder at 520 nm indicating fluorophore stacking is indicated by a black arrow. (C) Concentration-dependence of PrG-BRos absorbance at 555 nm and linear regression to data points (continuous line).

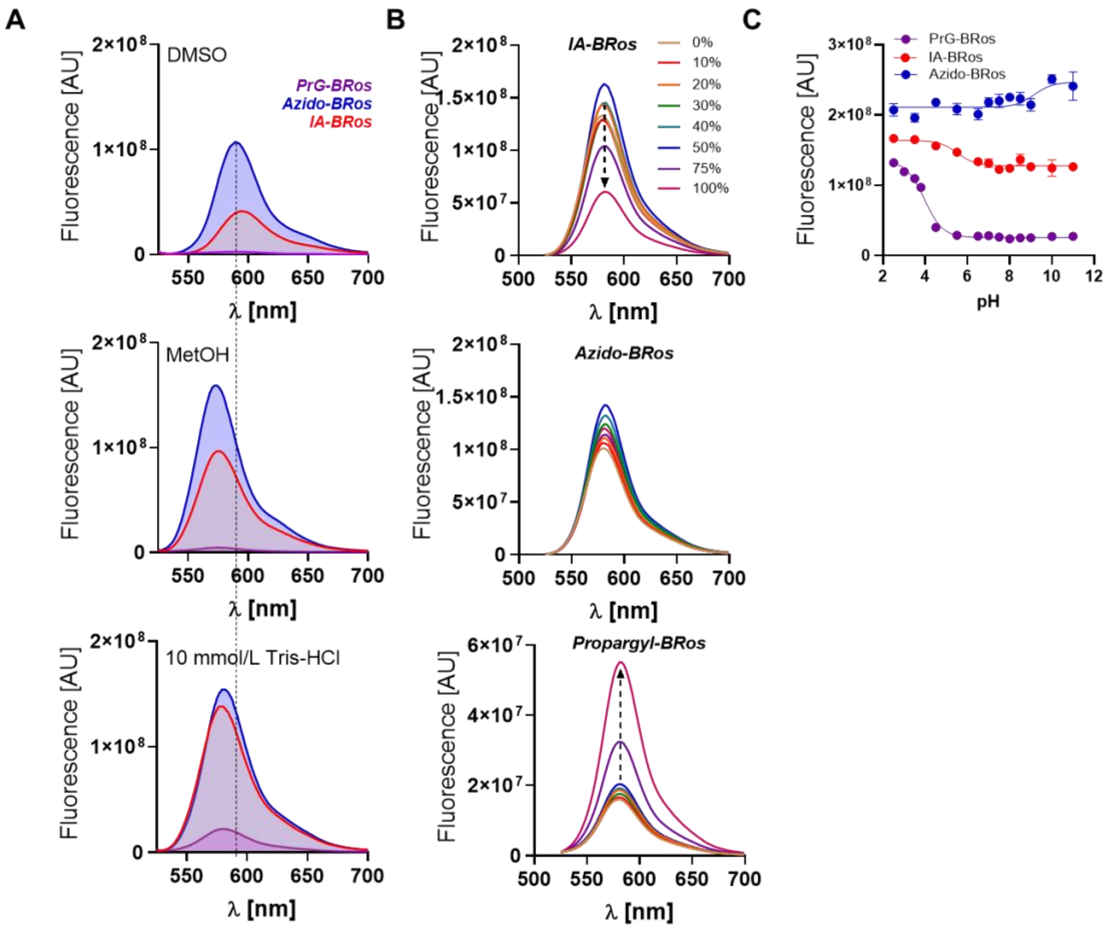

Supplemental Information Figure S2. (A) Emission spectrum of PrG-BRos (purple), Azido-BRos (blue) and IA-BRos (red) in DMSO, methanol and 10 mmo/L Tris HCl pH 7. (B) Emission spectrum of fluorophors at different glycerol concentrations % (v/v). (C) pH-dependence of fluorophore emission (exc 500 nm; em 580 nm).

Supporting Information Tables

Supporting Information Table S1. Summary of mass spectrometry analysis of peptides before and after labelling with IA-Bros, Azido-Bros and PrGßBRos.

	calculated mass [Da]	m/z found	species and calculated mass [Da]
unlabelled $(cys)_2$-peptide	2109.3	1055.4	$[M+2H^+]$ 1054.9
peptide crosslinked with IA-BRos	2674.5	892.0	$[M^++2H^+]$ 892.2
unlabelled $(PrG)_2$-peptide	2093.2	1048.6	$[M+2H^+]$ 1047.6
peptide crosslinked with Azido-BRos	2634.5	880.0	$[M^++2H^+]$ 878.8
unlabelled $(Azido)_2$-peptide	2127.2	1066.0	$[M+2H^+]$ 1064.6
peptide crosslinked with PrG-BRos	2688.5	897.8	$[M^++2H^+]$ 896.8

ESI of (a) unbound Peptide I and (b) bound to Bros-IA

(a)

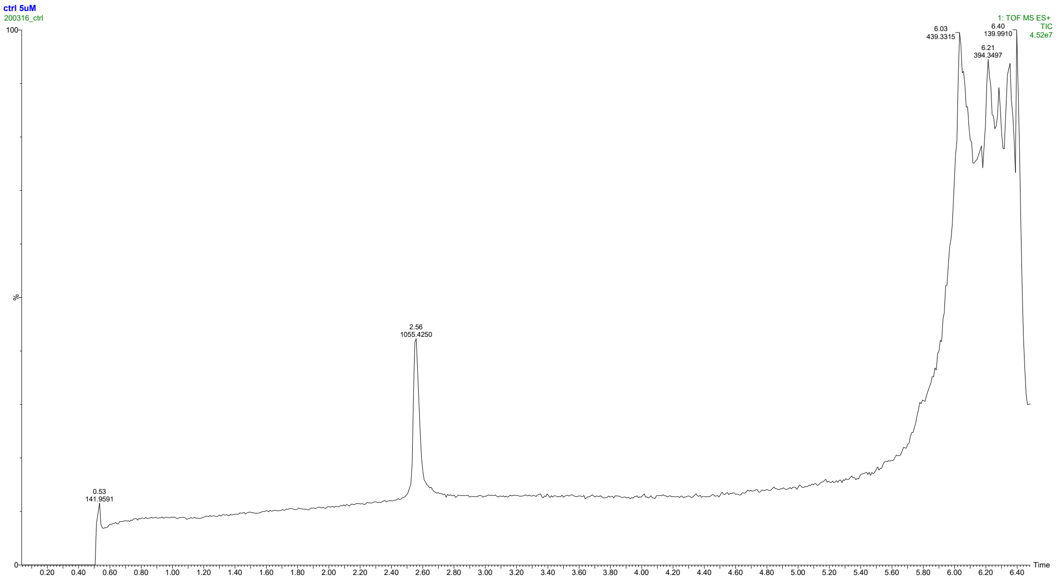

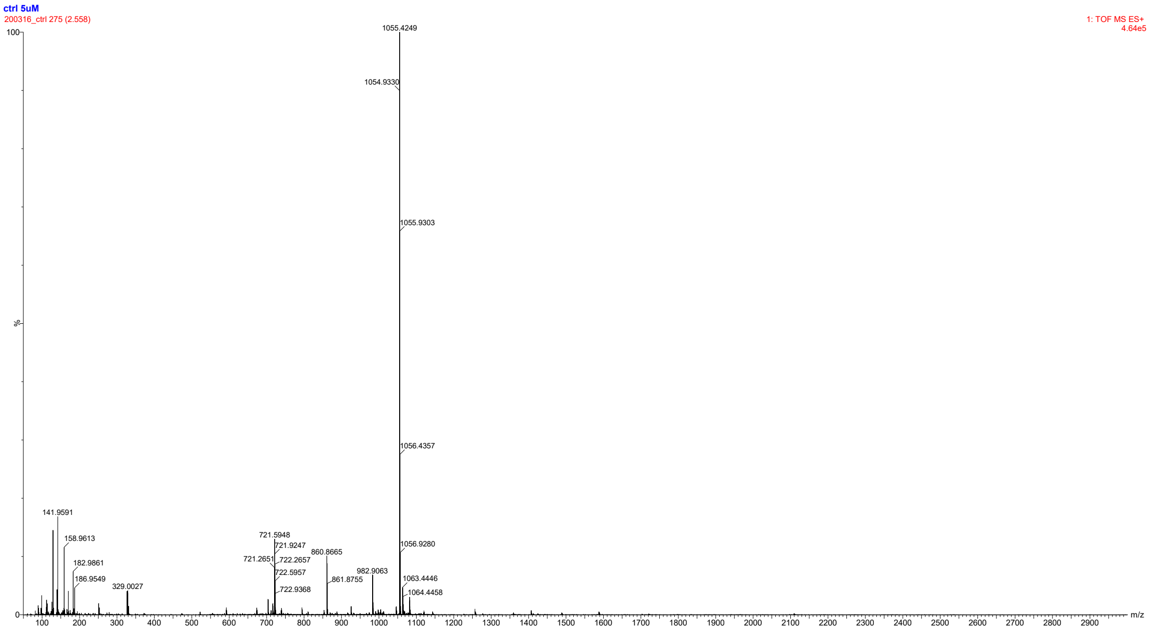

(b)

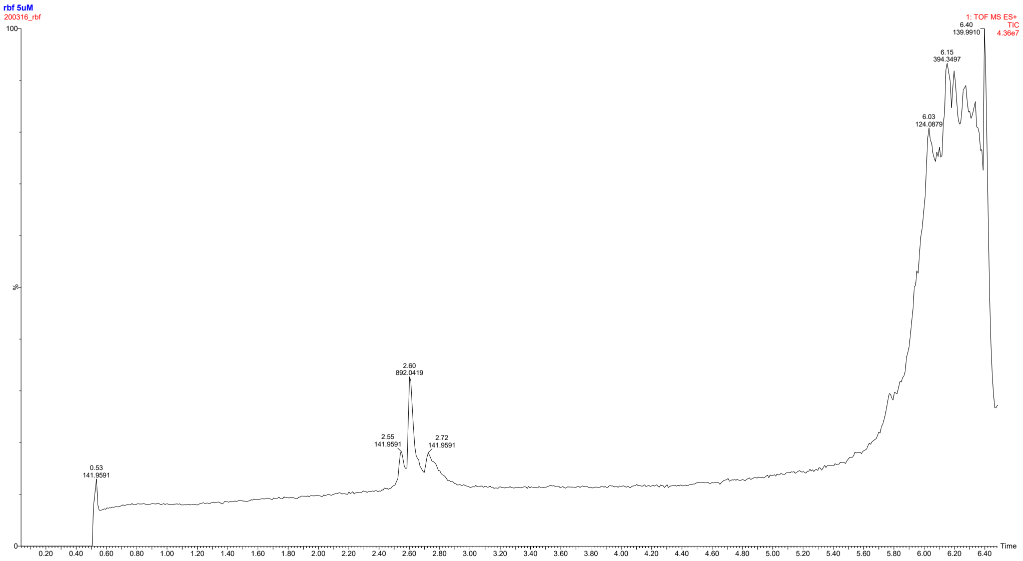
rbf 5uM
200316_rbf
1: TOF MS ES+
TIC
4.36e7
6.40
139.9910
6.15
394.3497
6.03
124.0879
2.60
892.0419
2.55
141.9591
2.72
141.9591
0.53
141.9591
Time

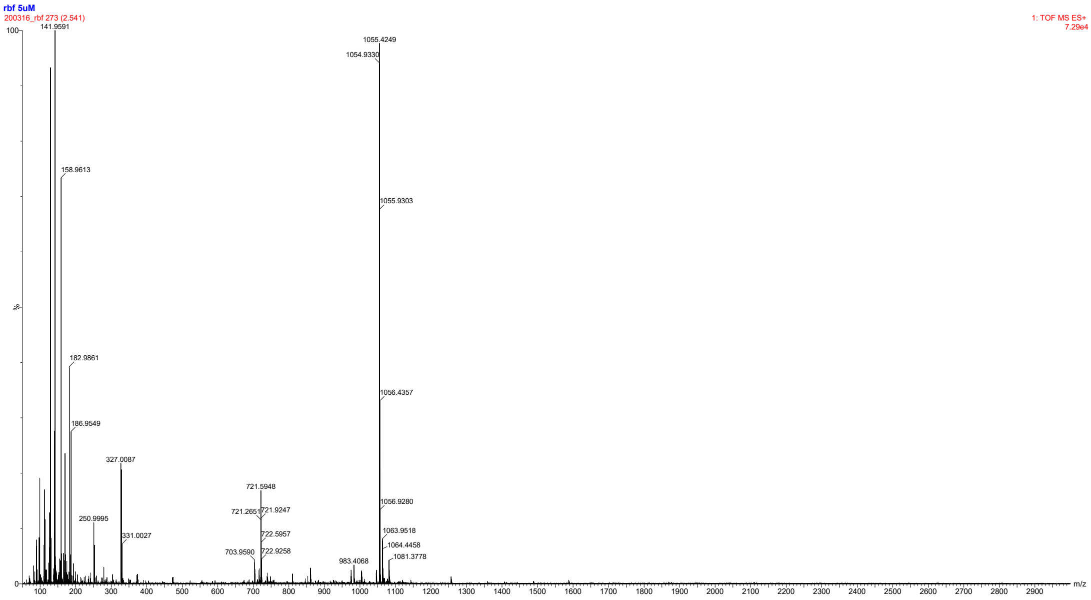
rbf 5uM
200316_rbf 273 (2.541)
1: TOF MS ES+
7.29e4
141.9591
158.9613
182.9861
186.9549
327.0087
250.9995
331.0027
721.5948
721.2651
721.9247
722.5957
703.9590
722.9258
983.4068
1055.4249
1054.9330
1055.9303
1056.4357
1056.9280
1063.9518
1064.4458
1081.3778
m/z

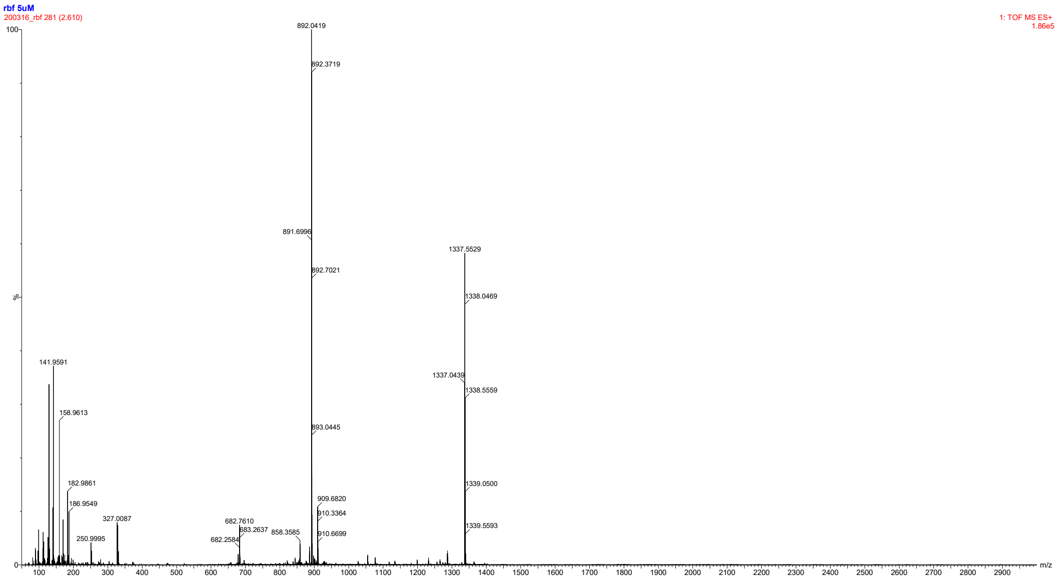
rbf 5uM
200316_rbf 281 (2.610)
1: TOF MS ES+
1.86e5
892.0419
892.3719
891.6996
892.7021
1337.5529
1338.0469
141.9591
1337.0439
1338.5559
158.9613
893.0445
182.9861
1339.0500
186.9549
909.6820
910.3364
327.0087
682.7610
683.2637
858.3585
1339.5593
910.6699
682.2584
250.9995
m/z

Supporting Information References

(1) Štacko, P.; Šebej, P.; Veetil, A. T.; Klán, P. Carbon-Carbon Bond Cleavage in Fluorescent Pyronin Analogues Induced by Yellow Light. *Org. Lett.* **2012**, *14* (18), 4918–4921. https://doi.org/10.1021/ol302244f.

(2) Wu, L.; Burgess, K. Synthesis and Spectroscopic Properties of Rosamines with Cyclic Amine Substituents. *J. Org. Chem.* **2008**, *73* (22), 8711–8718. https://doi.org/10.1021/jo800902j.

(3) Zhang, L.; Er, J. C.; Li, X.; Heng, J. J.; Samanta, A.; Chang, Y. T.; Lee, C. L. K. Development of Fluorescent Probes Specific for Parallel-Stranded G-Quadruplexes by a Library Approach. *Chem. Commun.* **2015**, *51* (34), 7386–7389. https://doi.org/10.1039/c5cc01601k.

(4) Pisoni, D. S.; Todeschini, L.; Borges, A. C. A.; Petzhold, C. L.; Rodembusch, F. S.; Campo, L. F. Symmetrical and Asymmetrical Cyanine Dyes. Synthesis, Spectral Properties, and Bsa Association Study. *J. Org. Chem.* **2014**, *79* (12), 5511–5520. https://doi.org/10.1021/jo500657s.

(5) Baumann, L.; Schöller, K.; De Courten, D.; Marti, D.; Frenz, M.; Wolf, M.; Rossi, R. M.; Scherer, L. J. Development of Light-Responsive Porous Polycarbonate Membranes for Controlled Caffeine Delivery. *RSC Adv.* **2013**, *3* (45), 23317–23326. https://doi.org/10.1039/c3ra44399j.

(6) Collot, M.; Kreder, R.; Tatarets, A. L.; Patsenker, L. D.; Mely, Y.; Klymchenko, A. S. Bright Fluorogenic Squaraines with Tuned Cell Entry for Selective Imaging of Plasma Membrane vs. Endoplasmic Reticulum. *Chem. Commun.* **2015**, *51* (96), 17136–17139. https://doi.org/10.1039/c5cc06094j.
